# A Six‐Crossing Doubly Interlocked [2]Catenane with Twisted Rings, and a Molecular Granny Knot

**DOI:** 10.1002/anie.201807135

**Published:** 2018-09-19

**Authors:** Jonathan J. Danon, David A. Leigh, Simone Pisano, Alberto Valero, Iñigo J. Vitorica‐Yrezabal

**Affiliations:** ^1^ School of Chemistry University of Manchester Oxford Road Manchester M13 9PL UK

**Keywords:** catenanes, chemical topology, coordination chemistry, molecular grids, supramolecular chemistry

## Abstract

A molecular 623 link (a six crossing, doubly interlocked, [2]catenane with twisted rings) and a 3_1_#3_1_ granny knot (a composite knot made up of two trefoil tangles of the same handedness) were constructed by ring‐closing olefin metathesis of an iron(II)‐coordinated 2×2 interwoven grid. The connections were directed by pendant phenyl groups to be between proximal ligand ends on the same faces of the grid. The 623 link was separated from the topoisomeric granny knot by recycling size‐exclusion chromatography. The identity of each topoisomer was determined by tandem mass spectrometry and the structure of the 623 link confirmed by X‐ray crystallography, which revealed two 82‐membered macrocycles, each in figure‐of‐eight conformations, linked through both pairs of loops.

Links (generally termed “catenanes” when referring to molecular systems) are mechanically connected closed loops (macrocycles).^1^ Complex links are found in some proteins^2^ and DNA.^3^ Topologies with two or three small‐molecule components that have succumbed to synthesis include Hopf links^4^ (the simplest [2]catenane topology; a 221 link in Alexander–Briggs notation^5^), Solomon links (421 ),^6^ a Star of David catenane (621 ),^7^ Borromean rings (632 ),^8^ and 633 9 and 937 10 links.^11^ The topologies with two components and fewer than seven crossings that have yet to be prepared in small‐molecule form are the Whitehead link (521 ),^12^ and 622 and 623 links (types of two‐component links with twists in the rings).^13^ Most of the complex molecular links synthesized to date are derived from metal coordination complexes that assemble ligand strands in spatial arrangements that direct the ring‐closing reactions to generate the required topology.^14^ Types of motifs employed include orthogonally positioned ligand complexes,^15^ linear^16^ and circular^17^ metal helicates, and interwoven molecular grids.6m A 2×2 interwoven grid was previously used to synthesize a Solomon link by connecting the ends of parallel ligand strands.6m However, the possibility of accessing different topologies from alternative ligand connectivities using similar types of scaffold remained unexplored. Herein we report on the designed synthesis of one of the topologies missing from the molecular lexicon, a 623 link, via a 2×2 interwoven grid. Also formed is a 3_1_#3_1_ 18 (granny) composite^10^, ^19^ knot.

The 623 link^13^ consists of two constitutionally identical macrocycles involving six crossings, the same number as a Star of David catenane (621 link) but with a different crossing pattern. The topology can be depicted as two figure‐of‐eight shapes linked through both pairs of loops (Figure 1). The crossing pattern for this link can, in principle, be achieved from a 2×2 interwoven grid by connecting the ends of ligand strands on the same face of the grid that are coordinated to adjacent metal ions. However, if the grid is a near‐perfect square then each strand end is equidistant to the two closest ligand‐ends and a mixture of two topoisomers will be obtained. The relative orientation of the connections on the two faces of the grid plane dictates the topology formed: a 623 link (the joined ends on one face are roughly perpendicular to the joined ends on the other face) or a granny knot (the connected groups on each face are close to parallel with those on the other) (Figure 1).

**Figure 1:**
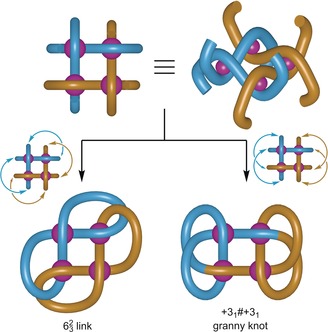
The topologies obtained by connecting the closest ligand ends on the same face of a 2×2 interwoven square grid. The knot and link topologies are both chiral,^1^, ^14^, ^18^ formed as a racemic mixture from a racemic grid.

To achieve the required connectivity of the ligand strands, we designed bis‐tridentate ligand **1** based on the bis(benzimidazolylpyridyl)thiazolo[5,4‐d]thiazole scaffold previously used to assemble a Solomon link (Figure 2).6m The *meta*‐substituted phenyl groups (attached as part of a biphenyl unit) direct the pendant alkene chains above or below the grid plane, the biphenyl linkage significantly restricting the space accessible by the chain ends. The chain length was chosen to avoid over‐reaching, permitting only the required strand connections. Ethyl groups were used to increase the solubility of the benzimidazole units. These are smaller than the isopentyl groups used in the Solomon link synthesis6m to avoid sterically hindering the conformations of the alkene chains that lead to the required connections.

**Figure 2:**
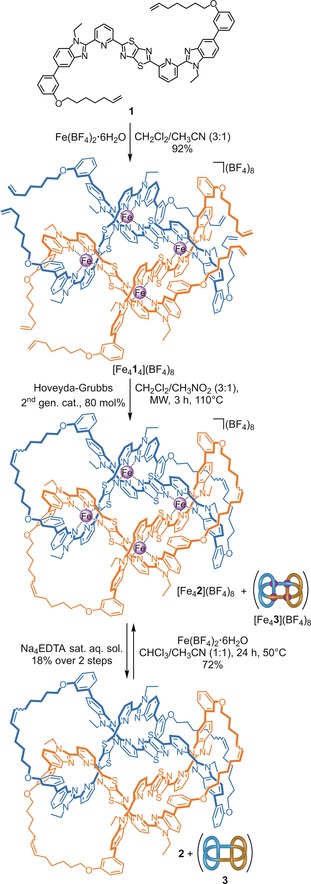
Metal‐directed synthesis of 623 link **2** and granny knot **3** from ligand **1**.

To confirm that the modifications of the ligand would not hamper grid formation, we prepared a model ligand with methyl groups in place of the alkene chains (Supporting Information, Section 2.5). Complexation of the ligand with an equimolar amount of iron(II) or zinc(II) tetrafluoroborate proceeded smoothly in a mixture of dichloromethane and acetonitrile at room temperature to quantitatively afford the corresponding 2×2 interwoven grids, which were characterised by NMR spectroscopy and X‐ray crystallography (Supporting Information, Sections 2.5 and 3). The alkene‐bearing 2×2 interwoven grid [Fe_4_ **1**_4_](BF_4_)_8_ was synthesized in analogous fashion, using ligand **1** and a small excess of iron(II) tetrafluoroborate (Supporting Information, Section 2.2). The ^1^H NMR spectrum of [Fe_4_ **1**_4_](BF_4_)_8_ showed significant downfield shifts of the pyridine protons compared to the parent ligand (**1**), and a significant upfield shift of the benzimidazole proton H^s^ (Supporting Information, page 9 and Figure S55), which should face the pyridine ring of the perpendicular ligand strand in [Fe_4_ **1**_4_](BF_4_)_8_ and therefore is indicative of the anticipated grid structure.

The 2×2 interwoven grid [Fe_4_ **1**_4_](BF_4_)_8_ was subjected to ring‐closing olefin metathesis (RCM) as a dilute solution (1 mm) in dichloromethane/nitromethane (3:1), employing the Hoveyda–Grubbs second generation catalyst^20^ under microwave heating in a sealed vial (Figure 2). After 3 h, ^1^H NMR indicated complete consumption of the starting material, and electrospray ionization mass spectrometry (ESI‐MS) showed multiply charged peaks corresponding to the successive loss of tetrafluoroborate counteranions from the fully‐closed grid (Supporting Information, Figure S3). The crude product was demetallated (Figure 2) by treatment with a saturated aqueous solution of tetrasodium ethylenediaminetetraacetate (Na_4_EDTA) to afford a mixture of the metal‐free knot and link and other reaction byproducts, which were separated by preparative recycling size‐exclusion chromatography (GPC) (Supporting Information, Sections 2.2 and 2.3).

The non‐interlocked macrocycle and other, non‐ring‐closed, compounds obtained from the RCM reaction were removed from the knot and link in the first GPC cycles to give pristine **2**/**3** in 18 % overall yield. However, the separation of the two topoisomers, which appeared as a single peak during the first few GPC cycles, proved to be far more challenging. After exhaustive recycling (15–17 cycles), two distinct peaks were obtained, the identities of which were determined by tandem ESI‐MS. The faster eluting compound gave a series of tandem MS peaks (Figure 3 b) that correspond to linear fragments that can only arise from the composite knot (fragmentation of the same bonds in a link would lead to dethreading and lower masses corresponding to the intact charged component macrocycle^21^). Under the same conditions, the equivalent ion of the slower eluting fraction fragmented to singly and doubly charged macrocycle without any higher mass fragments, behaviour only consistent with a link topology (Figure 3 a). Both topoisomers were also characterised by ^1^H and ^13^C NMR, giving distinct but complex spectra (Supporting Information, Section 2.3). The broad ^1^H NMR spectra are typical of slow reptation of interwoven strands in large and complex molecular knots and links (Supporting Information, Figure S10).^7^, ^22^, ^23^

**Figure 3:**
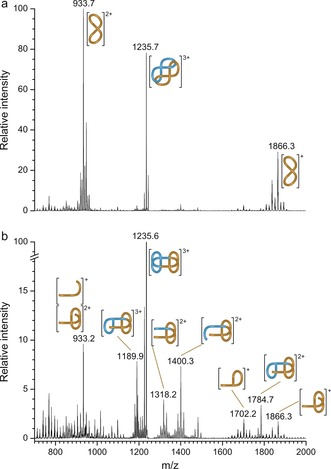
ESI‐MS/MS tandem mass spectra of the [M+3H]^3+^ ion for a) 623 link **2** and b) granny knot **3**.

The individual topoisomers were each remetallated by treatment with excess iron(II) tetrafluoroborate in a chloroform/acetonitrile mixture under heating (Figure 2 and Supporting Information, Section 2.4). In each case this proceeded with full conversion to the corresponding metallated complex, [Fe_4_ **2**](BF_4_)_8_ or [Fe_4_ **3**](BF_4_)_8_ respectively. The ^1^H NMR spectrum of each complex was significantly sharper than that of the mixture originally obtained from the RCM reaction (Supporting Information, Figures S2 and S40).

We attempted to grow crystals from a sample of [Fe_4_ **2**/**3**](BF_4_)_8_ obtained by remetallation of a mixture of the organic topoisomers that had been separated from other species by GPC (Supporting Information, Section 2.2). However, slow diffusion of toluene into a solution of [Fe_4_ **2**/**3**](BF_4_)_8_ in acetone yielded single crystals of, to our surprise, the demetallated 623 link. The coordination of thiazolo[5,4‐d]thiazole ligands to Fe^II^ is rather weak6m and it appears that the poor solubility of metal‐free **2** causes it to crystallize from solution as it reversibly forms at very low concentration. The X‐ray crystal structure of **2** (Figure 4 and Supporting Information, Section 3) shows two 82‐membered macrocycles, each with one twist, mechanically joined through both pairs of loops to form the six‐crossing 623 link topology (Figure 4). A circa 3:1 *E*/*Z* mixture of alkenes is present in the alkyl chains. The aromatic regions of the ligand strands stack at the centre of the structure, with an average distance of about 3.5 Å between strands. From inspection of models it is clear that the 623 link is topologically chiral;^1^, ^18^ both enantiomers are present in the unit cell.

**Figure 4:**
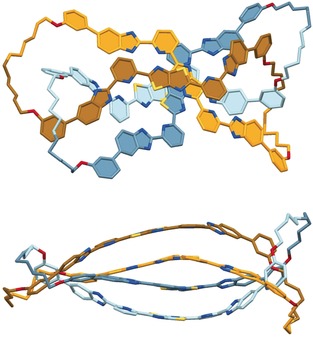
X‐Ray crystal structure of the metal‐free 623 link (**2**).^25^ Top view (top) and side view (bottom) showing the stacking of the ligand strands. Carbon atoms are coloured according to the macrocycle (the light blue and orange regions run below their darker blue and brown counterparts); N blue, O red, S yellow. A single enantiomer is shown; both enantiomers are present in the unit cell. The hydrogen atoms, pendant ethyl groups, and solvent molecules are omitted for clarity.

The designed assembly of different molecular topologies is one of the last forms of isomerism still to be properly mastered through synthetic chemistry. Like natural product synthesis,^24^ the way the field is advanced is to demonstrate the synthesis of previously difficult to make or inaccessible structures. In doing so a toolbox of strategies, tactics, and methods is developed that can be used to tackle ever more complex targets. The current work demonstrates that carefully designed ligand extensions can be used to direct the end‐groups of interwoven strands on a 2×2 grid for connections that generate both a six‐crossing two‐component prime link and a granny composite knot.

## Conflict of interest

The authors declare no conflict of interest.

## Supporting information

As a service to our authors and readers, this journal provides supporting information supplied by the authors. Such materials are peer reviewed and may be re‐organized for online delivery, but are not copy‐edited or typeset. Technical support issues arising from supporting information (other than missing files) should be addressed to the authors.

Supplementary

Supplementary
