# Supplementary material for: A Six‐Crossing Doubly Interlocked [2]Catenane with Twisted Rings, and a Molecular Granny Knot
Source: Angew Chem Int Ed Engl. 2018 Sep 19;57(42):13833–7. doi: 10.1002/anie.201807135 (PMC6221036; doi:10.1002/anie.201807135)
Supplement: Supplementary file 1 — Supplementary [file ANIE-57-13833-s001.pdf]

Angewandte Chemie
Eine Zeitschrift der Gesellschaft Deutscher Chemiker

Supporting Information

A Six-Crossing Doubly Interlocked [2]Catenane with Twisted Rings, and a Molecular Granny Knot

Jonathan J. Danon, David A. Leigh, Simone Pisano, Alberto Valero, and Iñigo J. Vitorica-Yrezabal*

anie_201807135_sm_miscellaneous_information.pdf
anie_201807135_sm_Movie.mp4

Table of Contents

Table of Contents 2
1. General experimental section 3
2. Synthesis and experimental 4
  2.1. Synthesis of ligand **1** 4
  2.2. Synthesis of molecular grid [Fe_4**1**$_4$]$(BF_4)_8$, 6_3^2 link **2** and granny knot **3** 8
  2.3. GPC separation of topoisomers **2** and **3** 19
  2.4. Remetallation of pure topoisomers **2** and **3** 36
  2.5. Synthesis of the model ligand and model grids 40
3. X-ray crystal structures 44
4. NMR spectra 47
5. References 63

1. General experimental section

All reagents and solvents were purchased from Sigma-Aldrich or Fluorochem and used without further purification unless otherwise specified. Compounds **4**[S1] was synthesised following literature procedure. Dry solvents were obtained by passing through an activated alumina column on a Phoenix SDS solvent drying system (JC Meyer Solvent Systems, CA, USA). NMR spectra were recorded on a BrukerAvance III equipped with a cryoprobe (5mm CPDCH ^{13}C-$^{1}H/D$) instrument with an Oxford AS600 magnet. Chemical shifts are reported in parts per million (ppm) from high to low frequency and referenced to the residual solvent resonance. Coupling constants (*J*) are reported in Hertz (Hz). Standard abbreviations indicating multiplicity were used as follows: s = singlet, d = doublet, t = triplet, q = quartet, quin = quintet, sep = septet, m = multiplet, br = broad. ^{1}H assignments were made using 2D NMR methods (COSY, HSQC, HMBC). Low resolution ESI mass spectrometry was performed with a Thermo Scientific LCQ Fleet or an Advion Expression CMS L single quadrupole MS detector. High resolution ESI (electrospray ionisation) and MALDI-TOF (matrix-assisted laser desorption/ionization time-of-flight) mass spectrometry were carried out by the mass spectrometry services at the University of Manchester. Microwave reactions were performed using a Biotage® Initiator microwave synthesis system. Flash column chromatography was carried out using Silica 60 Å (particle size 40-63 μm, Sigma Aldrich, UK) as the stationary phase. Analytical thin layer chromatography (TLC) was performed on precoated silica gel plates (0.25 mm thick, 60 g F254, Merck, Germany) and visualised using both short and long waved ultraviolet light. Gel permeation chromatography (GPC) was performed using a JAI LC-9160 II NEXT Recycling Preparative HPLC system with 2 × JAIGEL-2.5HR columns (chloroform stabilised with ethanol + 0.5% triethylamine as eluent) with a UV-Vis 4ch NEXT detector.

Abbreviations: DMF *N,N*-dimethylformamide; DMSO dimethylsulfoxide; EDTA ethylenediaminetetraacetate; H.G. II Hoveyda-Grubbs 2nd generation catalyst; PTFE Teflon.

2. Synthesis and experimental

2.1. Synthesis of ligand 1

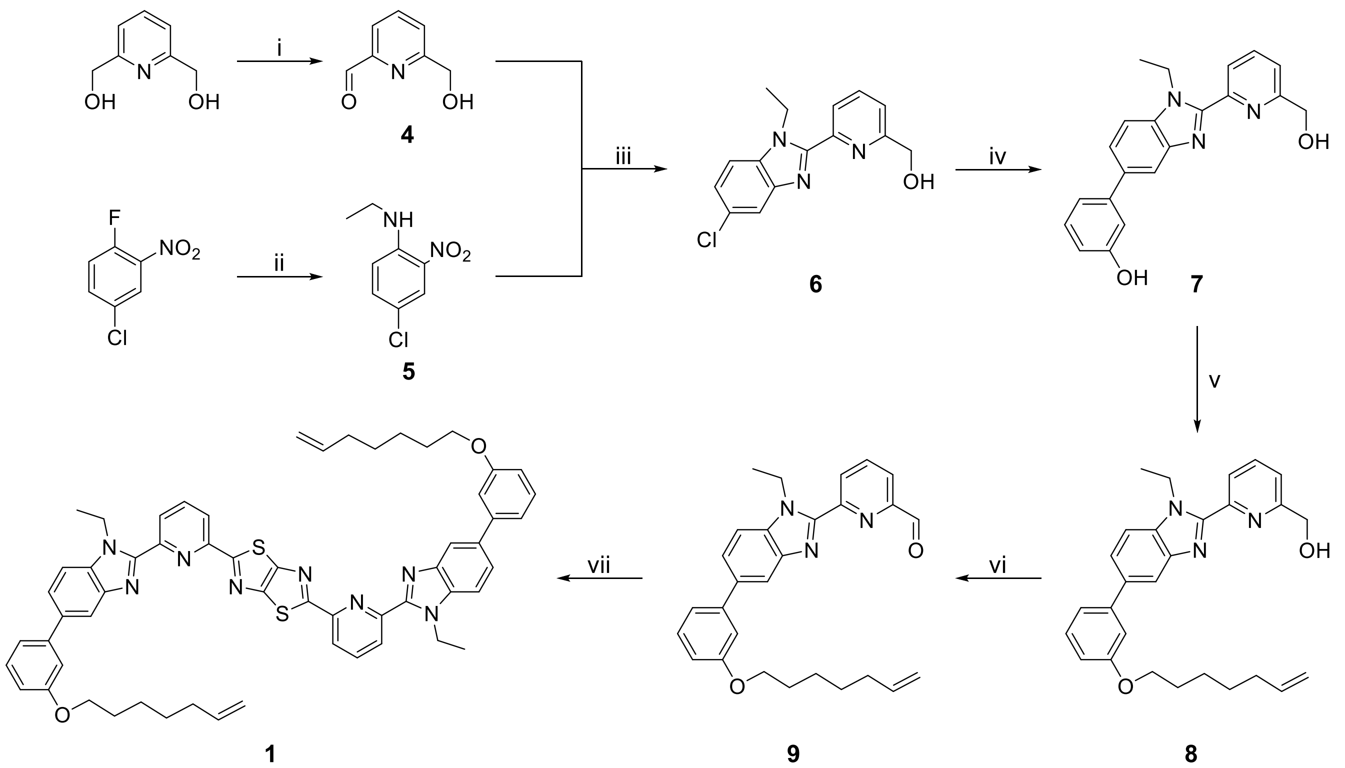

Scheme S1. i) MnO_2, $CHCl_3$, 62°C, 16 h; ii) $EtNH_2{\cdot}HCl$, K_2CO_3, DMSO, 90°C, 2 h; iii) $Na_2S_2O_4$, EtOH/H_2O, N_2, 80°C, 16 h; iv) potassium 3-hydroxyphenyltrifluoroborate, $Pd(OAc)_2$, SPhos, K_2CO_3, EtOH, N_2, 80°C, 24 h; v) 7-bromo-1-heptene, K_2CO_3, DMF, N_2, 50°C, 22 h; vi) MnO_2, $CHCl_3$, 62°C, 17 h; vii) dithiooxamide, 130°C, DMF, 24 h.

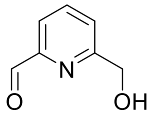

4

6-(hydroxymethyl)picolinaldehyde was synthesized following literature procedure.[S1]

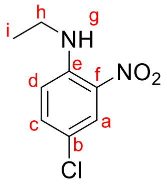

5

A solution of 5-chloro-2-fluoronitrobenzene (5.0 g, 28.5 mmol), ethylamine hydrochloride (23.2 g, 285 mmol) and potassium carbonate (43.2 g, 313 mmol) in DMSO (170 mL) was heated at 90°C for 2 hours. Upon cooling, water (200 mL) and diethyl ether (200 mL) were added, giving two phases. The organic layer was washed with brine (5 x 50 mL). The organic layer was dried over $MgSO_4$ and the solvent removed under reduced pressure. The desired product **5** was obtained as an orange solid (5.54 g, 97%), and was deemed sufficiently pure to use in the following step without further purification. ^{1}H NMR (600 MHz, Chloroform-*d*) δ 8.17 (d, *J* = 2.6 Hz, 1H, H^{a}), 7.95 (br. s, 1H, H^{g}), 7.38 (dd, *J* = 9.2, 2.3 Hz, 1H, H^{c}), 6.81 (d, *J* = 9.2 Hz, 1H, H^{d}), 3.34 (qd, *J* = 7.2, 5.3 Hz, 2H, H^{h}), 1.37 (t, *J* = 7.2 Hz, 3H, H^{i}). ^{13}C NMR (151 MHz, Chloroform-*d*) δ 144.21 (C^{e}), 136.50 (C^{c}), 131.64 (C^{f}), 126.08 (C^{a}), 120.01 (C^{b}), 115.31 (C^{d}), 38.01 (C^{h}), 14.45 (C^{i}). LR ESI-MS: *m/z* = 223.0 [M+Na]$^{+}$ (calcd. for $C_8H_9ClN_2O_2Na$, 223.02).

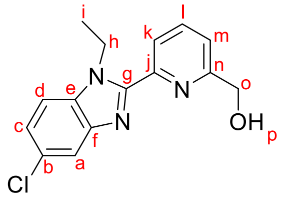

6

6-(Hydroxymethyl)picolinaldehyde (4.16 g, 30.4 mmol) and **5** (5.54 g, 27.6 mmol) were added into a mixture of ethanol (220 mL) and water (80 mL). The solution was degassed by bubbling argon for 1 hour and subsequently heated to 80°C until complete dissolution of the solids. Sodium dithionite (17.0 g, 82.8 mmol) was added in one portion under positive pressure of nitrogen, yielding a bright yellow suspension. The mixture was stirred under nitrogen at 80°C for 16 hours. After cooling to room temperature, most of the solvent was removed under reduced pressure. The residue was partitioned between CH_2Cl_2 (150 mL) and saturated aqueous Na_2CO_3 (100 mL), and after separation the aqueous layer was extracted with more CH_2Cl_2 (2 x 50 mL). The combined organic layers were washed with brine (50 mL) before drying over $MgSO_4$ and the solvent was removed under reduced pressure. Recrystallization from toluene yielded **6** as a yellow solid (4.15 g, 52%). ^{1}H NMR (600 MHz, Chloroform-*d*) δ 8.27 (d, *J* = 7.8 Hz, 1H, H^{k}), 7.87 (t, *J* = 7.8 Hz, 1H, H^{l}), 7.81 (d, *J* = 1.9 Hz, 1H, H^{a}), 7.39 – 7.36 (m, 2H, H^{d}, H^{m}), 7.31 (dd, *J* = 8.6, 1.9 Hz, 1H, H^{c}), 4.88 (s, 2H, H^{o}), 4.76 (q, *J* = 7.2 Hz, 2H, H^{h}), 3.38 (br. s, 1H, H^{p}), 1.54 (t, *J* = 7.2 Hz, 3H, H^{i}). ^{13}C NMR (151 MHz, Chloroform-*d*) δ 158.83 (C^{n}), 150.65 (C^{g}), 149.19 (C^{j}), 143.56 (C^{f}), 137.92 (C^{l}), 134.91 (C^{e}), 128.41 (C^{b}), 124.07 (C^{c}), 123.83 (C^{k}), 120.97 (C^{m}), 120.01 (C^{a}), 110.89 (C^{d}), 64.60 (C^{o}), 40.95 (C^{h}), 15.55 (C^{i}). LR ESI-MS: *m/z* = 288.2 [M+H]$^{+}$ (calcd. for $C_{15}H_{15}ClN_3O$, 288.09).

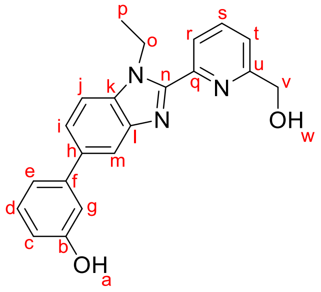

7

6 (2.00 g, 6.95 mmol), potassium 3-hydroxyphenyltrifluoroborate (2.08 g, 10.4 mmol), palladium(II) acetate (78 mg, 0.35 mmol), SPhos (0.28 g, 0.70 mmol) and potassium carbonate (2.88 g, 20.8 mmol) were dissolved in anhydrous and degassed ethanol (35 mL), and the mixture was heated to reflux for 24 hours. After cooling to room temperature, the solvent was removed under reduced pressure. The residue was dissolved in a 3:1 mixture of ethyl acetate-methanol, filtered through silica and eluted with additional eluent. Purification by flash column chromatography (SiO_2, CH_2Cl_2:AcOEt:MeOH, 8:2:0.2 to 8:2:0.5) yielded **7** as a yellow solid (1.33 g, 55%). ^{1}H NMR (600 MHz, Methanol-*d*$_4$) δ 8.11 (d, *J* = 7.7 Hz, 1H, H^r), 8.01 (t, *J* = 7.8 Hz, 1H, H^s), 7.89 (d, *J* = 1.0 Hz, 1H, H^m), 7.67 – 7.64 (m, 2H, H^j + H^t), 7.62 (dd, *J* = 8.5, 1.6 Hz, 1H, H^i), 7.27 (t, *J* = 7.8 Hz, 1H, H^d), 7.14 (d, *J* = 7.7 Hz, 1H, H^e), 7.12 – 7.10 (m, 1H, H^g), 6.78 (ddd, *J* = 8.0, 2.3, 0.7 Hz, 1H, H^c), 4.86 – 4.81 (m, 4H, H^o + H^v), 1.51 (t, *J* = 7.1 Hz, 3H, H^p). ^{13}C NMR (151 MHz, Methanol-*d*$_4$) δ 162.57 (C^u), 158.96 (C^b), 151.74 (C^o), 150.02 (C^q), 144.23 (C^f), 143.61 (C^l), 139.15 (C^s), 138.14 (C^h), 136.61 (C^k), 130.88 (C^d), 124.50 (C^i), 123.93 (C^r), 122.25 (C^t), 119.54 (C^e), 118.05 (C^m), 115.04 (C^c or C^g), 114.98 (C^c or C^g), 111.80 (C^j), 66.01 (C^v), 41.76 (C^n), 15.70 (C^p). LR ESI-MS: *m/z* = 346.2 $[M+H]^+$ (calcd. for $C_{21}H_{20}N_3O_2$, 346.16).

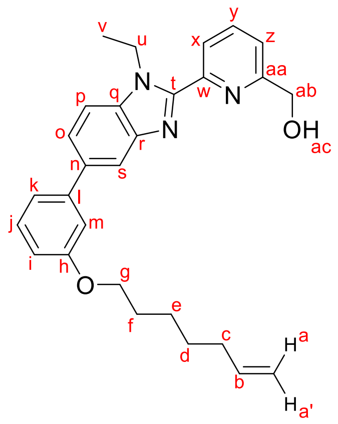

8

A solution of **7** (1.33 g, 3.85 mmol), 7-bromo-1-heptene (1.02 g, 5.77 mmol) and potassium carbonate (1.06 g, 7.70 mmol) in DMF (29 mL) was heated to 50°C for 22 hours under an atmosphere of nitrogen. Upon cooling, the solvent was removed, and the residue partitioned between dichloromethane (60 mL) and 5% aqueous lithium chloride (100 mL). The organic layer was sequentially washed with 5% aqueous lithium chloride (5 x 50 mL) and brine (50 mL). The organic layers were dried over $MgSO_4$ and the solvent removed under reduced pressure. Flash column chromatography (SiO_2, CH_2Cl_2:MeOH, 100:0 to 95:5) yielded **8** as a brown oil (1.08 g, 63%). ^{1}H NMR (600 MHz, Chloroform-*d*) δ 8.34 (d, *J* = 7.8 Hz, 1H, H^x), 8.05 (d, *J* = 1.0 Hz, 1H, H^s), 7.88 (t, *J* = 7.8 Hz, 1H, H^y), 7.61 (dd, *J* = 8.4, 1.6 Hz, 1H, H^o), 7.51 (d, *J* = 8.4 Hz, 1H, H^p), 7.39 – 7.34 (m, 2H, H^z + H^j), 7.27 – 7.24 (m, 1H, H^k), 7.22 – 7.20 (m, 1H, H^m), 6.89 (dd, *J* = 8.1, 2.1 Hz, 1H, H^i), 5.83 (ddt, *J* = 16.9, 10.2, 6.7 Hz, 1H, H^b), 5.02 (dq, *J* = 17.1, 1.6 Hz, 1H, H^a), 4.97 – 4.94 (m, 1H, $H^{a'}$), 4.90 (s, 2H, H^{ab}), 4.82 (q, *J* = 7.2 Hz, 2H, H^u), 4.04 (t, *J* = 6.6 Hz, 2H, H^g), 3.43 (br. s, 1H, H^{ac}), 2.10 (q, *J* = 6.8 Hz, 2H, H^c), 1.84 (p, *J* = 6.6 Hz, 2H, H^f), 1.59 (t, *J* = 7.2 Hz, 3H, H^v), 1.56 – 1.45 (m, 4H, H^d + H^e). ^{13}C NMR (151 MHz, Chloroform-*d*) δ 159.61 (C^h), 158.63 (C^{aa}), 150.16 (C^t), 149.53 (C^w), 143.35 (2C, C^l + C^r), 139.00 (C^c), 137.86 (C^y), 136.49 (C^n), 135.94 (C^q), 129.88 (C^j), 123.74 (C^x), 123.51 (C^o), 120.71 (C^z), 119.87 (C^k), 118.68 (C^s), 114.60 (C^a), 113.76 (C^m), 113.13 (C^i), 110.20 (C^p), 68.08 (C^g), 64.57 (C^{ab}), 40.92 (C^u), 33.87 (C^d), 29.33 (C^f), 28.81 (C^d), 25.74 (C^e), 15.64 (C^v). LR ESI-MS: *m/z* = 442.4 $[M+H]^+$ (calcd. for $C_{28}H_{31}N_3O_2$, 442.25).

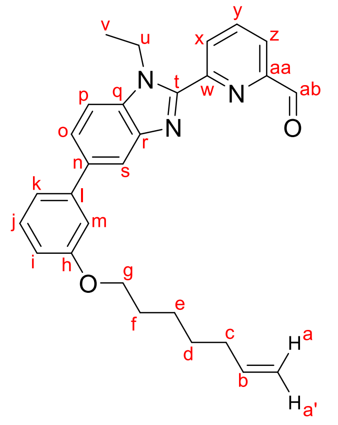

9

To a solution of **8** (1.08 g, 2.45 mmol) in chloroform (60 mL) was added MnO_2 (2.37 g, 24.5 mmol) and the suspension was stirred under reflux for 17 hours. Upon cooling, the mixture was filtered over Celite® and eluted with a 9:1 $CHCl_3$/MeOH mixture. The solvent

from the filtrate was removed under reduced pressure. The desired product **9** was obtained as a yellow oil (1.03 g, 96%), and was deemed sufficiently pure to use in the following step without further purification. ^{1}H NMR (600 MHz, Chloroform-*d*) δ 10.16 (s, 1H, H^{ab}), 8.72 (d, *J* = 7.3 Hz, 1H, H^{x}), 8.09 – 8.01 (m, 3H, H^{s} + H^{y} + H^{z}), 7.64 (d, *J* = 8.1 Hz, 1H, H^{o}), 7.55 (d, *J* = 8.3 Hz, 1H, H^{p}), 7.37 (t, *J* = 7.9 Hz, 1H, H^{j}), 7.28 – 7.24 (m, 1H, H^{k}), 7.22 (s, 1H, H^{m}), 6.90 (dd, *J* = 8.1, 2.0 Hz, 1H, H^{i}), 5.83 (ddt, *J* = 16.9, 10.2, 6.7 Hz, 1H, H^{b}), 5.05 – 5.00 (m, 1H, H^{a}), 5.00 – 4.93 (m, 3H, $H^{a'}$ + H^{u}), 4.04 (t, *J* = 6.5 Hz, 2H, H^{g}), 2.11 (q, *J* = 6.8 Hz, 2H, H^{c}), 1.84 (p, *J* = 6.6 Hz, 2H, H^{f}), 1.63 (t, *J* = 7.0 Hz, 3H, H^{v}), 1.54 – 1.46 (m, 4H, H^{d} + H^{e}). ^{13}C NMR (151 MHz, Chloroform-*d*) δ 192.88 (C^{ab}), 159.63 (C^{h}), 152.10 (C^{aa}), 151.25 (C^{w}), 149.08 (C^{t}), 143.30 (C^{r}), 143.24 (C^{l}), 138.99 (C^{b}), 138.20 (C^{y}), 136.69 (C^{n}), 136.16 (C^{q}), 129.91 (C^{j}), 128.88 (C^{x}), 123.90 (C^{o}), 121.67 (C^{z}), 119.86 (C^{k}), 118.77 (C^{s}), 114.61 (C^{a}), 113.78 (C^{m}), 113.17 (C^{i}), 110.33 (C^{p}), 68.09 (C^{g}), 41.21 (C^{u}), 33.86 (C^{c}), 29.32 (C^{f}), 28.81 (C^{d}), 25.74 (C^{e}), 15.69 (C^{v}). LR ESI-MS: *m/z* = 472.4 $[M+MeOH+H]^{+}$ (calcd. for $C_{29}H_{34}N_{3}O_{3}$, 472.26).

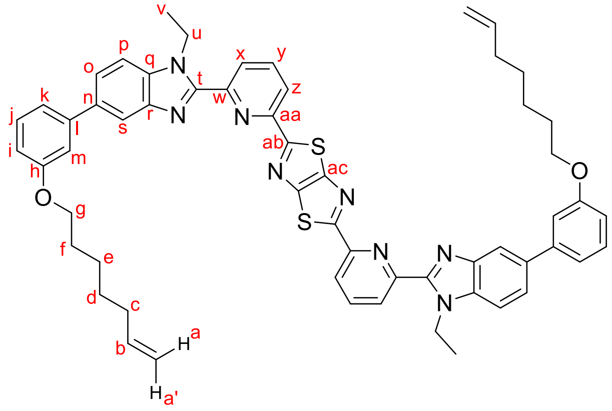

1

A solution of **9** (1.03 g, 2.34 mmol) and dithiooxamide (0.14 g, 1.17 mmol) in DMF (26 mL) was heated at 130°C for 24 hours. After cooling, precipitation *via* addition of methanol (250 mL) followed by vacuum filtration and washing with excess methanol afforded **1** (843 mg, 75%) as a yellow powder, which required no further purification. ^{1}H NMR (600 MHz, Chloroform-*d*) δ 8.61 (d, *J* = 7.7 Hz, 2H, H^{x}), 8.31 (d, *J* = 7.7 Hz, 2H, H^{z}), 8.08 (s, 2H, H^{s}), 8.03 (t, *J* = 7.8 Hz, 2H, H^{y}), 7.65 (dd, *J* = 8.3, 1.3 Hz, 2H, H^{o}), 7.57 (d, *J* = 8.5 Hz, 2H, H^{p}), 7.37 (t, *J* = 7.8 Hz, 2H, H^{j}), 7.27 (s, 1H, H^{k}), 7.23 (s, 2H, H^{m}), 6.90 (dd, *J* = 8.1, 2.1 Hz, 2H, H^{i}), 5.84 (ddt, *J* = 13.3, 10.2, 6.7 Hz, 2H, H^{b}), 5.08 – 4.99 (m, 6H, H^{a} + H^{u}), 4.96 (d, *J* = 10.2 Hz, 2H, $H^{a'}$), 4.05 (t, *J* = 6.5 Hz, 4H, H^{g}), 2.14 – 2.08 (m, 4H, H^{c}), 1.88 – 1.81 (m, 4H, H^{f}), 1.75 (t, *J* = 7.1 Hz, 6H, H^{v}), 1.54 – 1.47 (m, 8H, H^{d} + H^{e}). ^{13}C NMR (151 MHz, Chloroform-*d*) δ 170.77 (C^{ab}), 159.62 (C^{h}), 153.41 (C^{ac}), 150.57 (C^{aa} or C^{w}), 150.41 (C^{aa} or C^{w}), 148.89 (C^{t}), 143.21 (2C, C^{l} + C^{r}), 139.00 (C^{b}), 138.24 (C^{y}), 136.61 (C^{n}), 136.13 (C^{q}), 129.90 (C^{j}), 126.28 (C^{x}), 123.80 (C^{o}), 120.01 (C^{z}), 119.83 (C^{k}), 118.68 (C^{s}), 114.61 (C^{a}), 113.74 (C^{m}), 113.13 (C^{i}), 110.32 (C^{p}), 68.08 (C^{g}), 41.23 (C^{u}), 33.87 (C^{c}), 29.33 (C^{f}), 28.82 (C^{d}), 25.74 (C^{e}), 16.02 (C^{v}). MALDI-TOF MS: *m/z* = 983.9 $[M+Na]^{+}$ (calcd. for $C_{58}H_{56}N_{8}O_{2}S_{2}Na$, 983.39).

2.2. Synthesis of molecular grid [Fe$_4$**1**$_4$](BF$_4$)$_8$, 6_3^2 link 2 and granny knot 3

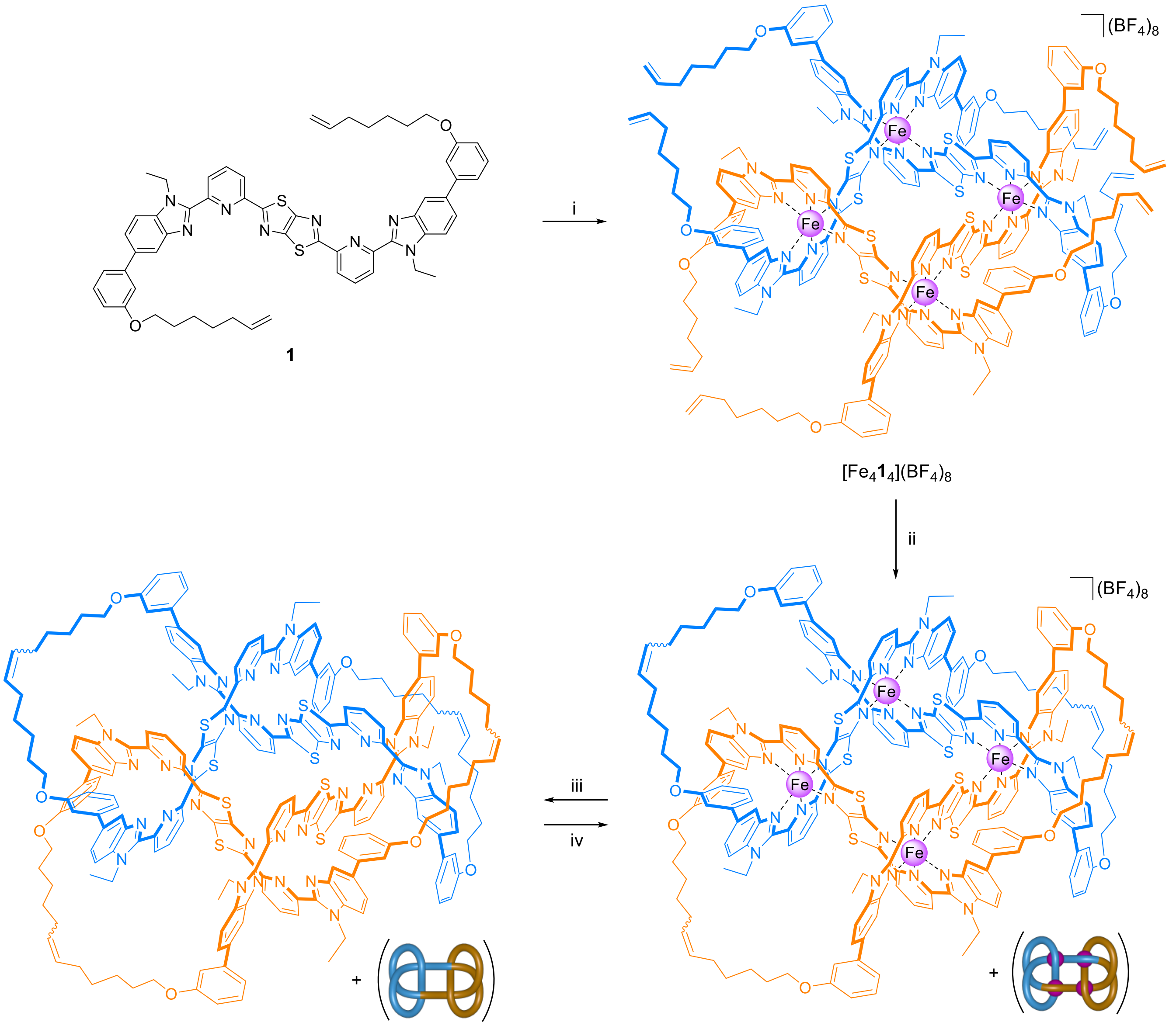

Scheme S2. i) Fe(BF$_4$)$_2$·6H$_2$O, CH$_2$Cl$_2$/MeCN, r.t., 15 min; ii) H.G. II, CH$_2$Cl$_2$/MeNO$_2$, N$_2$, MW, 110°C, 3h; iii) Na$_4$EDTA sat. aq. sol., r.t.; iv) Fe(BF$_4$)$_2$·6H$_2$O, CHCl$_3$/MeCN, 24h, 50°C.

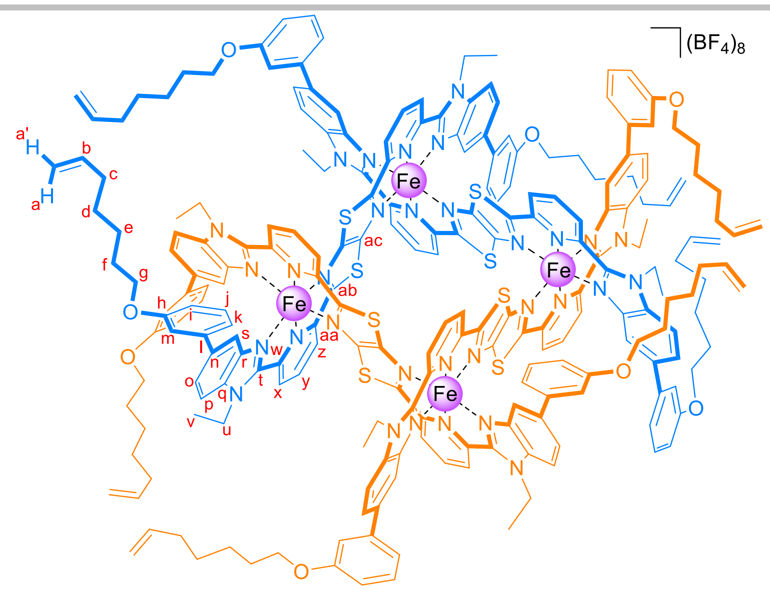

[Fe_4**1**$_4$]$(BF_4)_8$

A solution of iron(II) tetrafluoroborate hexahydrate (183 mg, 0.541 mmol) in acetonitrile (12 mL) was added to a solution of **1** (400 mg, 0.416 mmol) in CH_2Cl_2 (37 mL) at room temperature. Within a minute the brownish yellow solution had turned dark green. The mixture was stirred for 15 more minutes, after which the solvents were removed under reduced pressure. The residue was redissolved in acetonitrile, precipitated by addition of distilled water and filtered over Celite®. The green precipitate was washed with more distilled water (200 mL) and taken into acetonitrile. Removal of the solvent under reduced pressure afforded the desired complex [Fe_4**1**$_4$]$(BF_4)_8$ (401 mg, 81%) as a dark green solid. ^{1}H NMR (600 MHz, Acetonitrile-d_3) δ 9.72 (br. s, 8H, H^x or H^z), 9.52 (br. s, 8H, H^x or H^z), 9.11 (t, J = 8.2 Hz, 8H, H^y), 7.50 (d, J = 7.9 Hz, 8H, H^o), 7.40 – 7.37 (m, 8H, H^j), 7.35 (d, J = 7.9 Hz, 8H, H^p), 6.94 (dd, J = 8.4, 2.1 Hz, 8H, H^k), 6.61 (s, 8H, H^m), 6.50 (d, J = 7.0 Hz, 8H, H^i), 5.92 (ddt, J = 17.0, 10.2, 6.7 Hz, 8H, H^b), 5.15 (br. s, 8H, H^s), 5.08 (dq, J = 17.2, 1.5 Hz, 8H, H^a), 5.02 – 4.98 (m, 8H, $H^{a'}$), 4.90 – 4.77 (m, 16H, H^u), 4.01 (t, J = 6.6 Hz, 16H, H^g), 2.12 (s, 16H, H^c), 1.88 – 1.82 (m, 16H, H^f), 1.56 – 1.51 (m, 32H, H^d + H^e), 1.24 (t, J = 7.3 Hz, 24H, H^v). ^{13}C NMR (151 MHz, Acetonitrile-d_3) δ 160.54 (C^h), 150.39 (2C,C^{aa} + C^x), 143.31 (C^y), 141.31 (C^l or C^n), 139.99 (C^c), 138.93 (C^l or C^n), 131.13 (C^j), 126.53 (C^p), 119.94 (C^i), 115.02 (C^a), 114.81 (C^o), 114.42 (C^k), 114.22 (C^m), 113.70 (C^s), 68.89 (C^g), 42.81 (C^u), 34.38 (C^c), 29.80 (C^f), 29.39 (C^d), 26.27 (C^e), 16.29 (C^v). Carbons q, r, t, ab, ab, x, z could not be observed. LR ESI-MS: m/z = 865.5 $[M{-}5BF_4]^{5+}$ calculated 865.3; 706.8 $[M{-}6BF_4]^{6+}$ calculated 706.6; 593.5 $[M{-}7BF_4]^{7+}$ calculated 593.2.

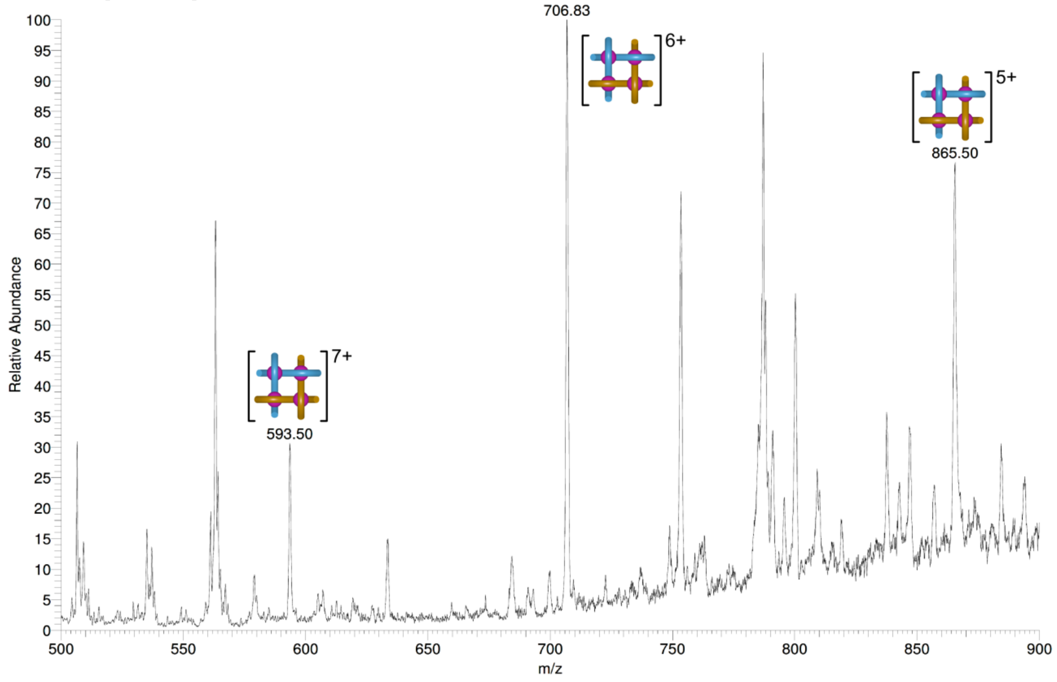

Figure S1. Low-resolution ESI-MS of [Fe_4**1**$_4$]$(BF_4)_8$ (all peaks observed as the [Fe_4**1**$_4$+(8–n)BF_4]$^{n+}$ adducts). Calculated peaks (m/z): 865.3 $[M{-}5BF_4]^{5+}$; 706.6 $[M{-}6BF_4]^{6+}$; 593.2 $[M{-}7BF_4]^{7+}$.

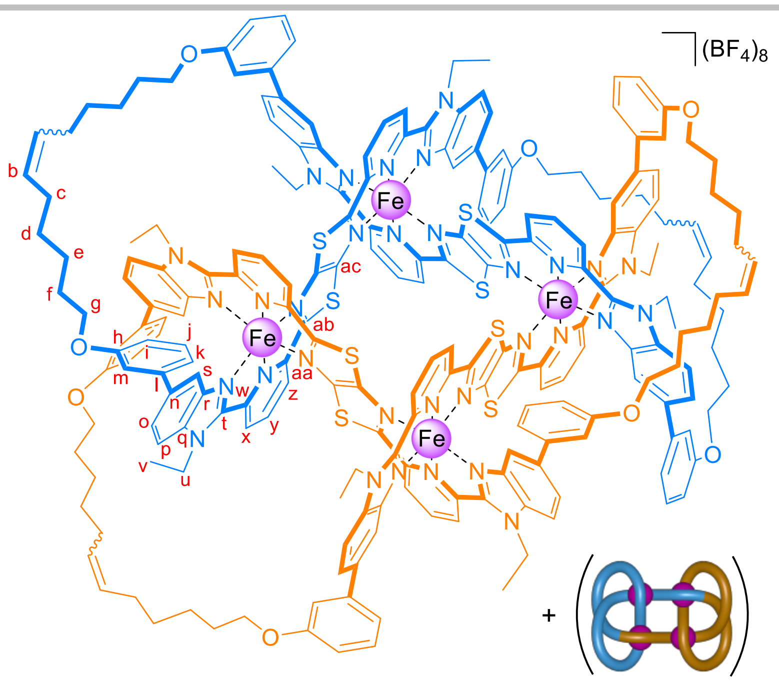

[Fe_4**2/3**]$(BF_4)_8$

A solution of Hoveyda-Grubbs 2nd generation catalyst (5.3 mg, 8.4 μmol, 0.8 equiv.) in anhydrous and degassed dichloromethane (7.5 mL) was added to a solution of [Fe_4**1**$_4$]$(BF_4)_8$ (50.0 mg, 10.5 μmol) in anhydrous and degassed nitromethane (2.5 mL). The mixture was heated under microwave irradiation in a sealed vial at 110°C for 3 hours. Upon cooling, ethyl vinyl ether (4 mL) was added to the reaction. The mixture was stirred for further 30 minutes, before removing the solvent under reduced pressure. The resulting dark green solid showed full conversion of the starting material by ^{1}H NMR (Figure S2). The crude material was taken forward to the next step without further purification. LR ESI-MS: *m/z* = 1462.8 $[M–3BF_4]^{3+}$ calculated 1462.7; 1075.5 $[M–4BF_4]^{4+}$ calculated 1075.3; 843.1 $[M–5BF_4]^{5+}$ calculated 842.8; 688.3 $[M–6BF_4]^{6+}$ calculated 687.9; 577.5 $[M–7BF_4]^{7+}$ calculated 577.2.

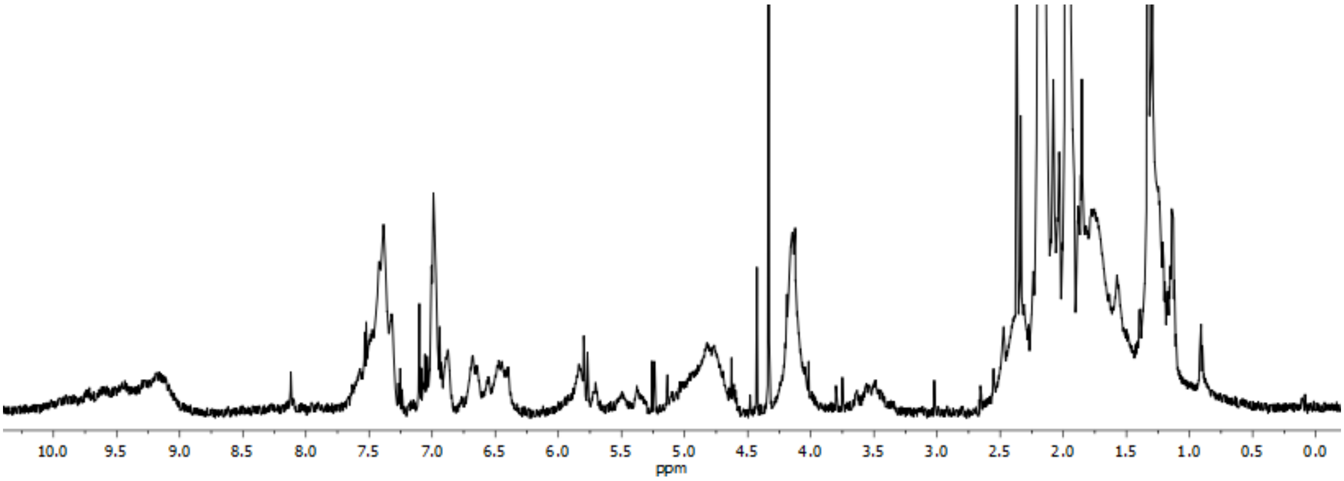

Figure S2. ^{1}H NMR (600 MHz, CD_3CN) spectrum of [Fe_4**2/3**]$(BF_4)_8$ mixture from the ring closing metathesis (RCM) reaction.

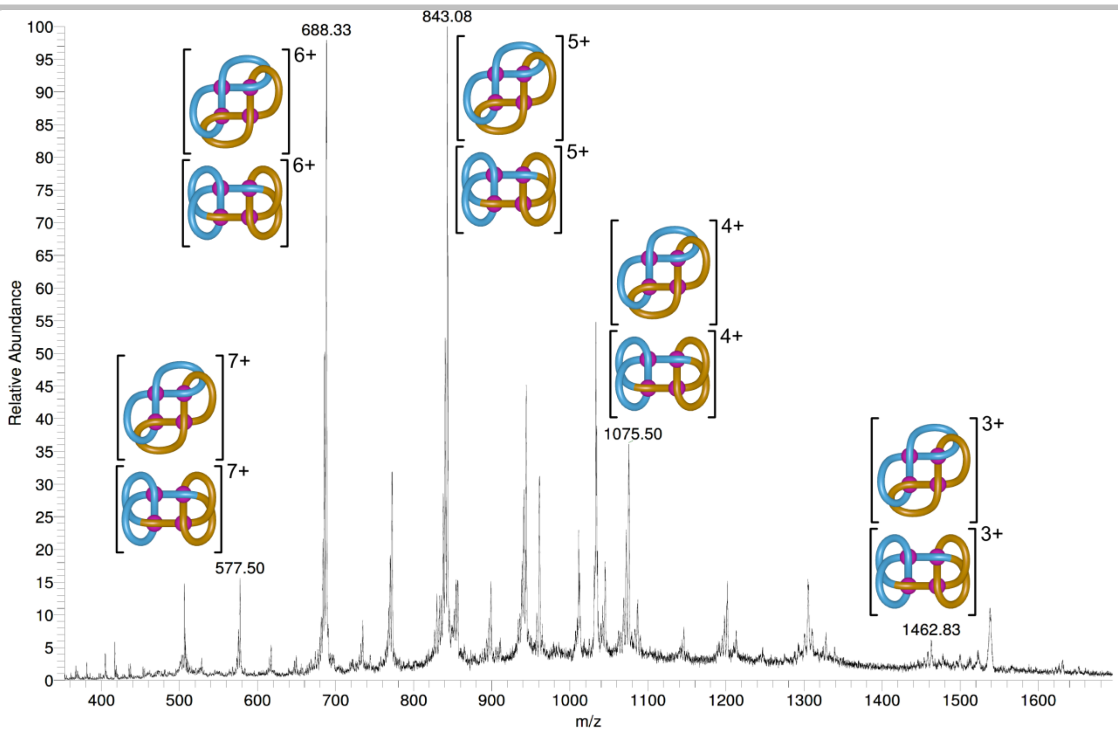

Figure S3. Low-resolution ESI-MS of [Fe_4**2**/**3**](BF_4)$_8$ after ring closing metathesis (all peaks observed as the [Fe_4**2**/**3**+(8–n)BF_4]$^{n+}$ adducts). Calculated peaks (*m/z*): 1462.7 [M–3BF_4]$^{3+}$; 1075.3 [M–4BF_4]$^{4+}$; 842.8 [M–5BF_4]$^{5+}$; 687.9 [M–6BF_4]$^{6+}$; 577.2 [M–7BF_4]$^{7+}$.

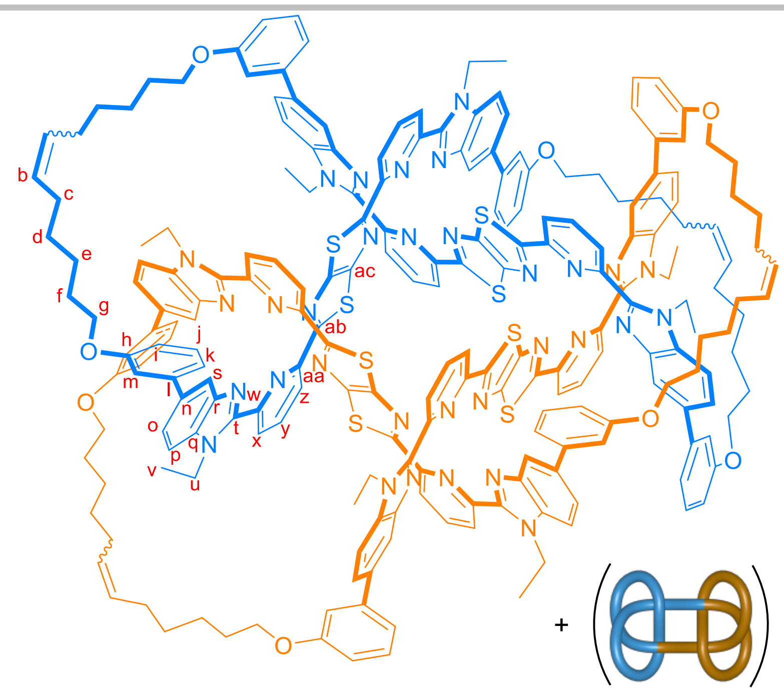

2/**3**

[Fe_4**2**/**3**]$(BF_4)_8$ (306 mg, 66 μmol) was dissolved in acetonitrile (15 mL) and a saturated aqueous solution of Na_4EDTA (60 mL), H_2O (100 mL) and $CHCl_3$ (100 mL) were added. The mixture was vigorously shaken in a extraction funnel, and the phases were separated. The aqueous layer was further extracted with $CHCl_3$ (4 x 30 mL), and the combined organic layers were dried over Na_2SO_4 and the solvent removed under reduced pressure. The crude organic material was purified by recycling GPC, affording **2/3** (Figure S5, green fraction) (55 mg, 18%) as a brownish yellow powder. Fractions containing non-interlocked macrocycle (Figure S5, blue fraction) and non-ring-closed compounds (Figure S5, magenta fraction) were also collected. All fractions were analysed by MALDI-TOF MS in order to identify the isolated species and to assess the purity of the fractions.

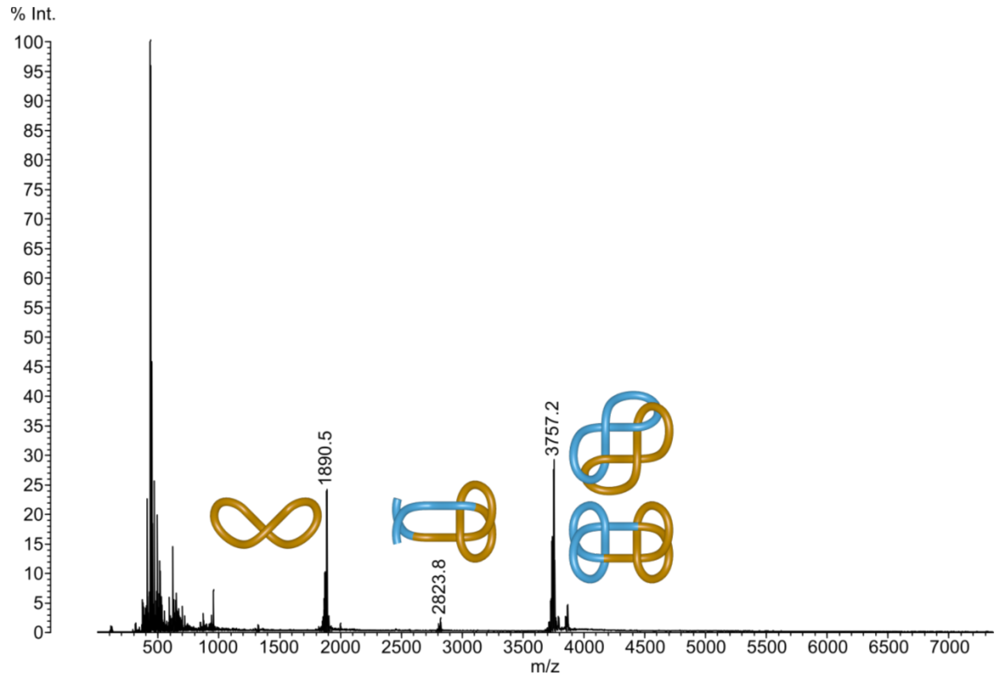

Figure S4. MALDI-TOF MS of the demetallated mixture prior to GPC separation. Calculated peaks (*m/z*): 1887.7 (macrocycle, $[M+Na]^+$), 2826.1 (non-closed fragment, $[M+H]^+$), 3752.4 (link **2**/knot **3**, $[M+Na]^+$).

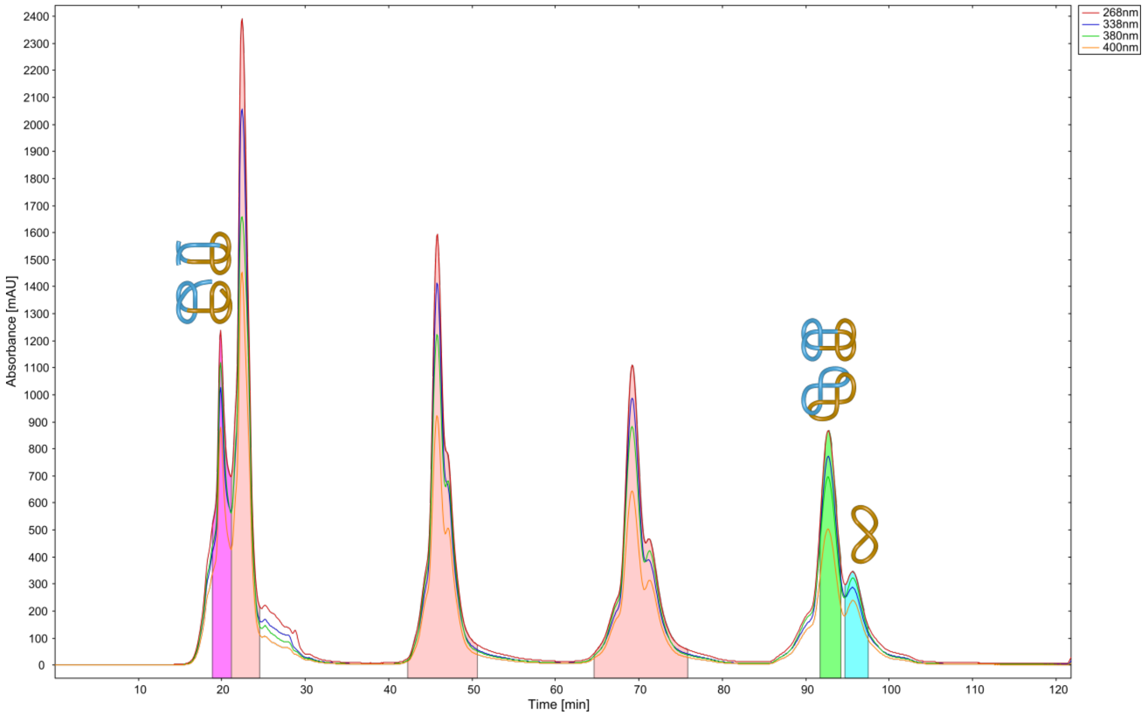

Figure S5. Representative recycling GPC UV-trace for the purification of the link **2**/knot **3** mixture (green fraction). The red regions indicate the intervals of recycling during the elution. Fractions containing non-interlocked macrocycle (blue fraction) and non-ring-closed compounds (magenta fraction) were also collected separately. The identity of all the fractions was determined by MALDI-TOF MS (Figures S6 to S8).

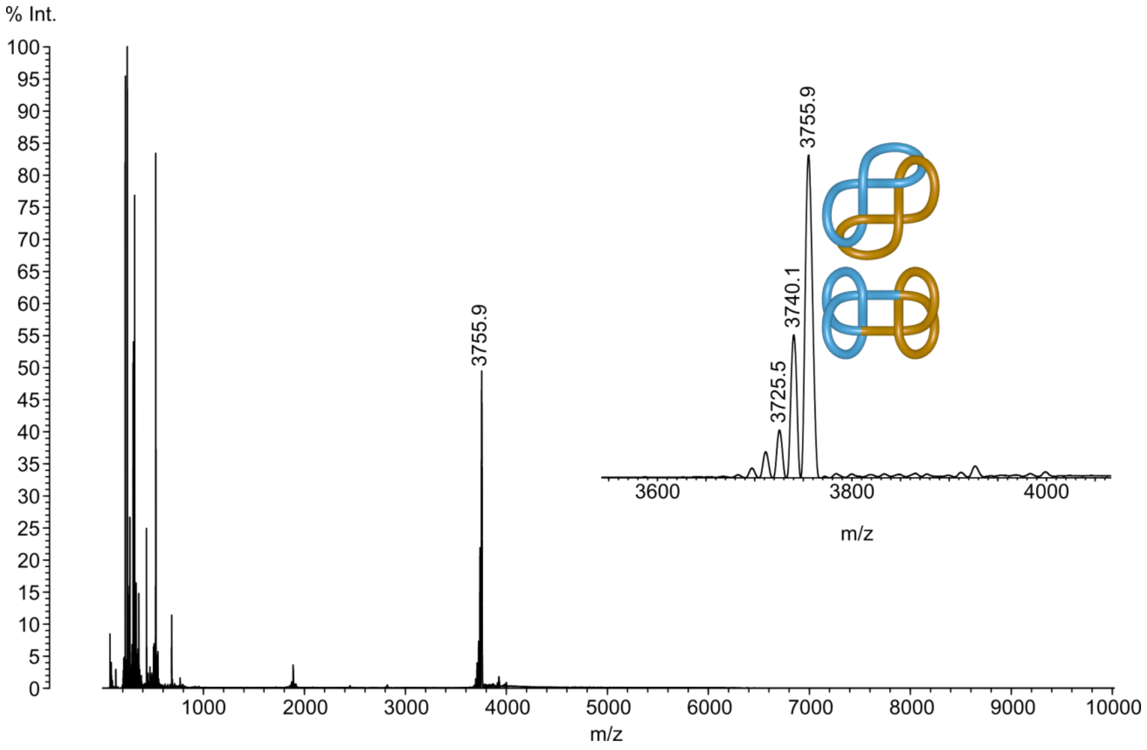

Figure S6. MALDI-TOF MS of pure link **2**/knot **3** mixture (Figure S5, green fraction) after GPC separation (peak observed as the $[M+Na]^+$ adduct). Calculated peak (*m/z*): 3752.4.

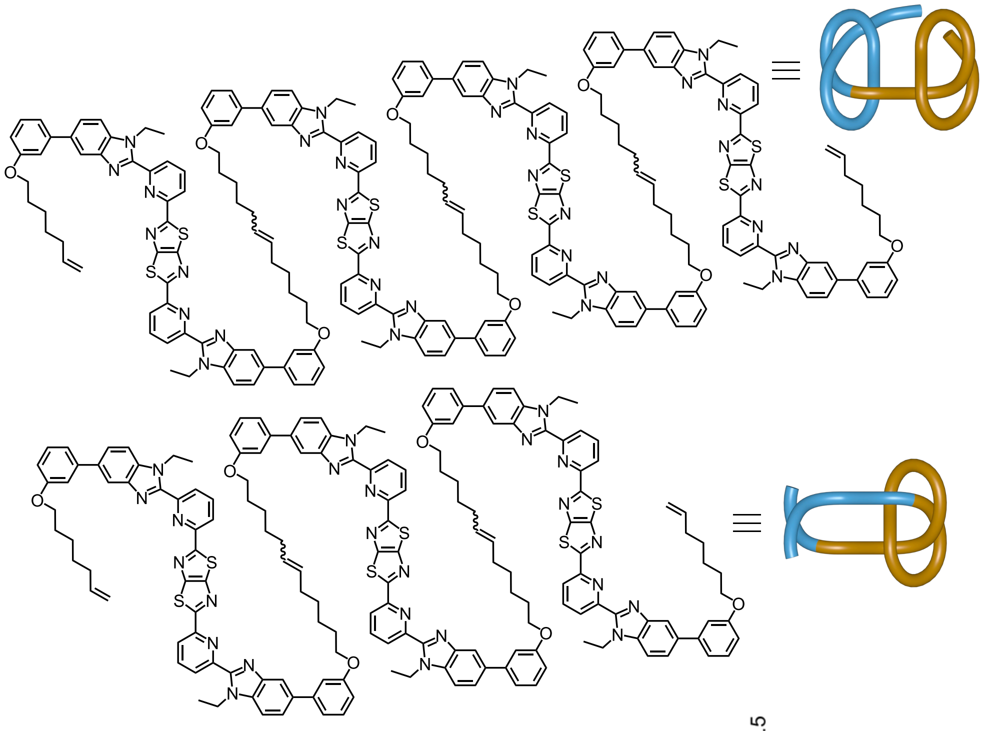

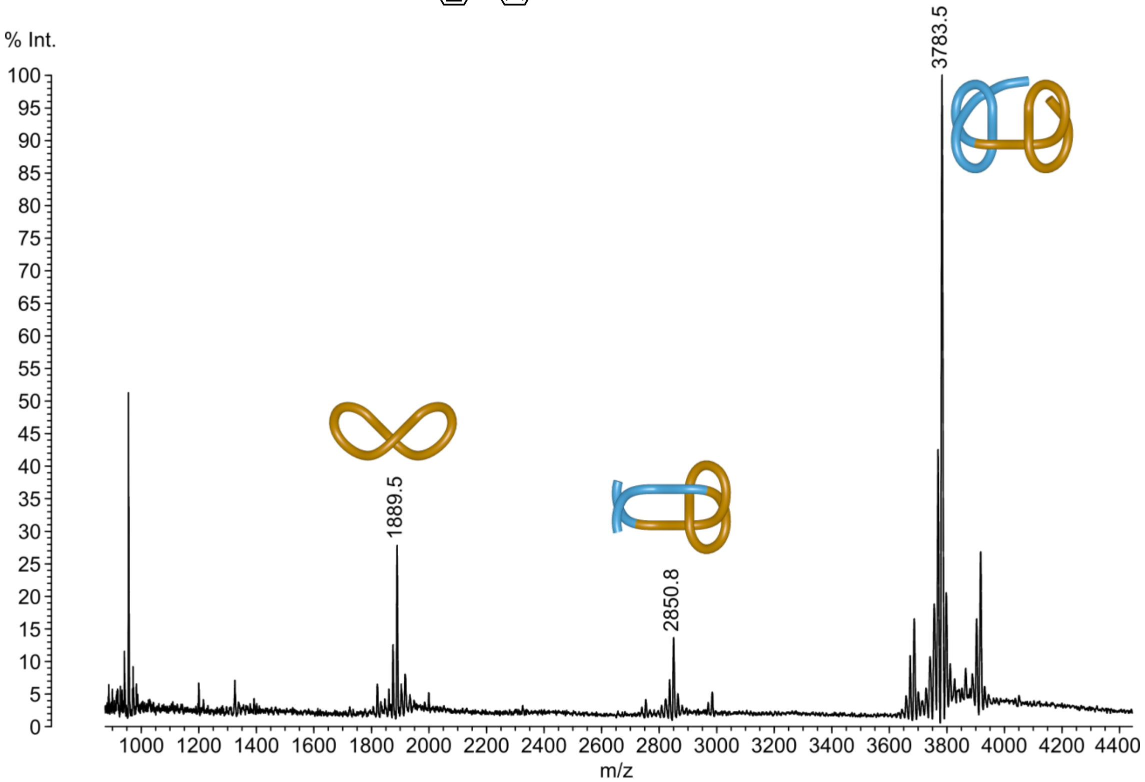

Figure S7. MALDI-TOF MS of a non-ring-closed fraction of compounds (Figure S5, magenta fraction), isolated from GPC separation (all peaks observed as the $[M+Na]^+$ adducts). Calculated peaks (*m/z*): 1887.7 (macrocycle), 2848.1 (non-closed fragment), 3780.5 (non-closed knot).

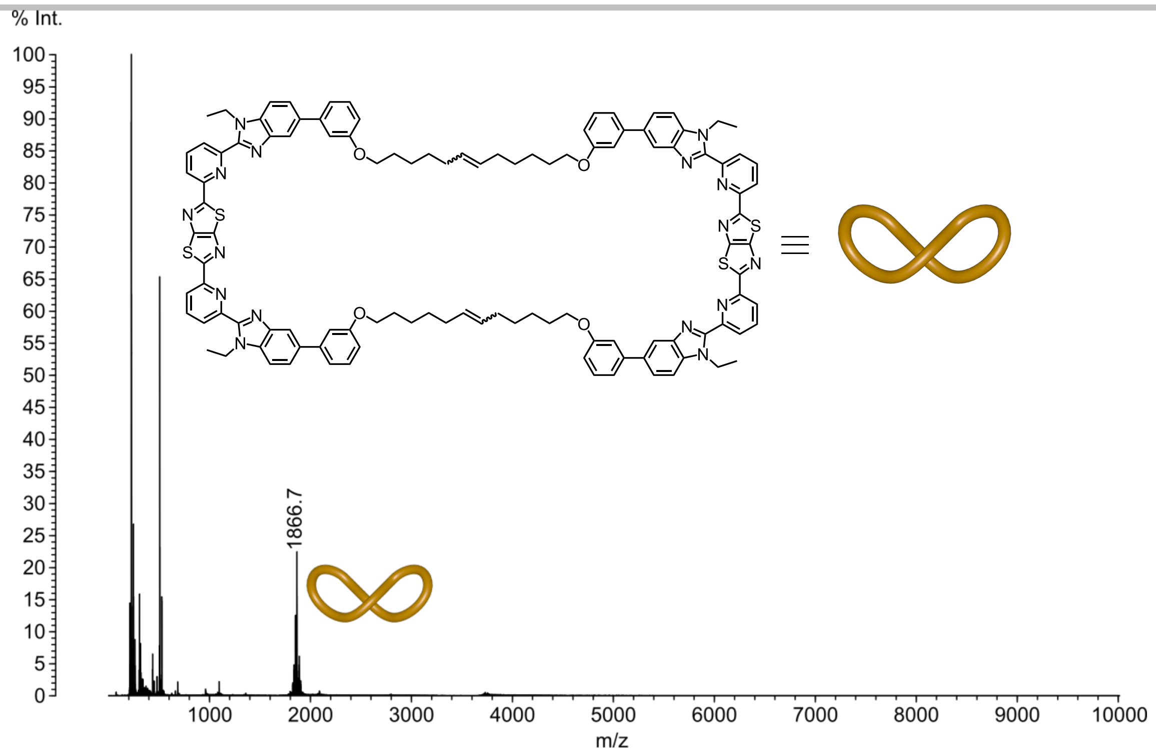

Figure S8. MALDI-TOF MS of non-interlocked macrocycle (Figure S5, blue fraction) isolated from GPC separation (peak observed as the $[M+H]^+$ adduct). Calculated peak (*m/z*): 1865.7.

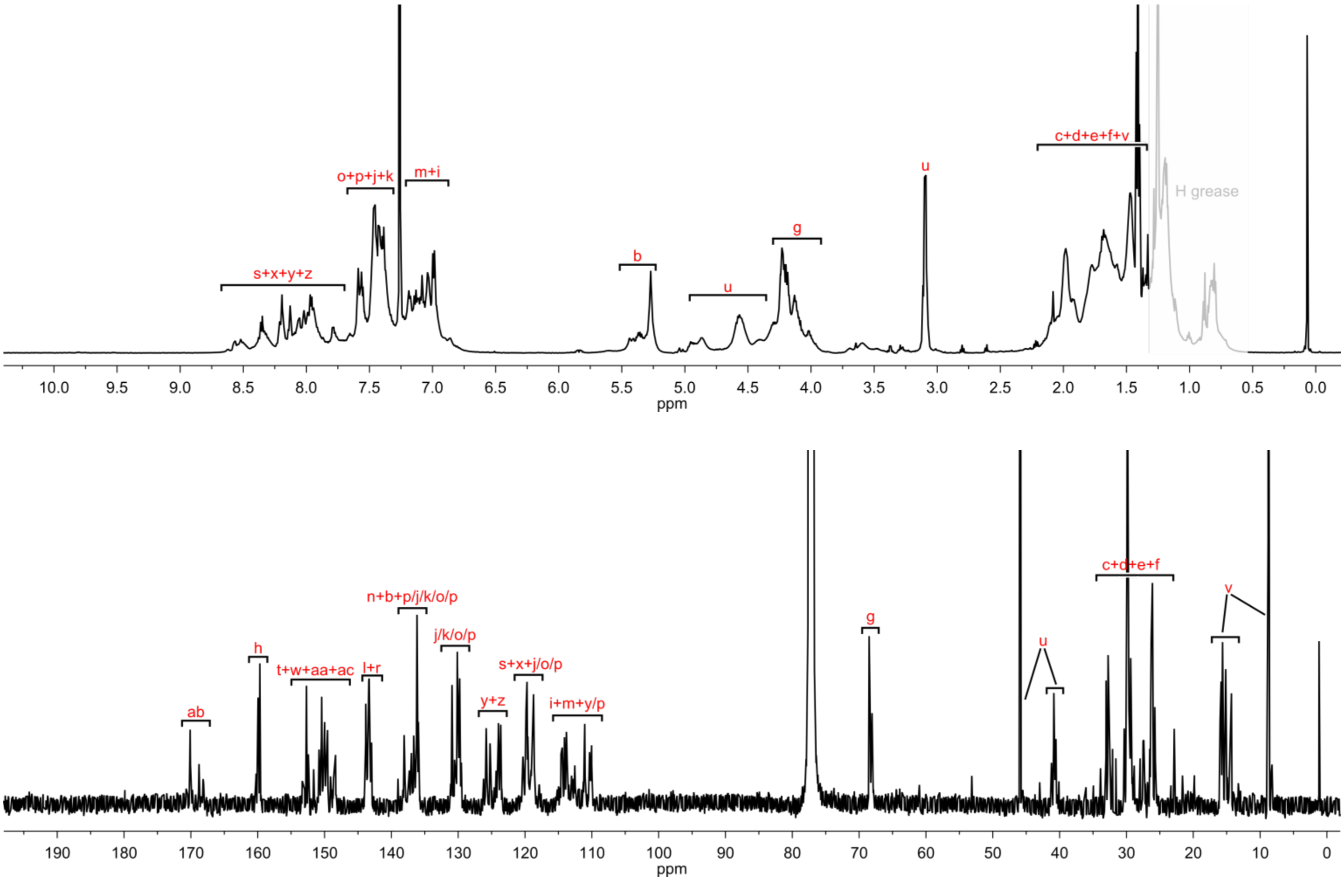

Figure S9. ^{1}H NMR (600 MHz, $CDCl_3$) spectrum (top), ^{13}C NMR (151 MHz, $CDCl_3$) spectrum (bottom) and peak assignment of pure **2**/**3** mixture after GPC purification (Figure S5, green fraction).

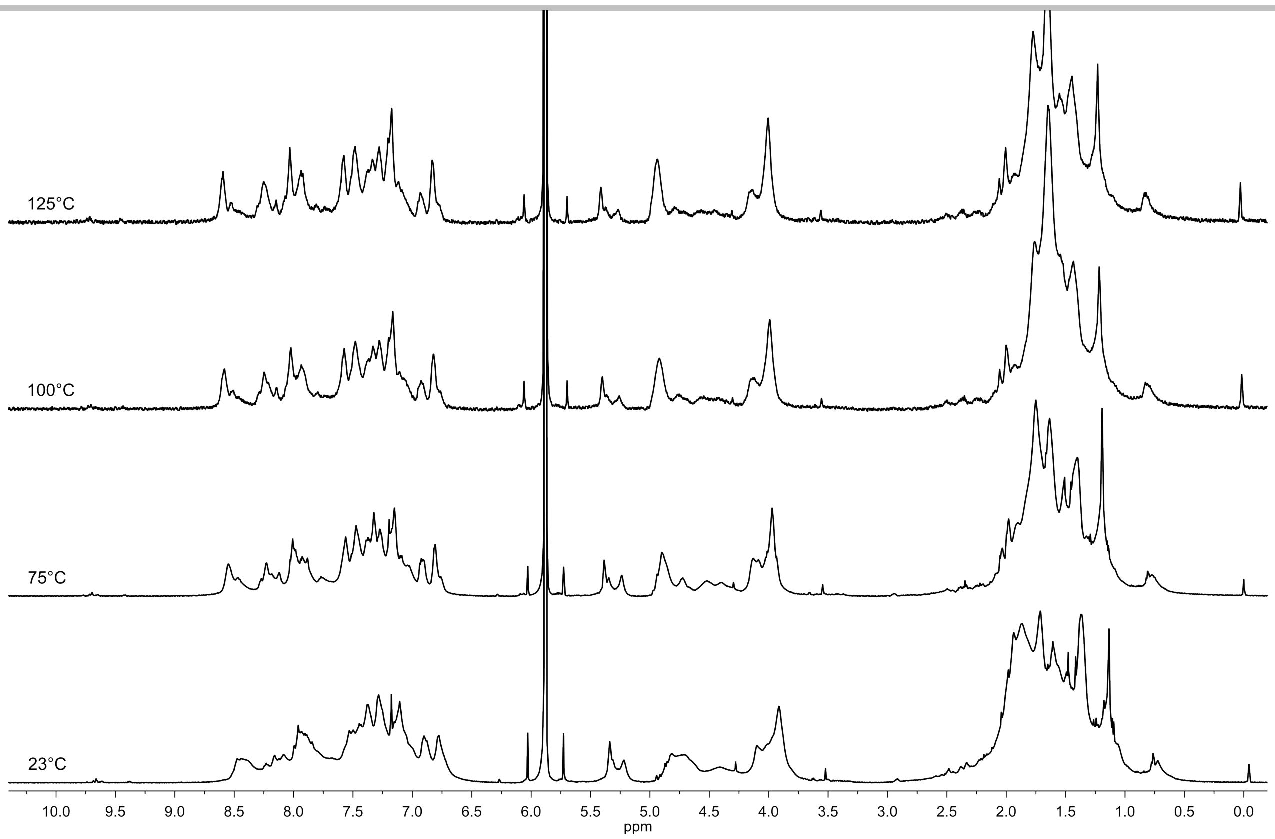

Figure S10. Stack plot of ^{1}H NMR spectra (500 MHz, tetrachloroethane-d_2) at different temperatures of pure **2**/**3** mixture after GPC purification (Figure S5, green fraction). The broadness of the spectra are due to slow reptation, typical of big entangled molecules.

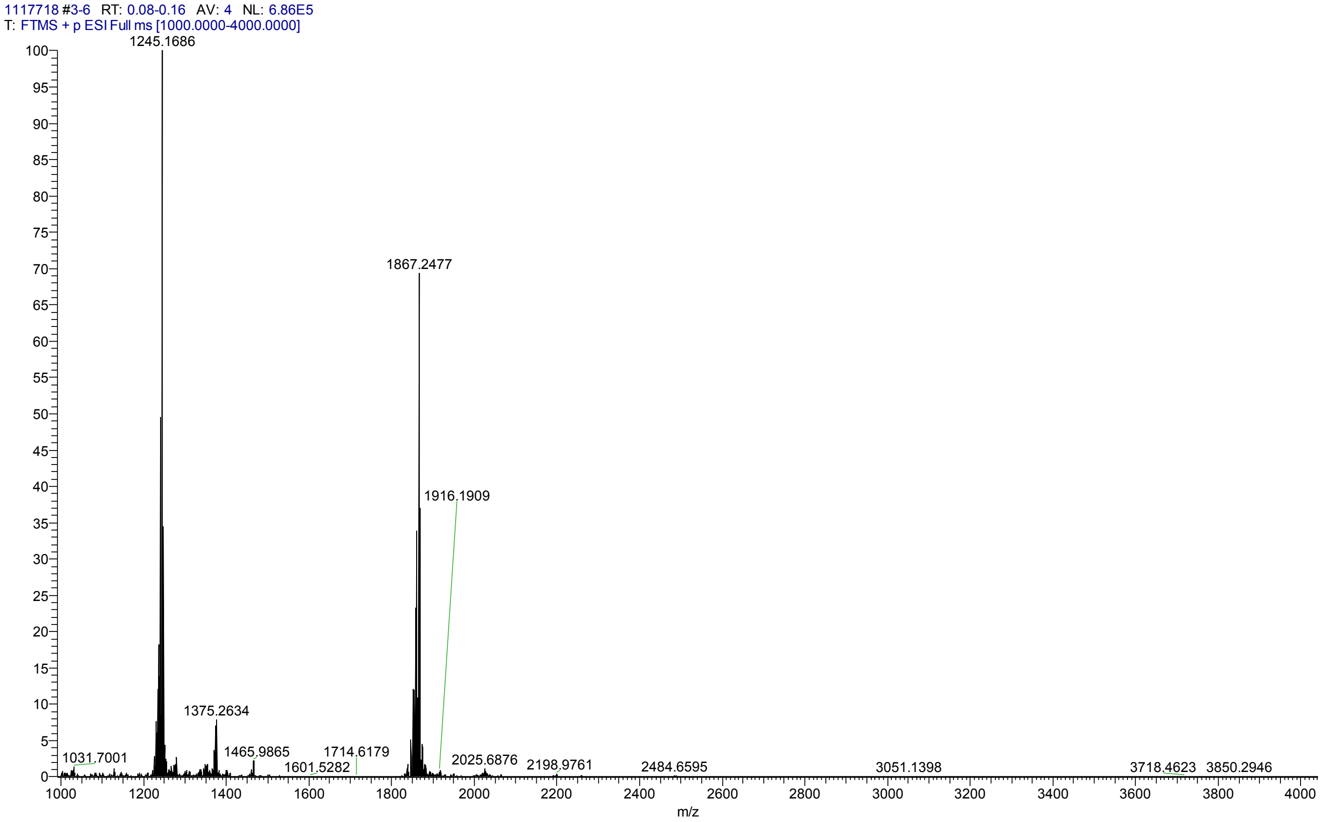

Figure S11. High-resolution ESI-MS of pure link **2**/knot **3** mixture after GPC separation (all peaks observed as the [**2**/**3**+nH]$^{n+}$ adducts). Calculated peaks (m/z): 1865.7 [M+2H]$^{2+}$; 1244.2 [M+3H]$^{3+}$.

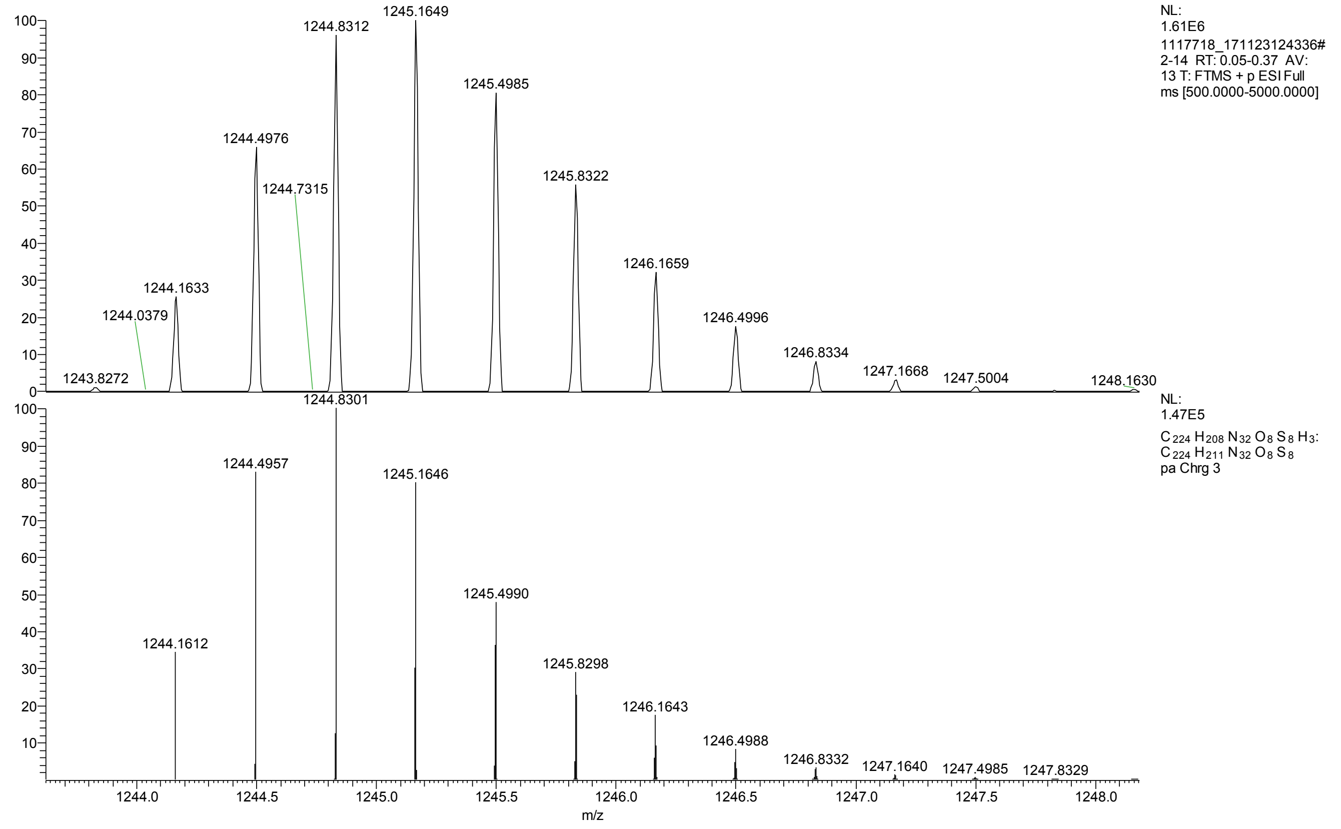

Figure S12. High-resolution ESI-MS of the $[M+3H]^{3+}$ peak of pure link **2**/knot **3** mixture after GPC separation. Experimental spectrum (top, observed m/z 1244.8312) and calculated spectrum (bottom, theoretical m/z 1244.8301).

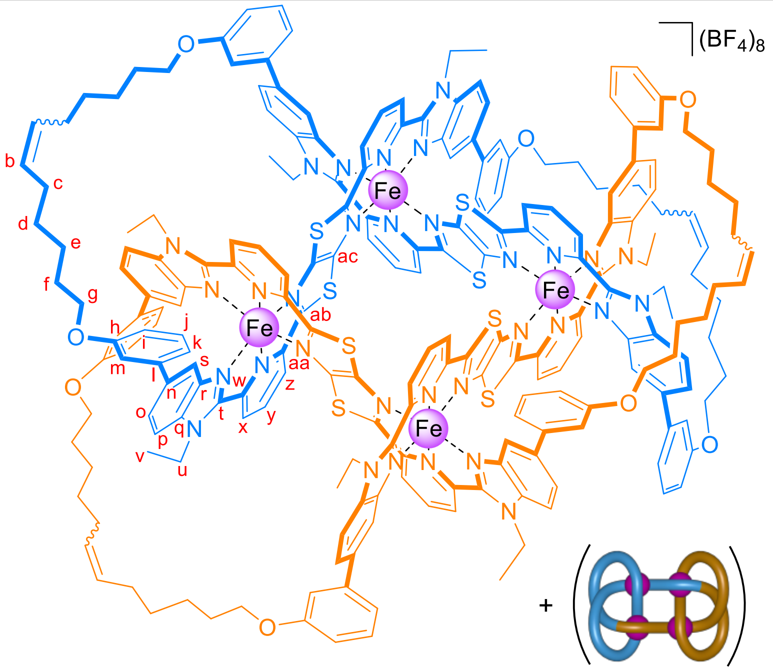

[Fe_4**2**/**3**]$(BF_4)_8$

2/**3** (10 mg, 2.68 μmol) was dissolved in $CHCl_3$ (1 mL), then iron(II) tetrafluoroborate hexahydrate (7.3 mg, 21.4 μmol) and MeCN (1 mL) were added. The mixture turned dark green almost instantely and it was stirred at 50°C for 24 h. The solvent was then removed under reduced pressure and the solid redissolved in MeCN (1 mL). The product was precipitated with a 0.1 M aqueous solution of KBF_4 and filtered over Celite®. After washing with water (50 mL), EtOH (5 mL) and Et_2O (10 mL), the product was taken into MeCN. Upon removal of the solvent under reduced pressure, [Fe_4**2**/**3**]$(BF_4)_8$ was obtained as a dark green solid (9.0 mg, 72%). ^{1}H NMR (600 MHz, Acetonitrile-d_3) δ 10.11 – 9.17 (H^x + H^z), 9.15 – 8.99 (H^y), 7.63 – 7.24 (H^j + H^o + H^p), 7.00 – 6.89 (H^k), 6.88 – 6.58 (H^i), 6.56 – 6.33 (H^m), 5.82 – 5.62 (H^b), 5.51 – 5.23 (H^s), 4.97 – 4.59 (H^u), 4.25 – 4.02 (H^g), 2.49 – 2.24 (H^c), 2.07 – 1.60 (H^d + H^e + Hf), 1.35 – 1.13 (H^v). ^{13}C NMR (151 MHz, Acetonitrile-d_3) δ 160.80 – 160.61 (C^h), 141.73 – 141.06 (C^l or C^n), 139.47 – 138.74 (C^l or C^n), 131.81 – 131.51 (C^b), 130.48 – 126.44 (C^j + C^o + C^p), 120.24 – 119.98 (C^i), 114.93 – 113.96 (C^m + C^k), 113.88 – 113.44 (C^s), 69.39 – 68.63 (C^g), 42.96 – 42.15 (C^u), 30.92 – 26.17 (C^d + C^e + C^f),16.64 – 15.77 (C^v). Carbons q, r, t, w, x, y, z, aa, ab, ac could not be unambiguously assigned. LR ESI-MS: m/z = 1463.0 $[M{-}3BF_4]^{3+}$ calculated 1462.7; 1075.6 $[M{-}4BF_4]^{4+}$ calculated 1075.3; 843.3 $[M{-}5BF_4]^{5+}$ calculated 842.8; 688.4 $[M{-}6BF_4]^{6+}$ calculated 687.9; 577.8 $[M{-}7BF_4]^{7+}$ calculated 577.2.

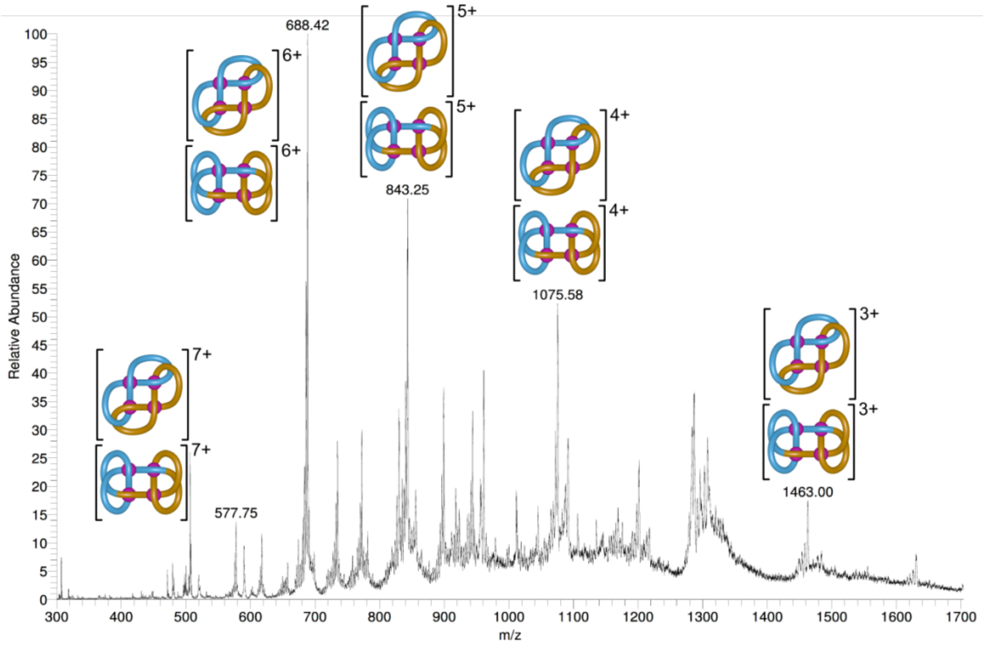

Figure S13. Low-resolution ESI-MS of [Fe_4**2**/**3**]$(BF_4)_8$ after remetallation of pure link **2**/knot **3** mixture (all peaks observed as the [Fe_4**2**/**3**+(8–n)$BF_4]^{n+}$ adducts). Calculated peaks (m/z): 1462.7 $[M{-}3BF_4]^{3+}$; 1075.3 $[M{-}4BF_4]^{4+}$; 842.8 $[M{-}5BF_4]^{5+}$; 687.9 $[M{-}6BF_4]^{6+}$; 577.2 $[M{-}7BF_4]^{7+}$.

2.3. GPC separation of topoisomers 2 and 3

Approximately 80 mg of the pure mixture of organic link **2** and knot **3** were subjected to purification by recycling GPC. The mixture, which appeared as a single sharp peak in the early cycles, required 6-7 cycles to show a noticeable splitting into two different peaks. After a minimum of approximately 15-17 cycles, the two peaks became sufficiently separated to be collected in different fractions (Figure S14). The recycling was interrupted between each pass of the peak in order to discard any smearing of the previous pass into the upcoming peak from the recycling operation. This procedure, although necessary, results in some loss of material. The isolated fractions, each enriched in one of the topoisomers, were resubmitted to GPC separation following the procedure described above (Figure S24 and S25) to ensure the removal of any traces of the other topoisomer. After exhaustive recycling, fractions containing pure link **2** (1.5 mg) and pure knot **3** (1.8 mg) were finally obtained. Both fractions were characterised by ^{1}H and ^{13}C NMR, where they afforded distinct but complex spectra (Figures S33-S35).

ESI-MS of the collected fractions (Figure S26 and S27) showed identical peaks to those of the mixture prior to separation (Figure S15). The starting mixture of topoisomers, as well as the separated fractions of link **2** (Figure S24) and knot **3** (Figure S25), were analysed through tandem ESI-MS by fragmentation of the triply charged $[M+3H]^{3+}$ peak. In the mixture ESI-MS/MS spectrum (Figure S16), a number of peaks from fragmentation of the base peak were observed, which were assigned to molecular fragments arising from both the link and the knot topologies based on the observed isotopic patterns for the detected peaks (Figures S17-S23). The fraction containing pure knot **3** gave a series of low intensity ESI-MS/MS peaks (Figure S30) that correspond to linear fragments that can only arise from the composite knot (fragmentation of the same bonds in link **2** would lead to dethreading and lower masses corresponding to non-interlocked macrocycle). Under the same conditions, fragmentation of the equivalent peak for the pure link (**2**) fraction afforded high intensity peaks coresponding to singly- and doubly-charged macrocycle (Figure S28) without the appearance of any higher mass fragments in significant amount, behaviour consistent with a link topology.

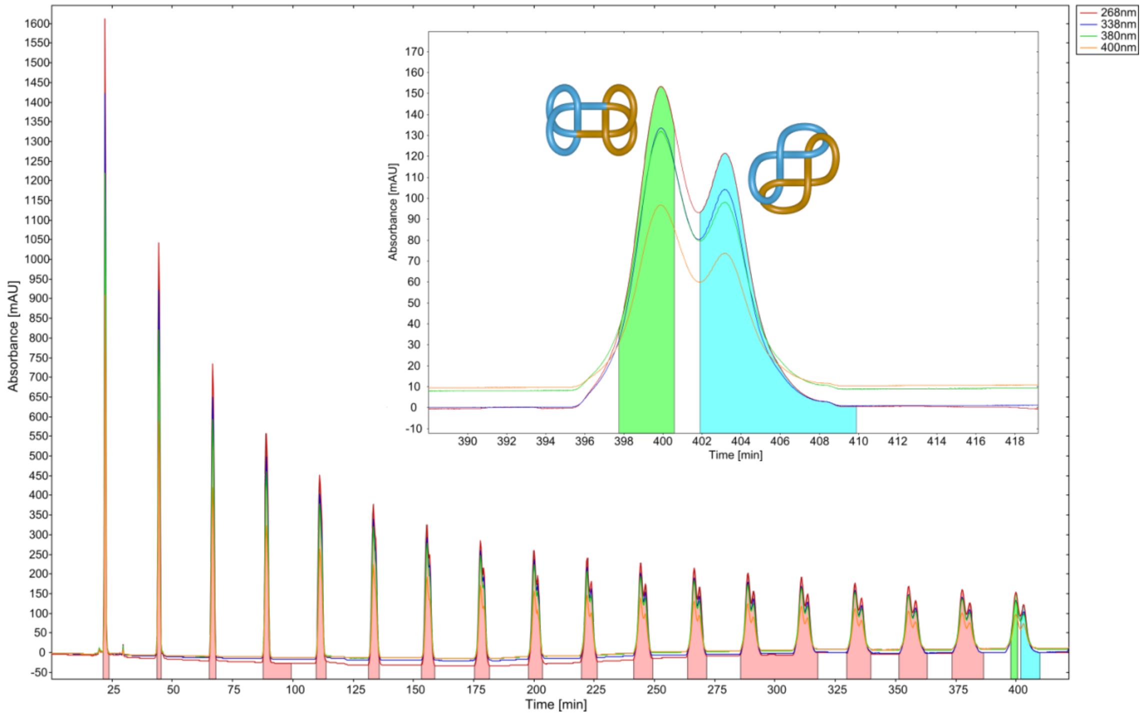

Figure S14. Representative recycling GPC UV-trace for the separation of link **2** (blue fraction) and knot **3** (green fraction). The red regions indicate the intervals of recycling during the elution. Both obtained fractions, which were each enriched in one of the desired topologies, were resubmitted to GPC separation to ensure purity (Figures S24 and S25), followed by the identification of the topoisomer present in each fraction by ESI-MS/MS experiments (Figures S28-S30).

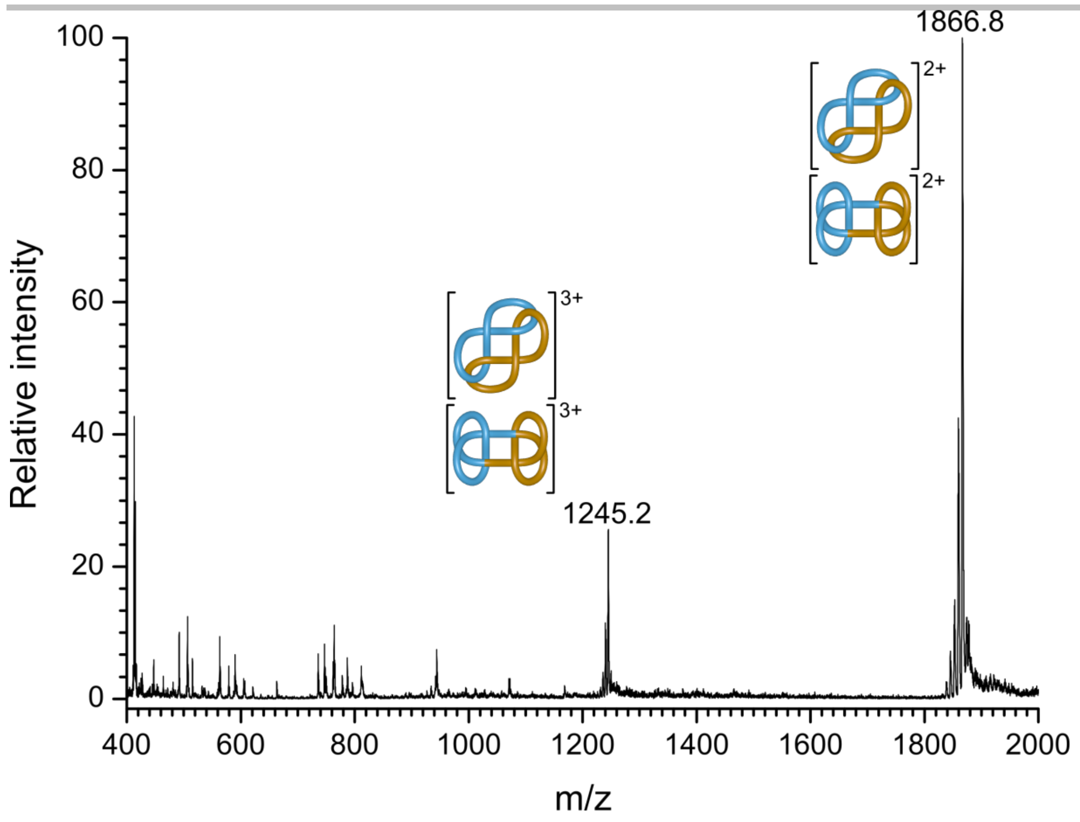

Figure S15. Low-resolution ESI-MS of pure link **2**/knot **3** mixture before further separation by GPC (all peaks observed as the [**2**/**3**+nH]$^{n+}$ adducts). Calculated peaks (m/z): 1865.7 [M+2H]$^{2+}$; 1244.2 [M+3H]$^{3+}$.

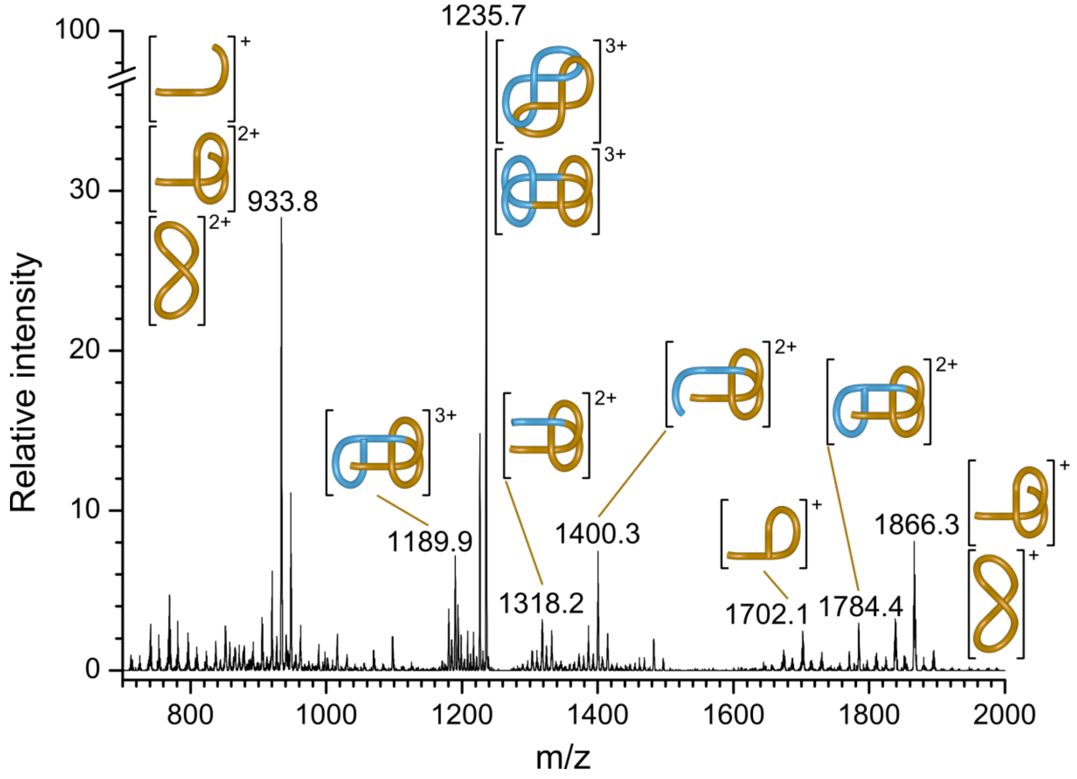

Figure S16. ESI-MS/MS experiment of pure link **2**/knot **3** mixture by fragmentation of the [M+3H]$^{3+}$ peak. The base peak at m/z 1235.7 corresponds to [M–C_2H_4+3H]$^{3+}$, where one of the pendant ethyl groups on the benzimidazole moiety has been lost as ethylene; all other peaks are observed as the corresponding [M+nH]$^{n+}$ adducts. The identity of the fragments was assigned by analysis of the isotopic patterns for the detected peaks (Figures S17-S23).

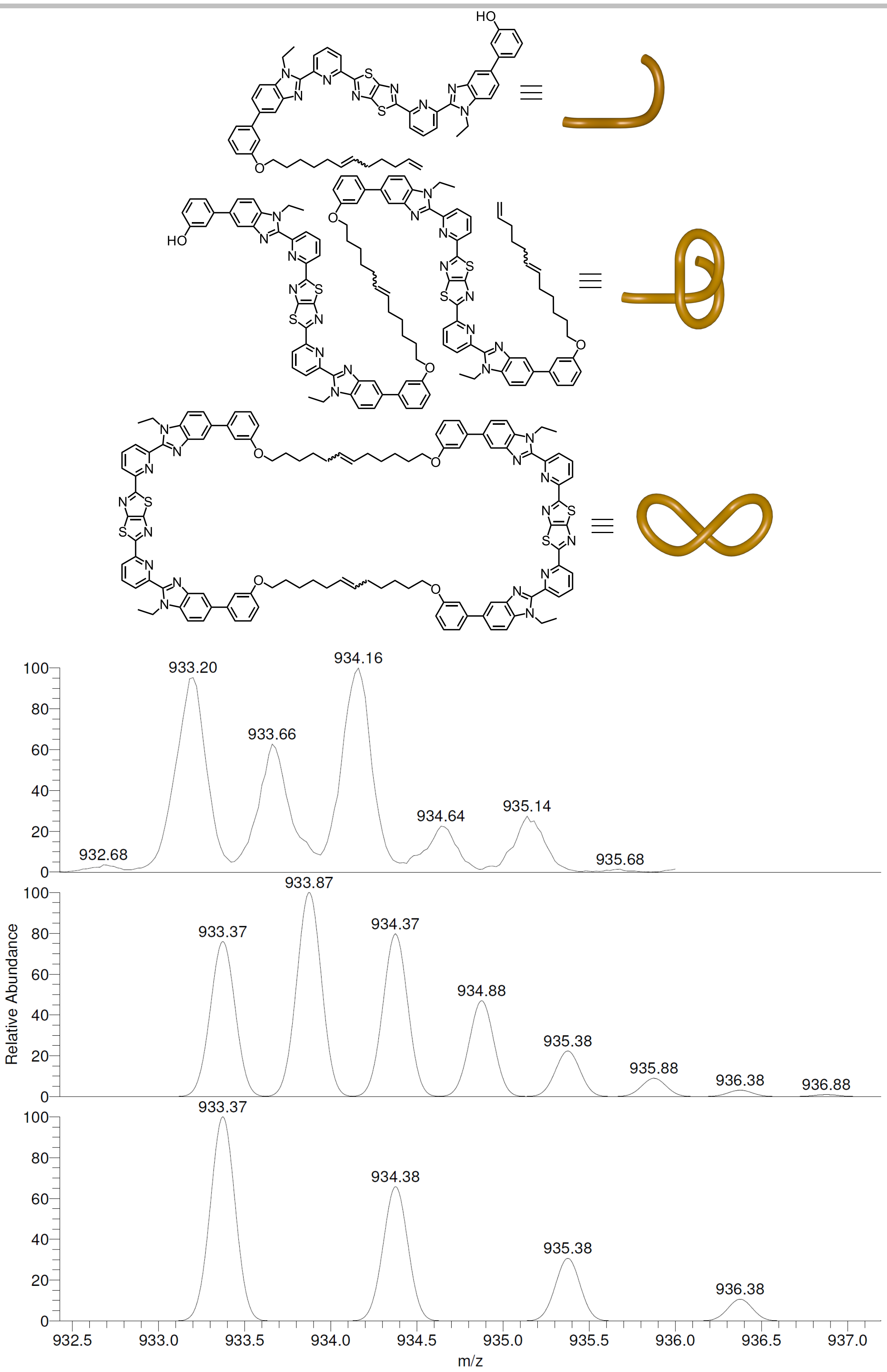

Figure S17. Proposed structure for the fragments with m/z 934, and low resolution ESI-MS/MS of the m/z 934 peak from pure link **2**/knot **3** mixture by fragmentation of the $[M+3H]^{3+}$ peak. Experimental spectrum (top), and calculated spectra for the proposed doubly charged (middle, predicted for $[C_{112}H_{104}N_{16}O_4S_4+2H]^{2+}$) and singly charged fragments (bottom, predicted for $[C_{56}H_{52}N_8O_2S_2+H]^{+}$).

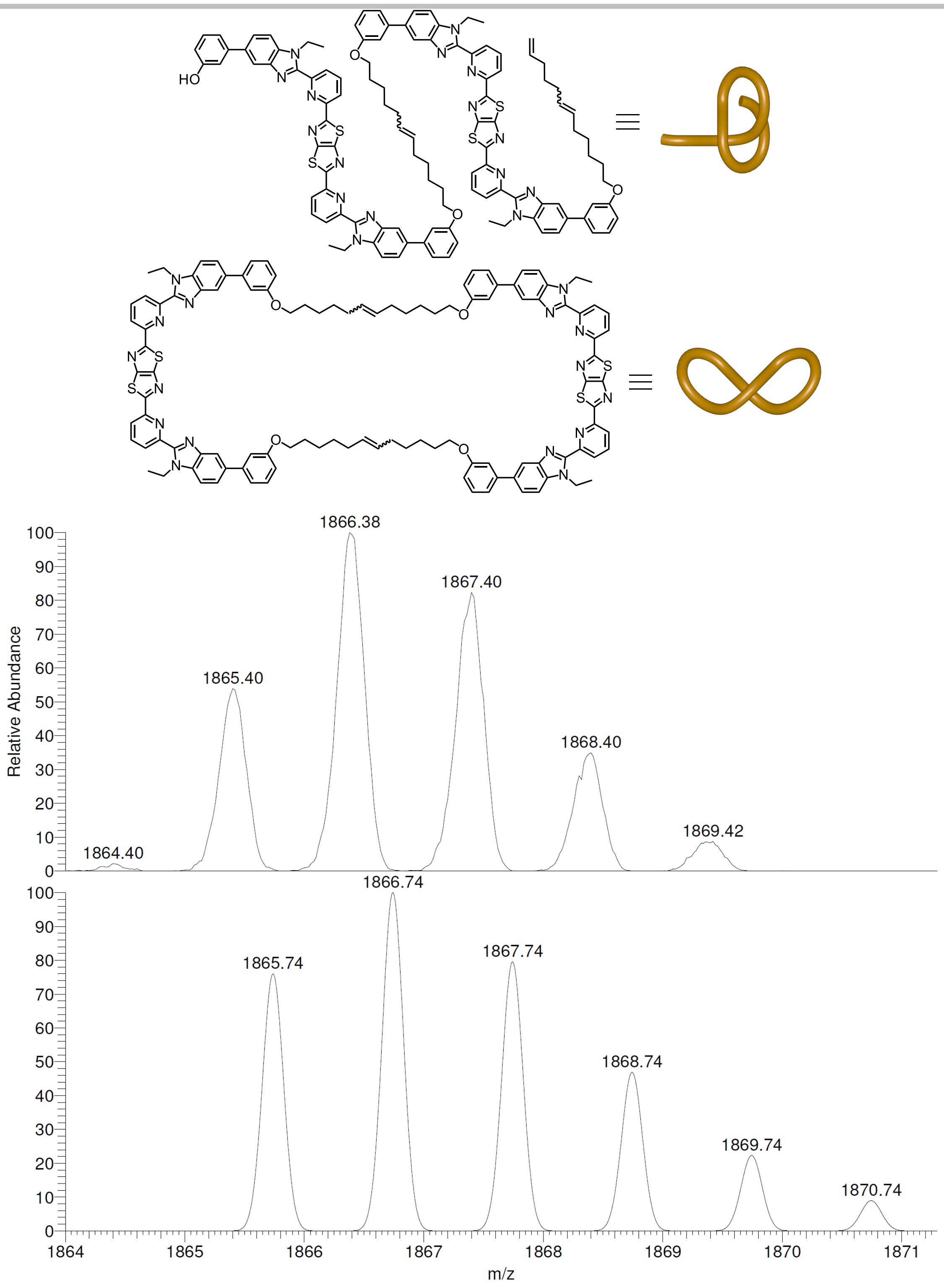

Figure S18. Proposed structure for the fragments with m/z 1866, and low resolution ESI-MS/MS of the m/z 1866 peak from pure link **2**/knot **3** mixture by fragmentation of the $[M+3H]^{3+}$ peak. Experimental spectrum (top), and calculated spectrum for the proposed singly charged fragments (bottom, predicted for $[C_{112}H_{104}N_{16}O_4S_4+H]^+$).

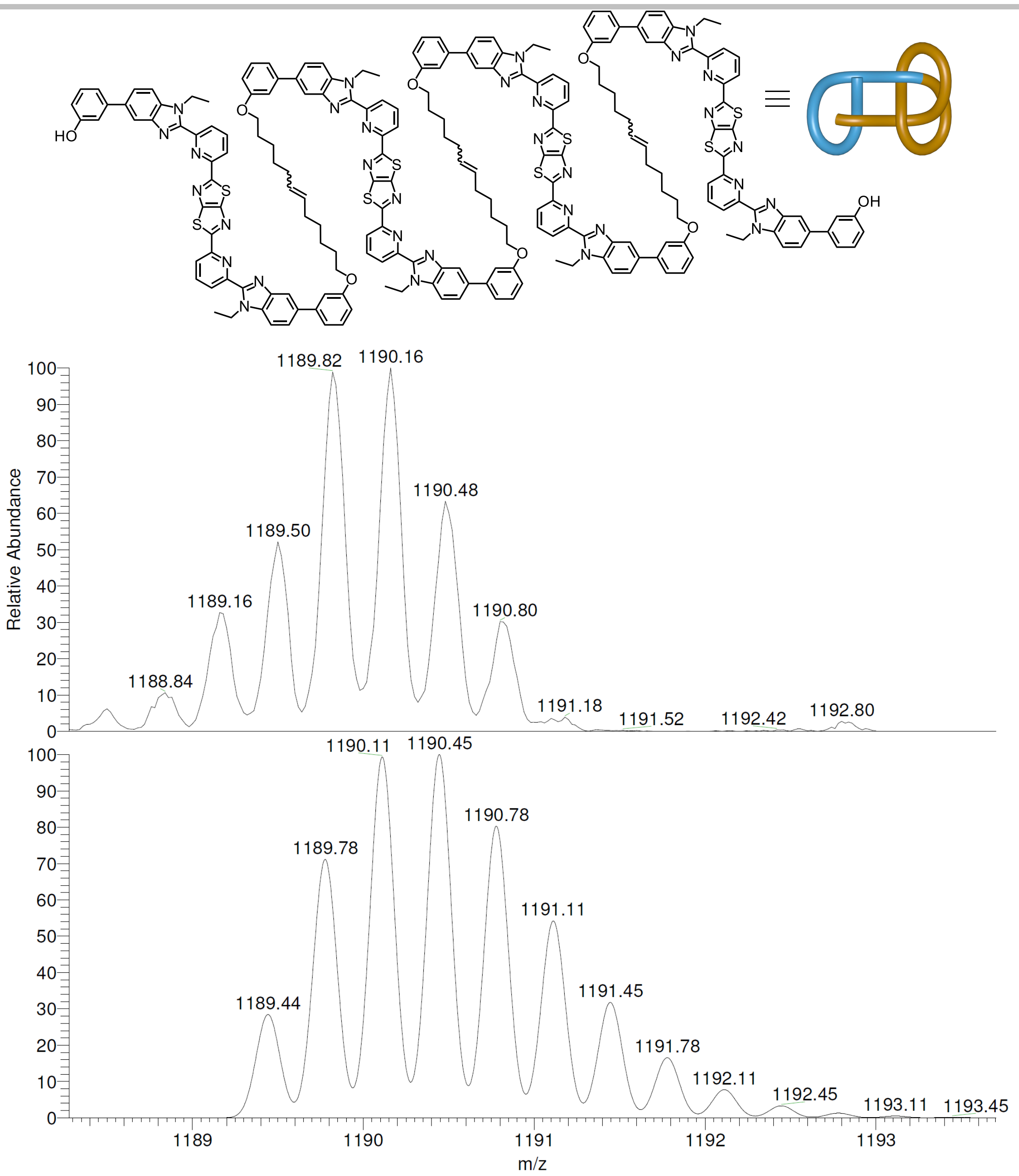

Figure S19. Proposed structure for the fragment with m/z 1190, and low resolution ESI-MS/MS of the m/z 1190 peak from pure link **2**/knot **3** mixture by fragmentation of the $[M+3H]^{3+}$ peak. Experimental spectrum (top), and calculated spectrum for the proposed triply charged fragment (bottom, predicted for $[C_{212}H_{188}N_{32}O_8S_8+3H]^{3+}$).

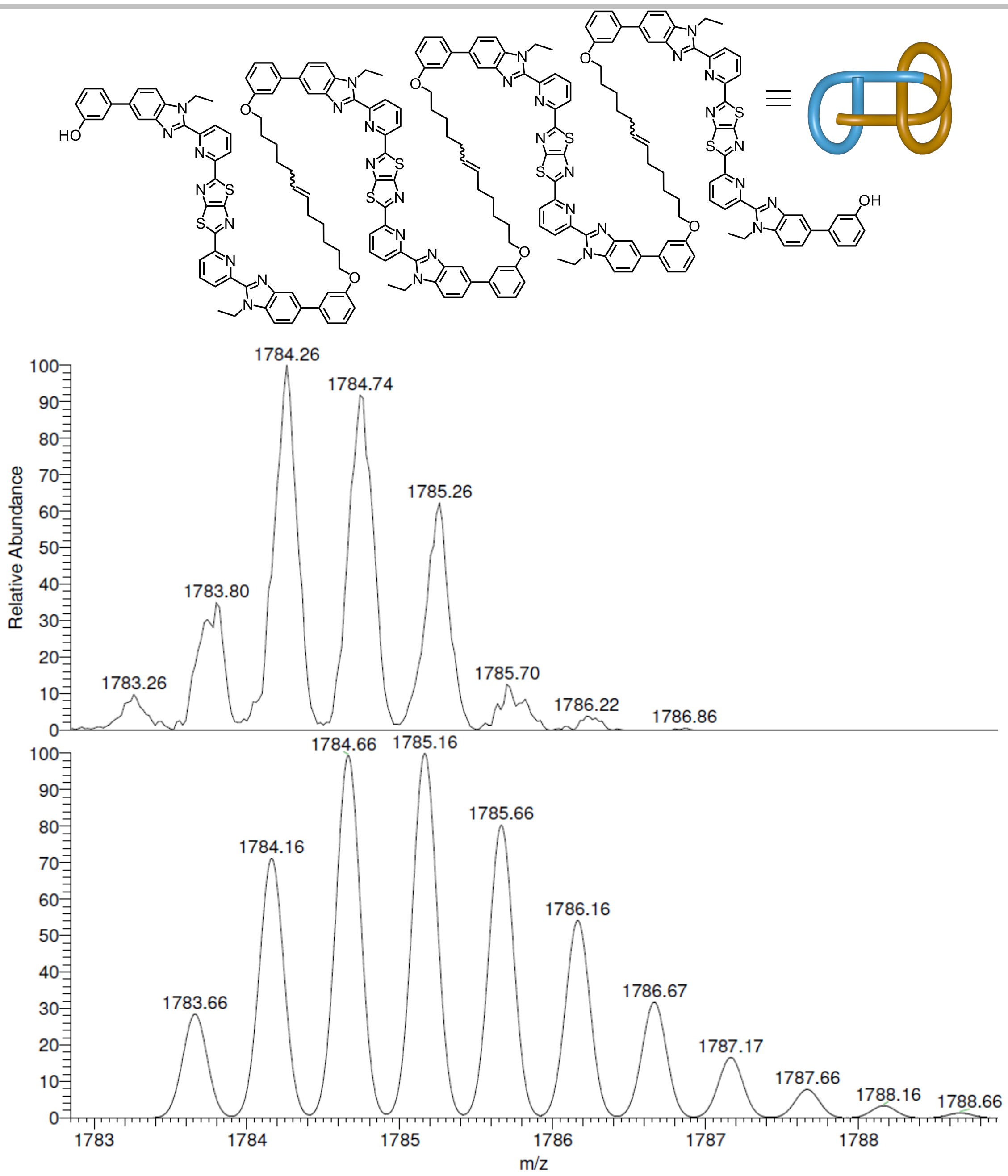

Figure S20. Proposed structure for the fragment with m/z 1784, and low resolution ESI-MS/MS of the m/z 1784 peak from pure link **2**/knot **3** mixture by fragmentation of the $[M+3H]^{3+}$ peak. Experimental spectrum (top), and calculated spectrum for the proposed doubly charged fragment (bottom, predicted for $[C_{212}H_{188}N_{32}O_8S_8+2H]^{2+}$).

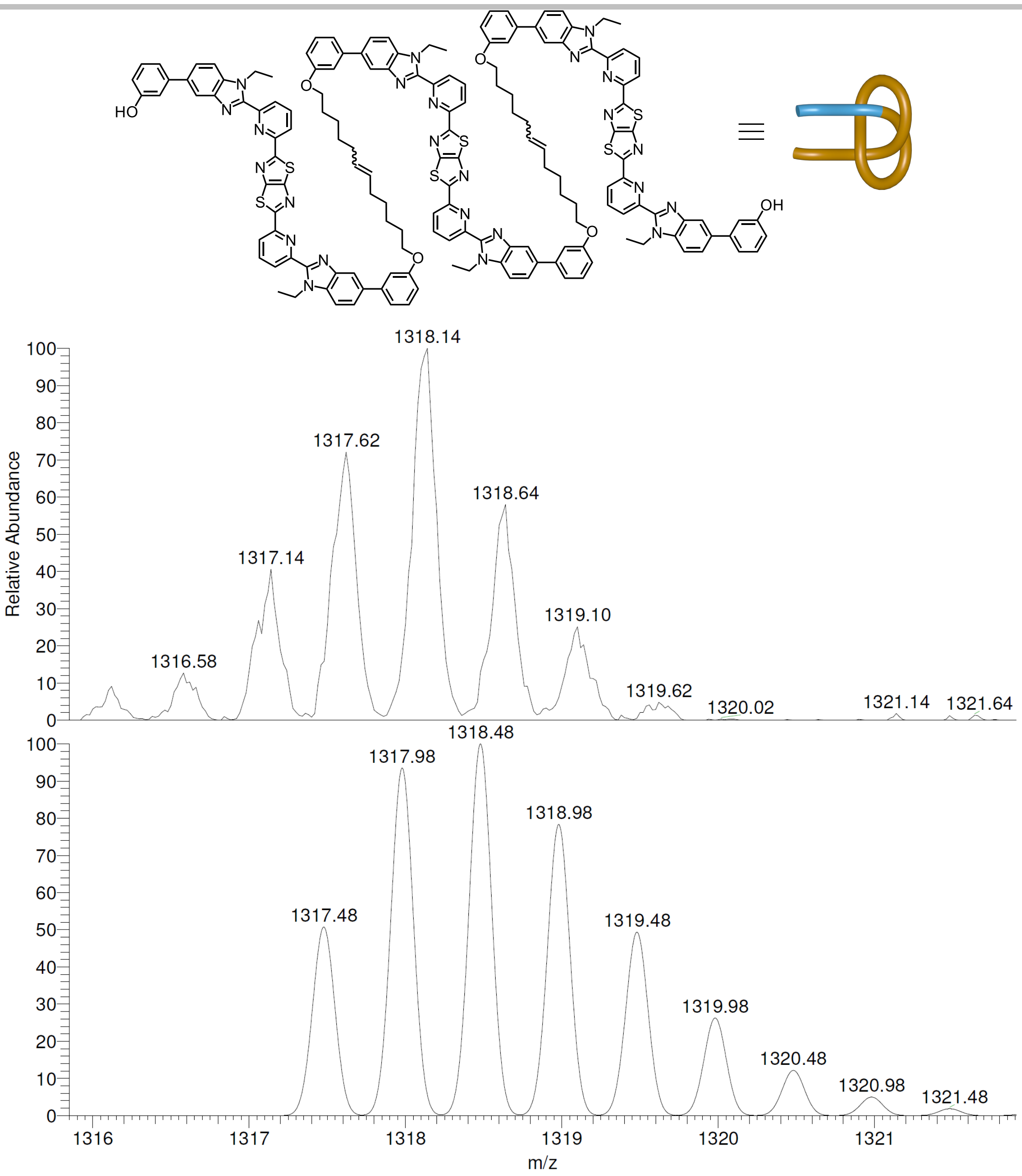

Figure S21. Proposed structure for the fragment with m/z 1318, and low resolution ESI-MS/MS of the m/z 1318 peak from pure link **2**/knot **3** mixture by fragmentation of the $[M+3H]^{3+}$ peak. Experimental spectrum (top), and calculated spectrum for the proposed doubly charged fragment (bottom, predicted for $[C_{156}H_{136}N_{24}O_6S_6+2H]^{2+}$).

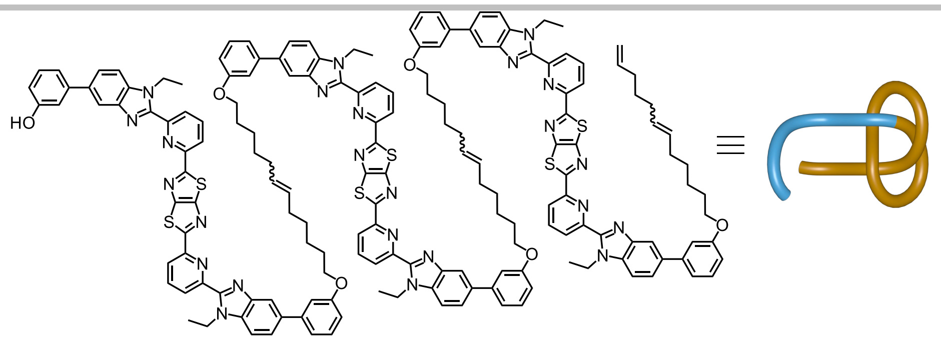

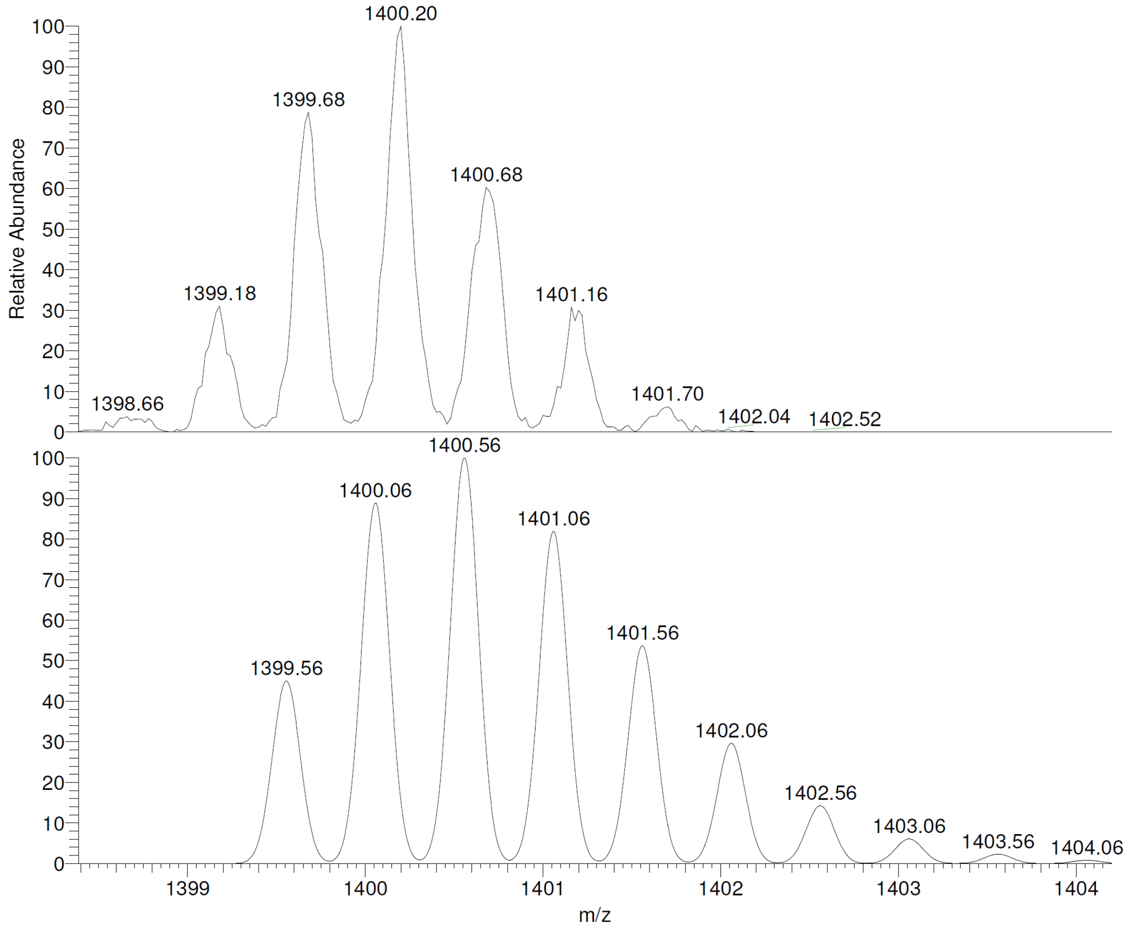

Figure S22. Proposed structure for the fragment with m/z 1400, and low resolution ESI-MS/MS of the m/z 1400 peak from pure link **2**/knot **3** mixture by fragmentation of the $[M+3H]^{3+}$ peak. Experimental spectrum (top), and calculated spectrum for the proposed doubly charged fragment (bottom, predicted for $[C_{168}H_{156}N_{24}O_6S_6+2H]^{2+}$).

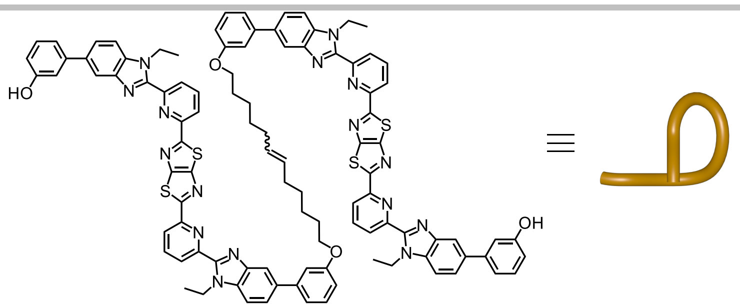

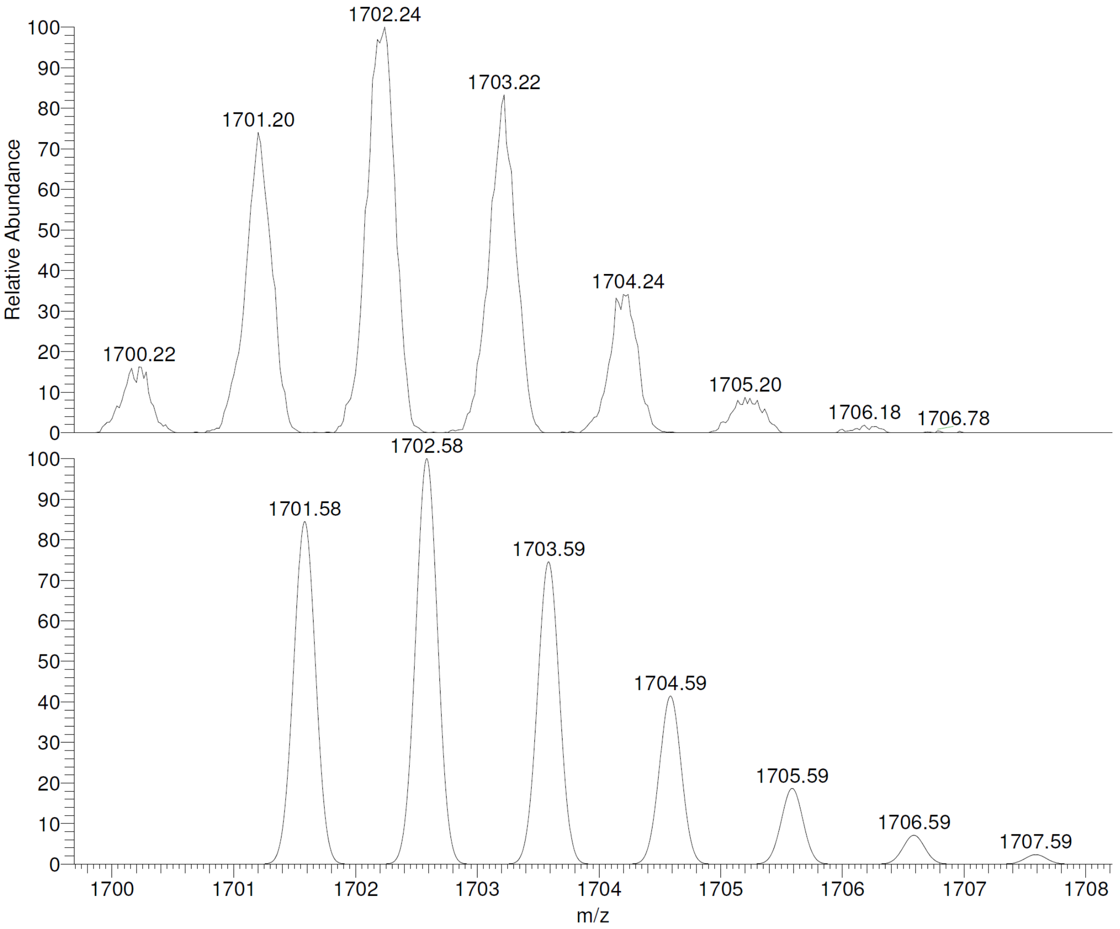

Figure S23. Proposed structure for the fragment with m/z 1702, and low resolution ESI-MS/MS of the m/z 1702 peak from pure link **2**/knot **3** mixture by fragmentation of the $[M+3H]^{3+}$ peak. Experimental spectrum (top), and calculated spectrum for the proposed singly charged fragment (bottom, predicted for $[C_{100}H_{84}N_{16}O_4S_4+H]^+$).

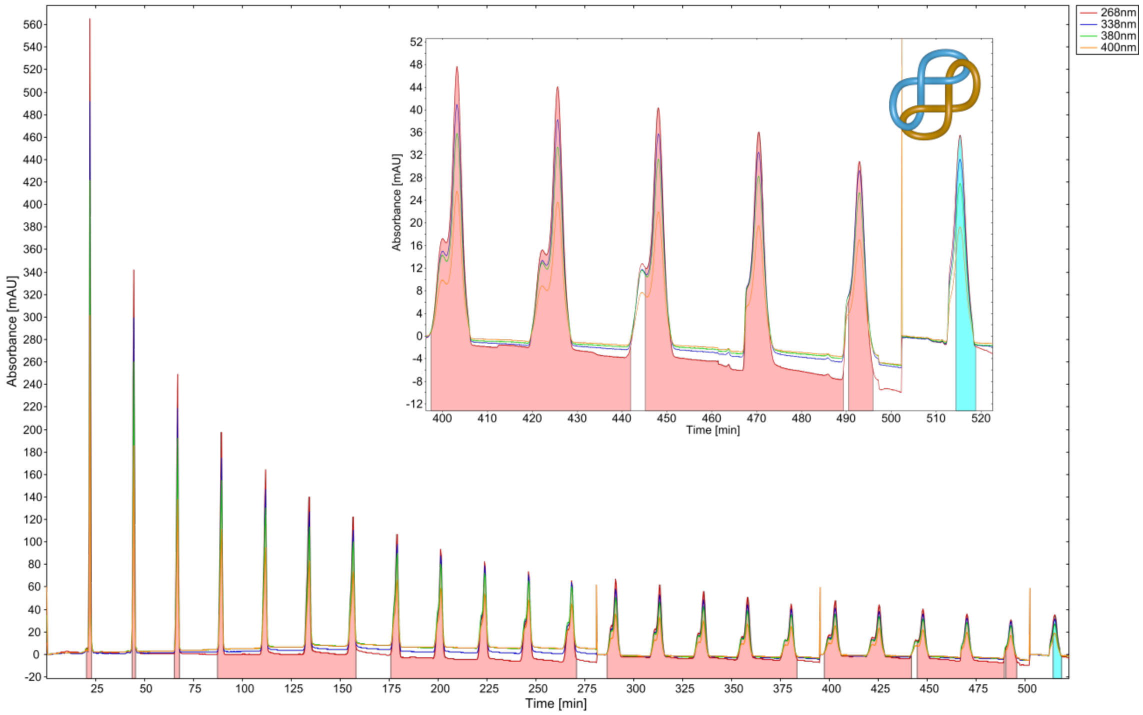

Figure S24. Representative recycling GPC UV-trace for the further purification of link **2** (Figure S14, blue fraction). The red regions indicate the intervals of recycling during the elution. A fraction containing pure link **2** was collected (blue fraction), and its identity was determined by ESI-MS/MS experiments (Figures S28 and S29).

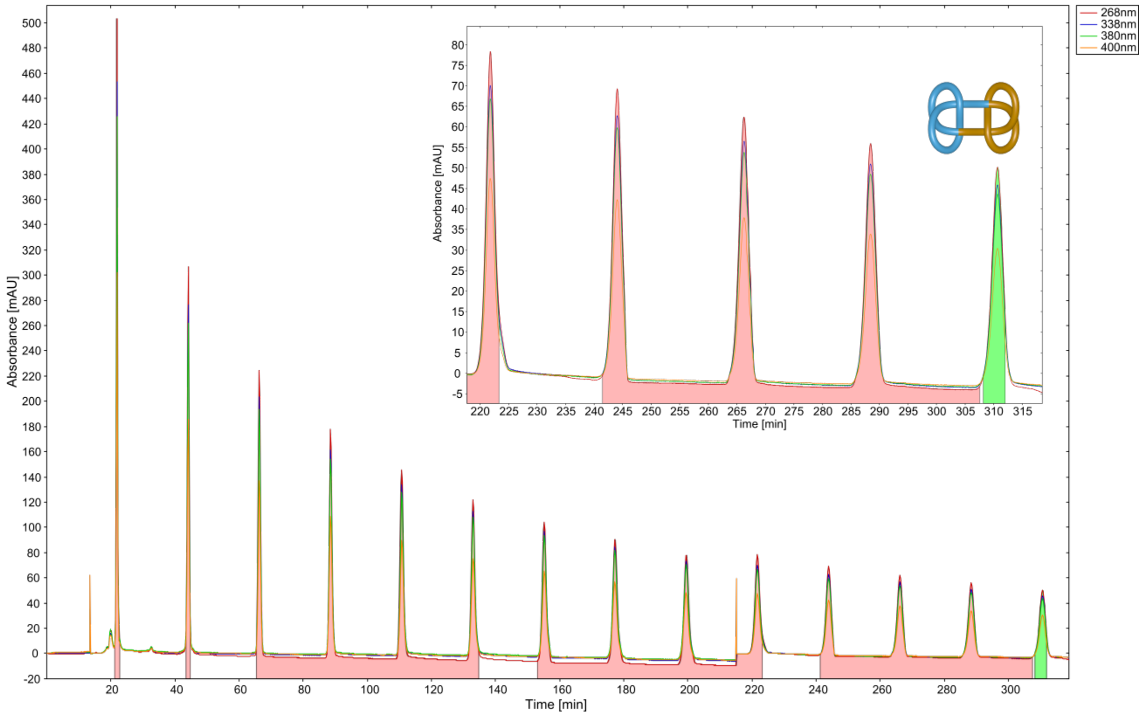

Figure S25. Representative recycling GPC UV-trace for the further purification of knot **3** (Figure S14, green fraction). The red regions indicate the intervals of recycling during the elution. A fraction containing pure knot **3** was collected (green fraction), and its identity was determined by ESI-MS/MS experiments (Figures S30 and S31).

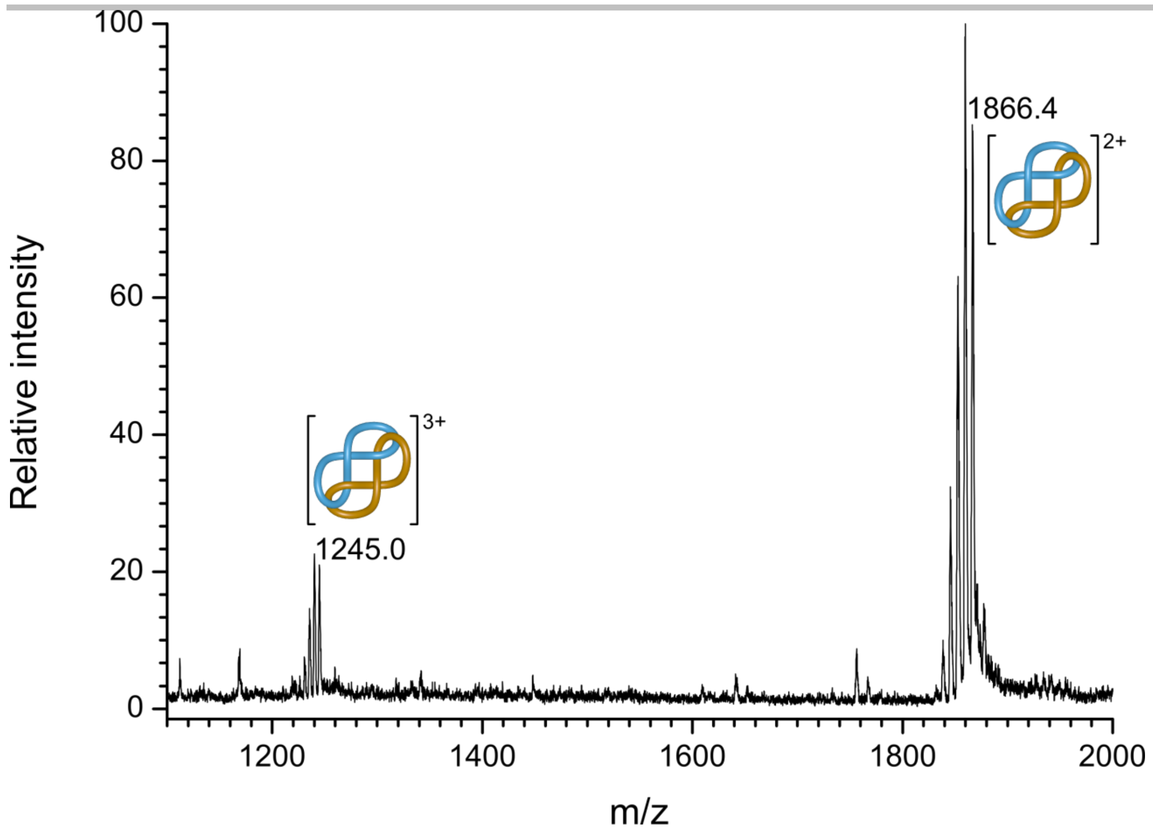

Figure S26. Low-resolution ESI-MS of pure link **2** (Figure S24, blue fraction; all peaks observed as the [**2**+nH]$^{n+}$ adducts). Calculated peaks (m/z): 1865.7 [M+2H]$^{2+}$; 1244.2 [M+3H]$^{3+}$.

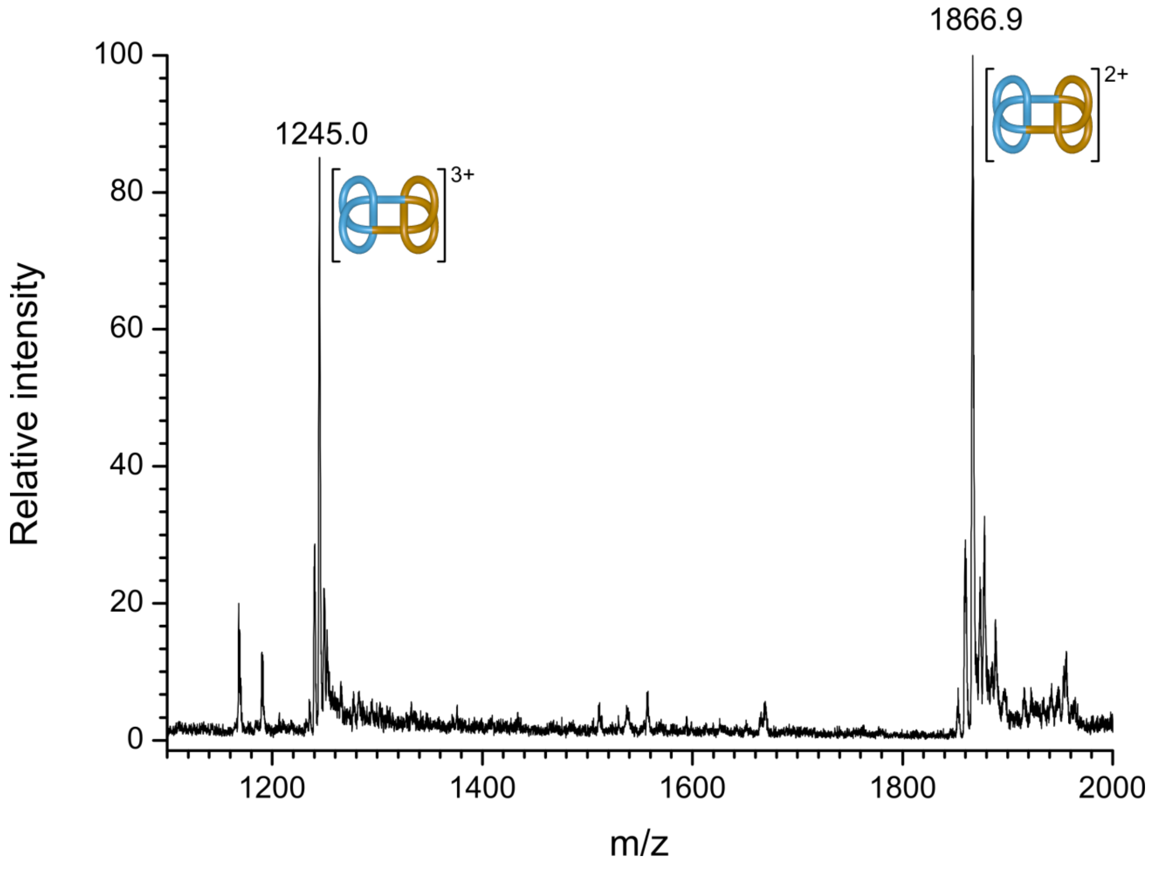

Figure S27. Low-resolution ESI-MS of pure knot **3** (Figure S25, green fraction; all peaks observed as the [**3**+nH]$^{n+}$ adducts). Calculated peaks (m/z): 1865.7 [M+2H]$^{2+}$; 1244.2 [M+3H]$^{3+}$.

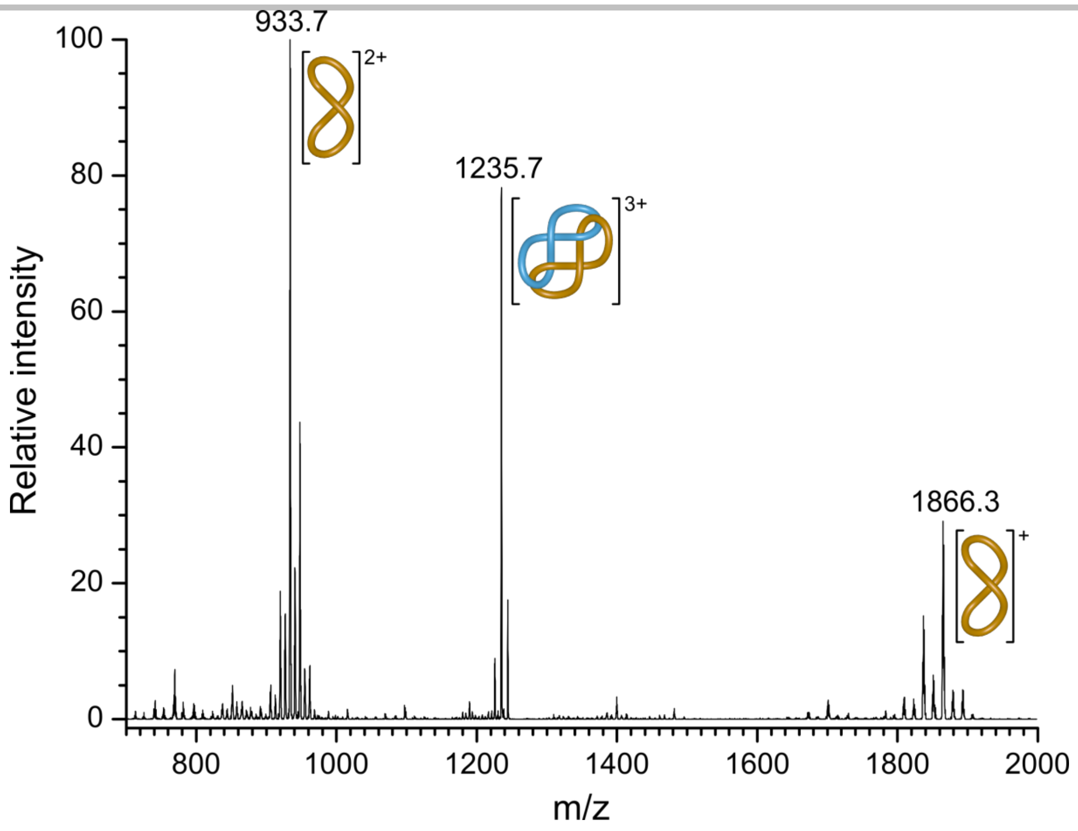

Figure S28. ESI-MS/MS experiment of pure link **2** by fragmentation of the $[M+3H]^{3+}$ peak. The base peak at m/z 1235.7 corresponds to $[M–C_2H_4+3H]^{3+}$, where one of the pendant ethyl groups on the benzimidazole moiety has been lost as ethylene; all other peaks are observed as the corresponding $[M+nH]^{n+}$ adducts.

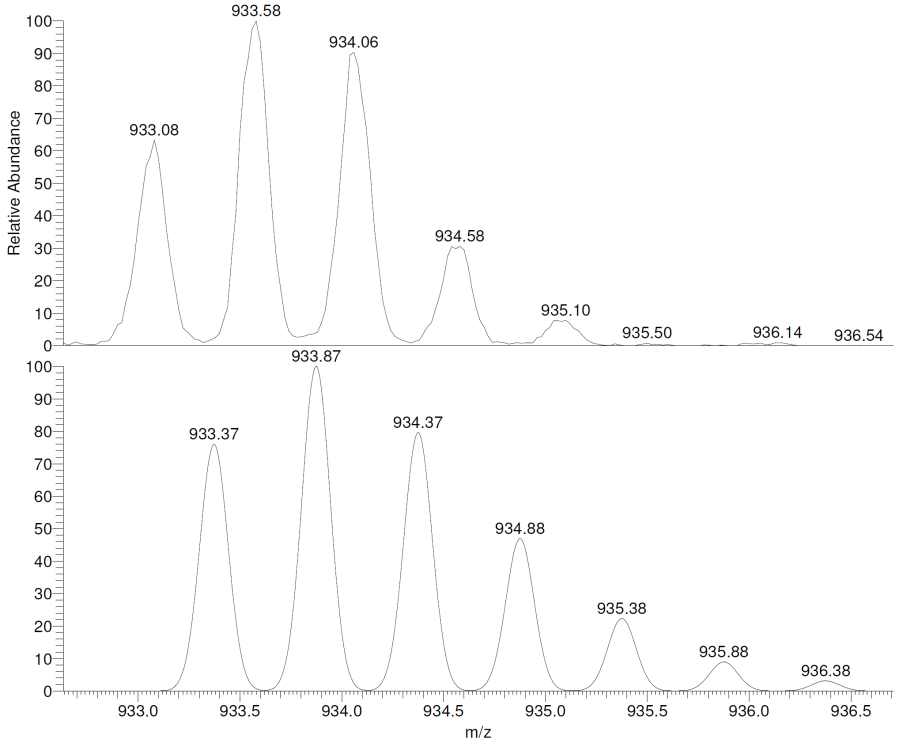

Figure S29. Low resolution ESI-MS/MS of the m/z 934 peak from pure link **2** by fragmentation of the $[M+3H]^{3+}$ peak. Experimental spectrum (top), and calculated spectrum (bottom, predicted for macrocycle $[C_{112}H_{104}N_{16}O_4S_4+2H]^{2+}$). Note the isotopic pattern of m/z 934 for the pure link **2** corresponding exclusively to doubly charged species, in contrast to the pattern for the mixture of topoisomers (Figure S17).

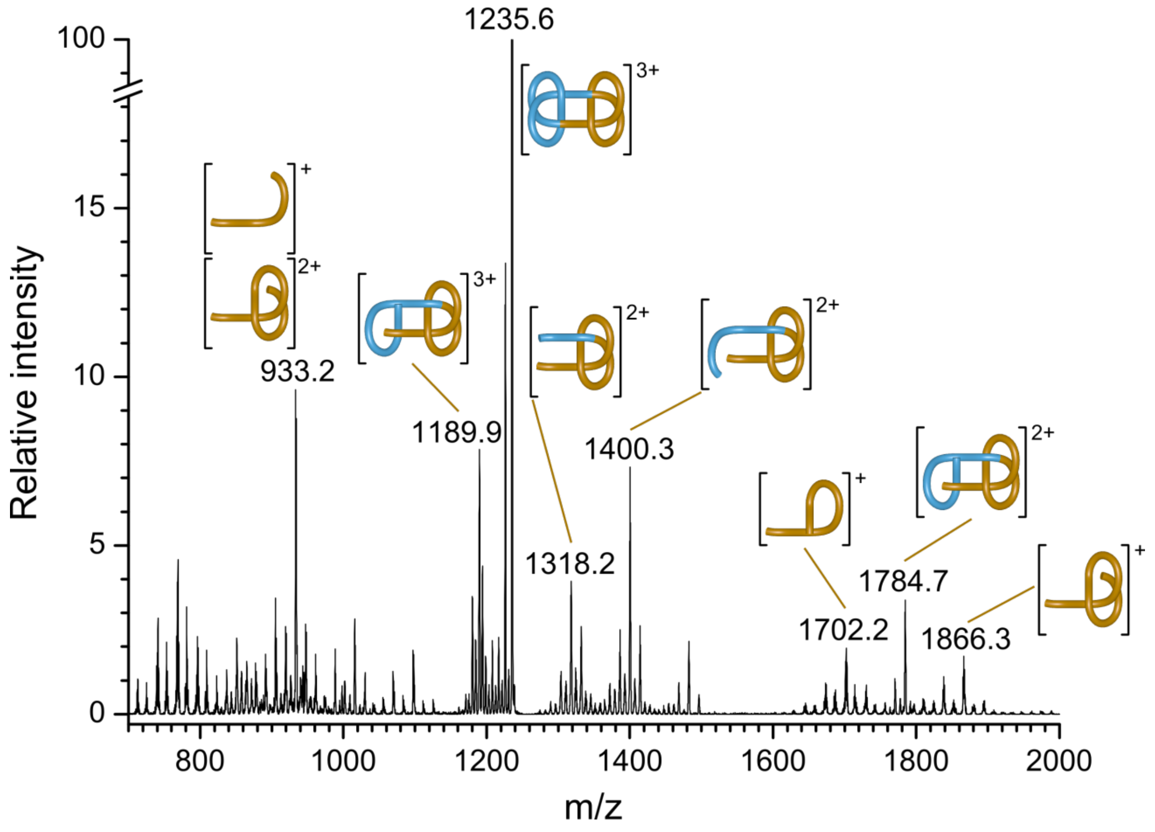

Figure S30. ESI-MS/MS experiment of pure knot **3** by fragmentation of the $[M+3H]^{3+}$ peak. The base peak at m/z 1235.6 corresponds to $[M–C_2H_4+3H]^{3+}$, where one of the pendant ethyl groups on the benzimidazole moiety has been lost as ethylene; all other peaks are observed as the corresponding $[M+nH]^{n+}$ adducts.

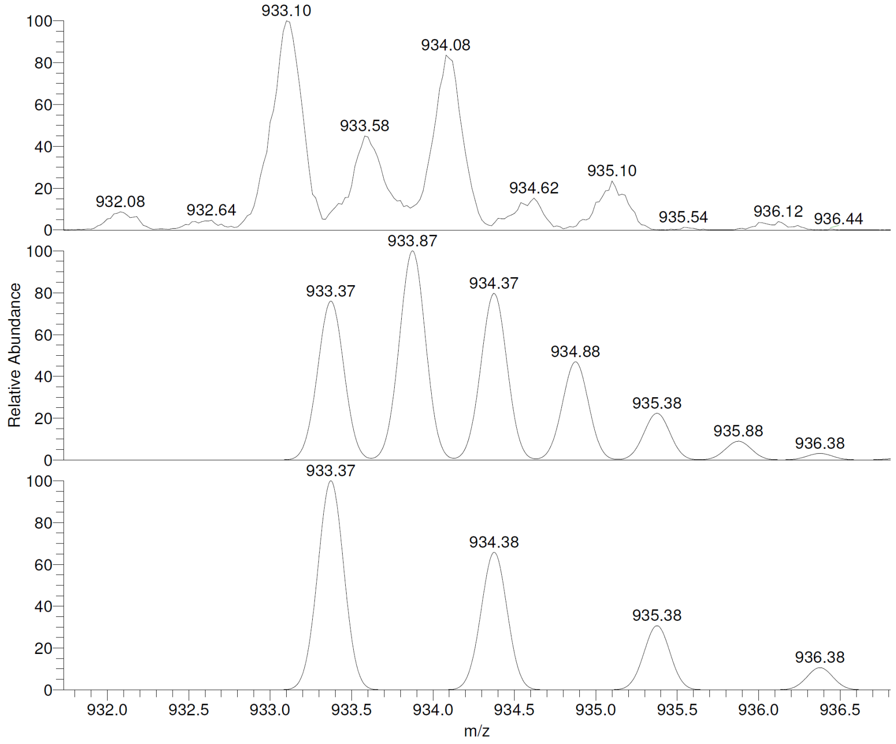

Figure S31. Low resolution ESI-MS/MS of the m/z 934 peak from pure knot **3** by fragmentation of the $[M+3H]^{3+}$ peak. Experimental spectrum (top), and calculated spectra for the proposed doubly charged (middle, predicted for linear fragment $[C_{112}H_{104}N_{16}O_4S_4+2H]^{2+}$) and singly charged fragments (bottom, predicted for linear fragment $[C_{56}H_{52}N_8O_2S_2+H]^{+}$). Note the difference in the relative intensities of the peaks corresponding to singly and doubly charged species in the pattern of pure knot **3**, in contrast to the pattern for the mixture of topoisomers (Figure S17).

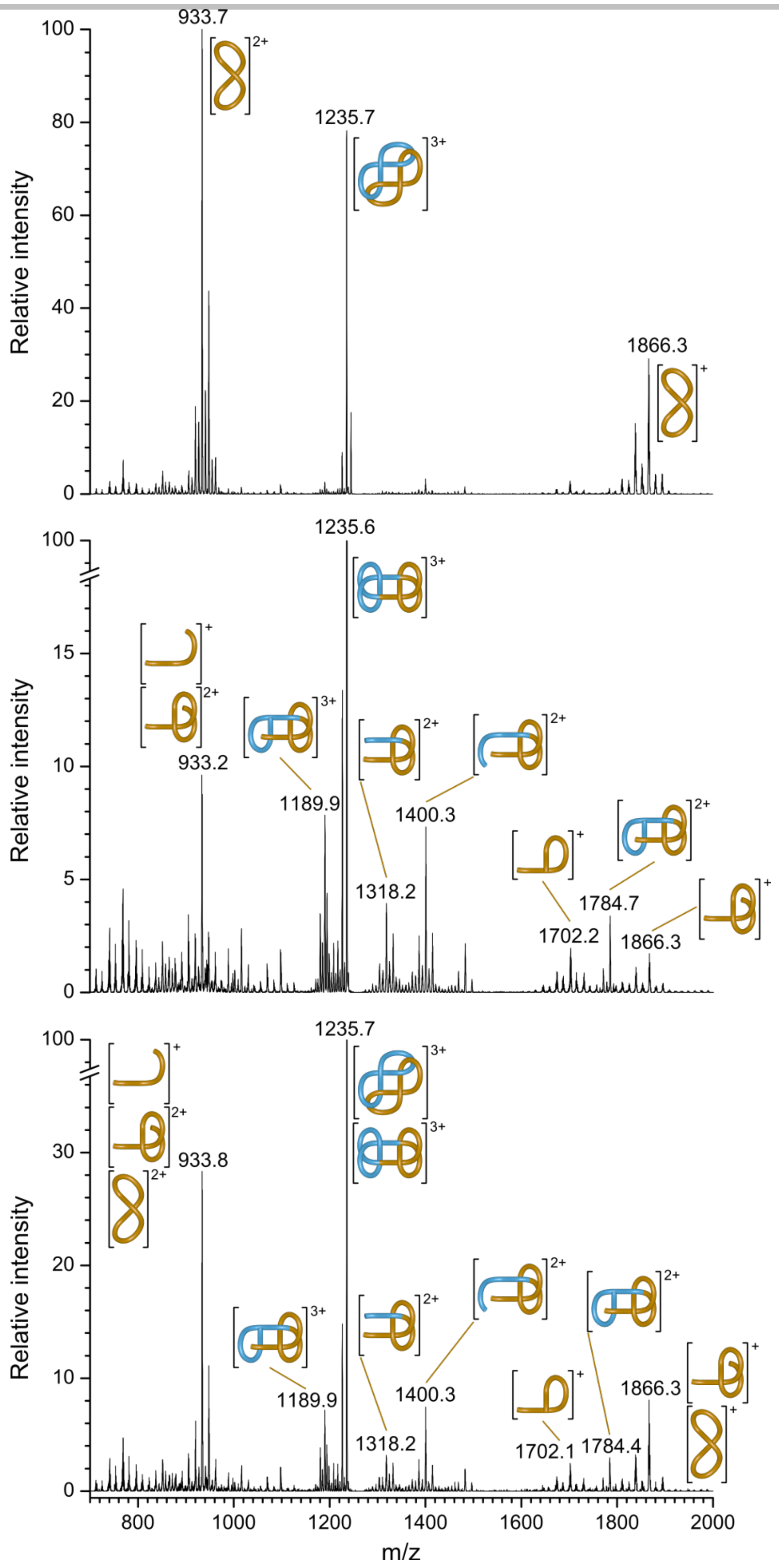

Figure S32. ESI-MS/MS experiments of pure link **2** (top), pure knot **3** (middle) and link **2**/knot **3** mixture (bottom) by fragmentation of the $[M+3H]^{3+}$ peak. The base peaks at m/z 1235 correspond to $[M–C_2H_4+3H]^{3+}$, where one of the pendant ethyl groups on the benzimidazole moiety has been lost as ethylene; all other peaks are observed as the corresponding $[M+nH]^{n+}$ adducts.

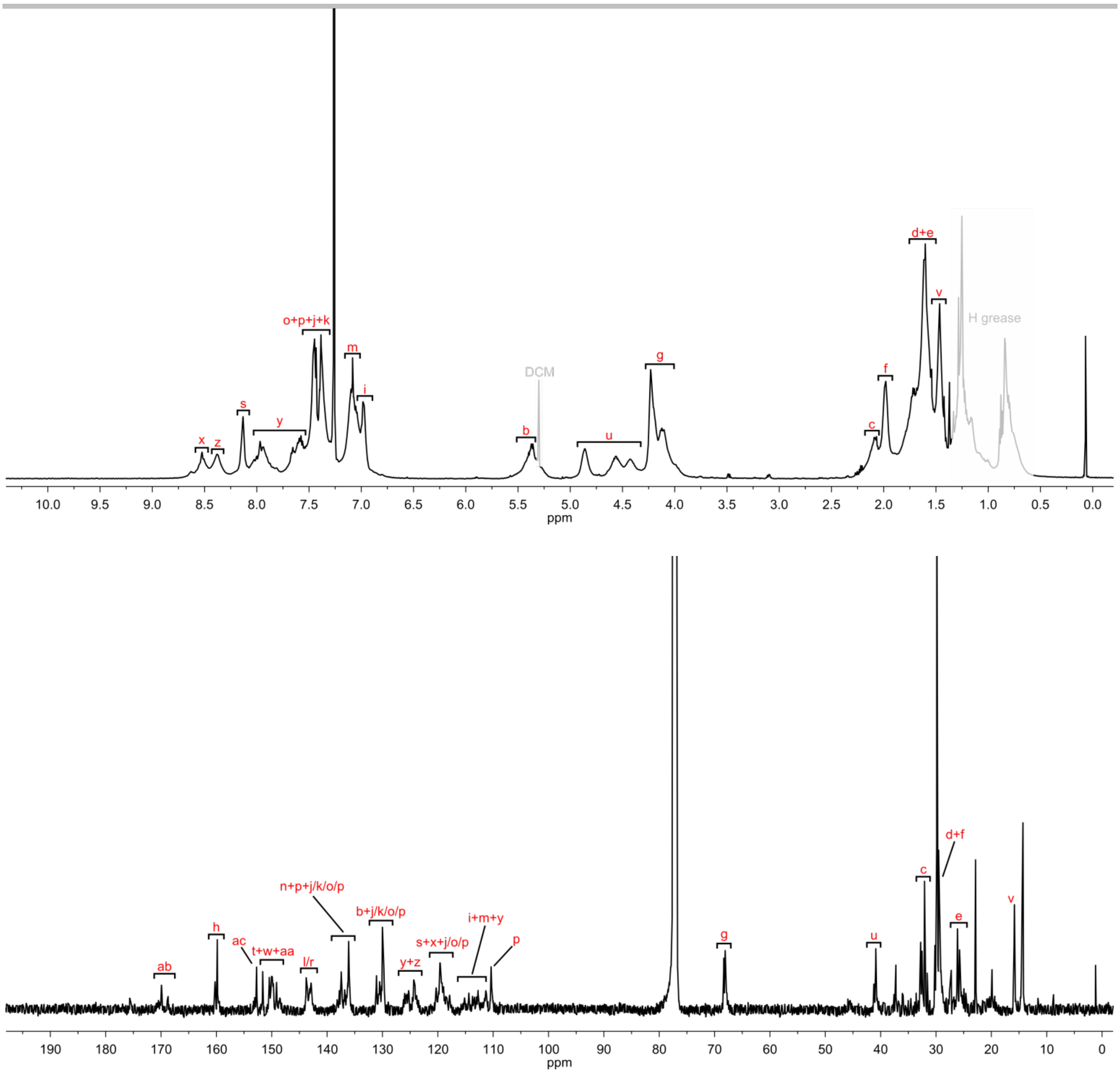

Figure S33. ^{1}H NMR (600 MHz, $CDCl_3$) spectrum (top), ^{13}C NMR (151 MHz, $CDCl_3$) spectrum (bottom) and peak assignment of pure link **2** after GPC separation (Figure S24, blue fraction).

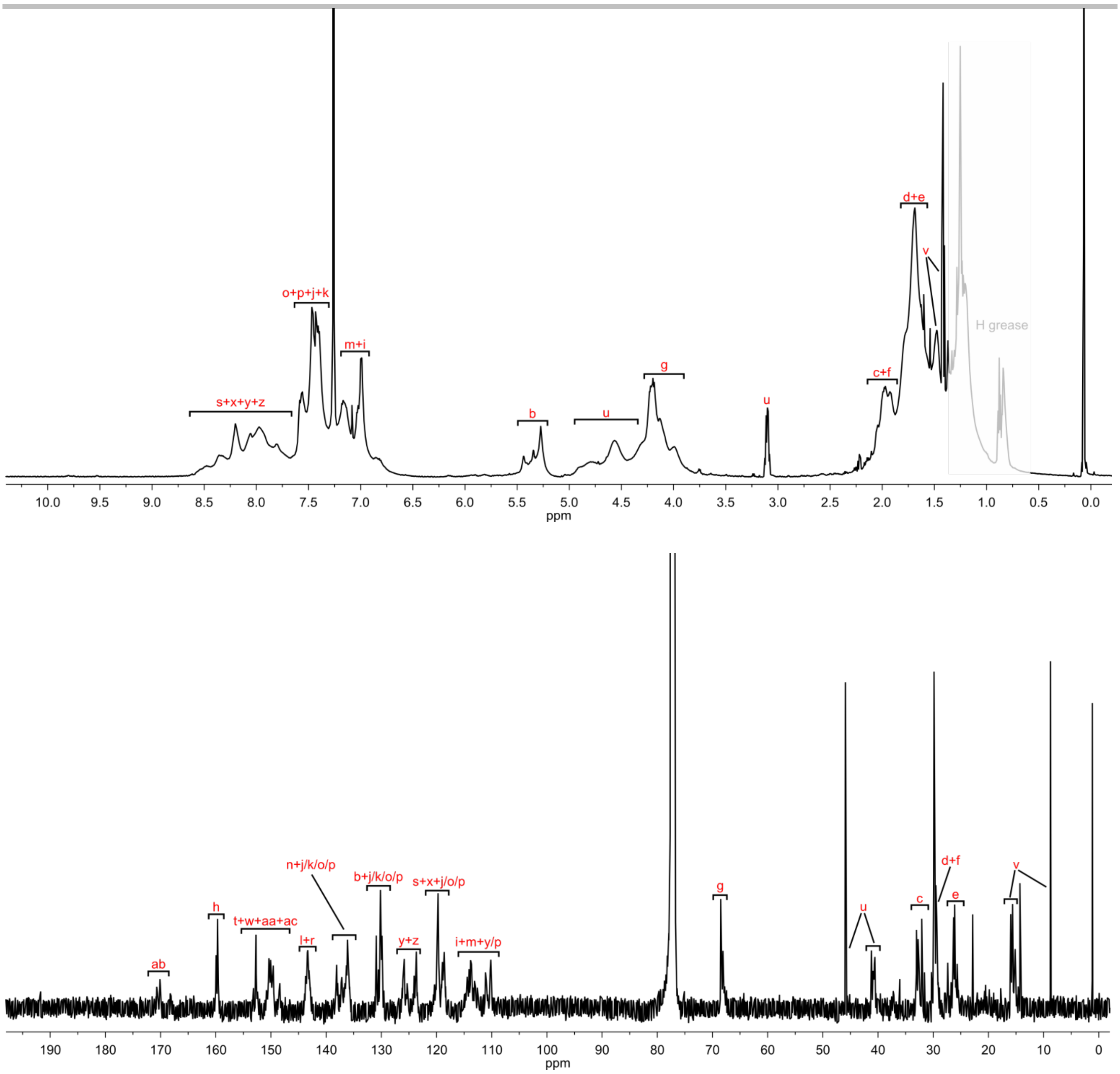

Figure S34. ^{1}H NMR (600 MHz, $CDCl_3$) spectrum (top), ^{13}C NMR (151 MHz, $CDCl_3$) spectrum (bottom) and peak assignment of pure knot **3** after GPC separation (Figure S25, green fraction).

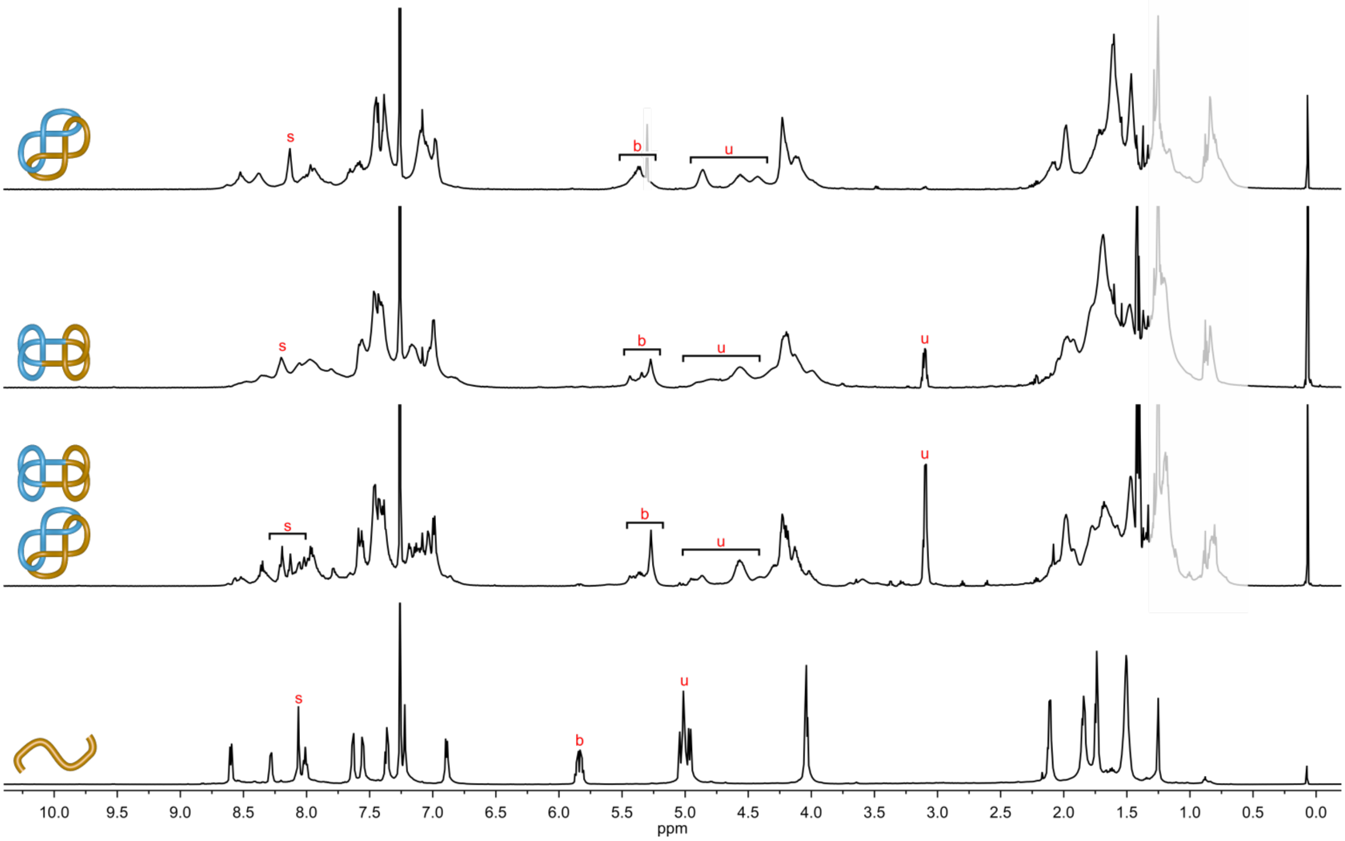

Figure S35. ^{1}H NMR (600 MHz, $CDCl_3$) spectra and representative peaks for (from top to bottom) pure link **2** (Figure S24, blue fraction), pure knot **3** (Figure S25, green fraction), pure **2**/**3** mixture (Figure S5, green fraction) and ligand **1**.

2.4. Remetallation of pure topoisomers 2 and 3

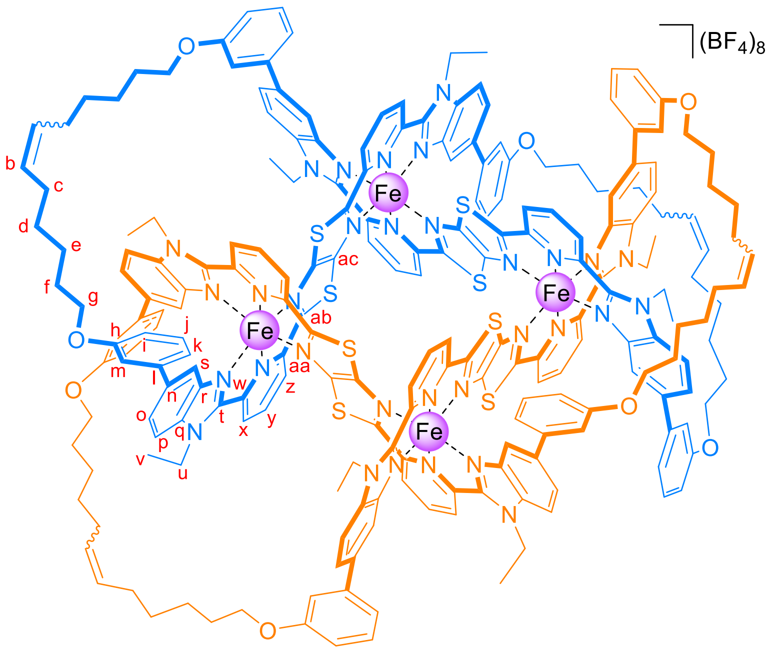

$[Fe_4\mathbf{2}](BF_4)_8$

In an NMR tube **2** (2.0 mg, 0.536 µmol) was dissolved in $CHCl_3$ (0.25 mL) then 360 µL of a 0.0597 M solution of iron(II) tetrafluoroborate hexahydrate (21.4 µmol) in MeCN were added. The mixture turned dark green almost instantaneously and it was heated at 70°C for 16 h. The solvent was then removed under reduced pressure and the solid redissolved in MeCN (0.5 mL). The product was precipitated with a 0.1 M aqueous solution of KBF_4 and filtered over Celite®. After washing with water (30 mL) and Et_2O (5 mL), the product was taken into MeCN. Upon removal of the solvent under reduced pressure, $[Fe_4\mathbf{2}](BF_4)_8$ was obtained as a dark green solid (1.3 mg, 52%).^{1}H NMR (600 MHz, Acetonitrile-*d*$_3$) δ 9.94 – 9.19 (H^x + H^z), 9.16 – 8.96 (H^y), 7.63 – 7.25 (H^j + H^o + H^p), 7.02 – 6.89 (H^k), 6.89 – 6.56 (H^i), 6.50 – 6.35 (H^m), 5.81 – 5.62 (H^b), 5.60 – 5.29 (H^s), 5.13 – 4.55 (H^u), 4.24 – 3.95 (H^g), 2.38 – 2.24 (H^c), 2.04 – 1.58 (H^d + H^e + Hf), 1.36 – 1.13 (H^v). ^{13}C NMR (151 MHz, Acetonitrile-*d*$_3$) δ 160.82 – 160.50 (C^h), 131.76 – 131.48 (C^b), 131.40 – 131.10 (C^j), 130.90 – 130.62 (C^s), 126.83 – 126.24 (C^p or C^o), 120.23 – 119.97 (C^i), 115.20 – 112.63 (C^m + C^k + C^s + C^p or C^o),69.31 – 68.60 (C^g), 33.76 – 33.33 (C^c), 30.74 – 26.18 (C^d + C^e + C^f), 16.69 – 15.68 (C^v) Carbons l, n, q, r, t, u, w, x, y, z, aa, ab, ac could not be unambiguously assigned. LR ESI-MS: *m/z* = 1463.0 $[M–3BF_4]^{3+}$ calculated 1462.7; 1075.6 $[M–4BF_4]^{4+}$ calculated 1075.3; 843.1 $[M–5BF_4]^{5+}$ calculated 842.8; 688.2 $[M–6BF_4]^{6+}$ calculated 687.9; 577.8 $[M–7BF_4]^{7+}$ calculated 577.2.

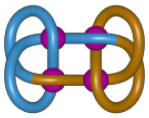

$[Fe_4\mathbf{3}](BF_4)_8$

In an NMR tube **3** (2.3 mg, 0.616 µmol) was dissolved in $CHCl_3$ (0.25 mL) then 211 µL of a 0.0233 M solution of iron(II) tetrafluoroborate hexahydrate (4.93 µmol) in MeCN were added. The mixture turned dark green almost instantaneously and it was stirred at 50°C for 24 h. The solvent was then removed under reduced pressure and the solid redissolved in MeCN (0.5 mL). The product was precipitated with a 0.1 M aqueous solution of KBF_4 and filtered over Celite®. After washing with water (30 mL) and Et_2O (5 mL), the product was taken into MeCN. Upon removal of the solvent under reduced pressure, $[Fe_4\mathbf{3}](BF_4)_8$ was obtained as a dark green solid (1.2 mg, 42%).^{1}H NMR (600 MHz, Acetonitrile-*d*$_3$) δ 9.81 – 9.20 (H^x + H^z), 9.19 – 8.93 (H^y), 7.51 – 7.24 (H^j + H^o + H^p), 7.00 – 6.90 (H^k), 6.88 – 6.62 (H^i), 6.57 – 6.31 (H^m), 5.82 – 5.64 (H^b), 5.84 – 5.17 (H^s), 4.96 – 4.57 (H^u), 4.21 – 4.02 (H^g), 2.46 – 2.24 (H^c), 2.04 – 1.62 (H^d + H^e + Hf), 1.33 – 1.15 (H^v). ^{13}C NMR (151 MHz, Acetonitrile-*d*$_3$) δ 160.76 – 160.40 (C^h), 131.82 – 131.48 (C^b), 131.40 – 131.12 (C^j), 130.86 – 130.63 (C^s), 126.74 – 126.28 (C^p or C^o), 120.19 – 119.91 (C^i), 114.87 – 114.41 (C^m + C^p or C^o), 114.23 – 113.89 (C^k), 113.84 – 113.28 (C^s),69.17 – 68.71 (C^g), 69.17 – 68.71 (C^g), 43.04 – 42.38 (C^u), 33.87 – 33.33 (C^c), 30.84 – 26.32 (C^d + C^e + C^f), 16.58 – 15.83 (C^v) Carbons l, n, q, r, t, w, x, y, z, aa, ab, ac could not be unambiguously assigned. LR ESI-MS: *m/z* = 1462.8 $[M–3BF_4]^{3+}$ calculated 1462.7; 1075.5 $[M–4BF_4]^{4+}$ calculated 1075.3; 843.3 $[M–5BF_4]^{5+}$ calculated 842.8; 688.3 $[M–6BF_4]^{6+}$ calculated 687.9.

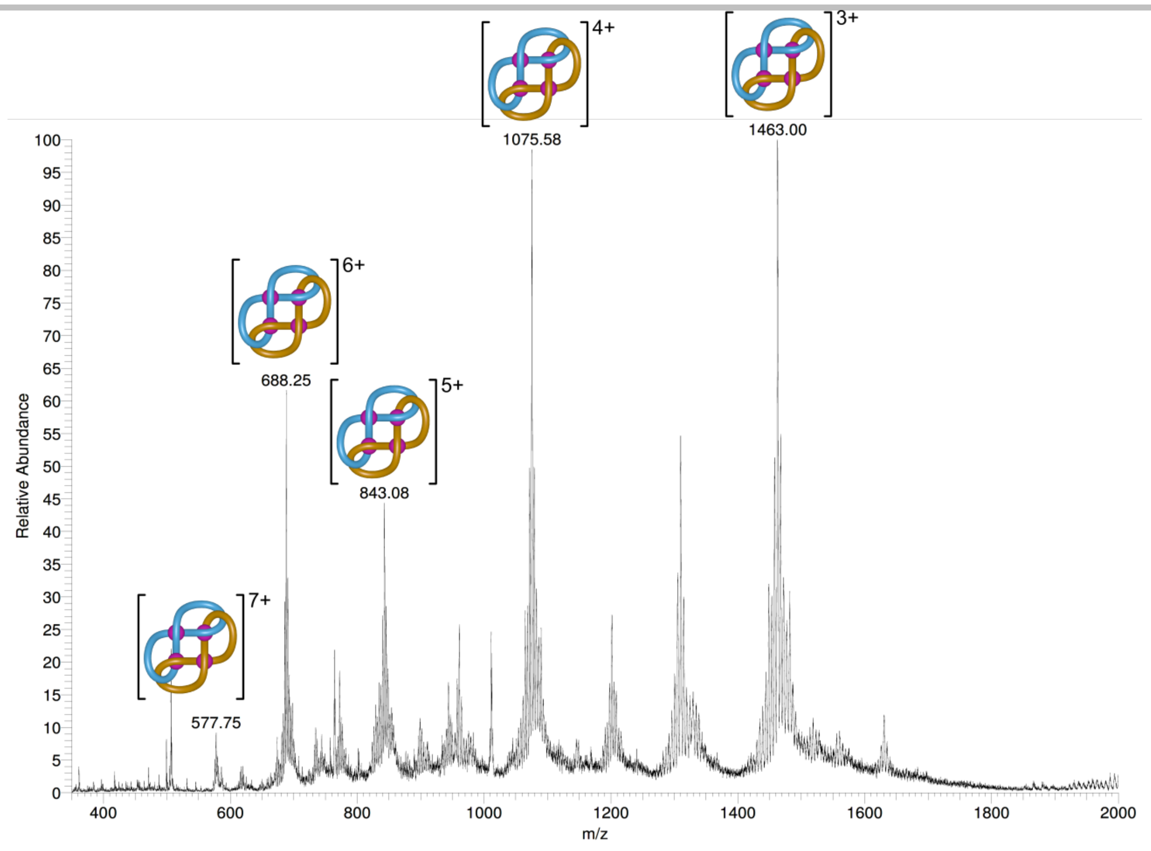

Figure S36. Low-resolution ESI-MS of $[Fe_4\mathbf{2}](BF_4)_8$ after remetallation (all peaks observed as the $[Fe_4\mathbf{2}+(8–n)BF_4]^{n+}$ adducts). Calculated peaks (*m/z*): 1462.7 $[M–3BF_4]^{3+}$; 1075.3 $[M–4BF_4]^{4+}$; 842.8 $[M–5BF_4]^{5+}$; 687.9 $[M–6BF_4]^{6+}$; 577.2 $[M–7BF_4]^{7+}$.

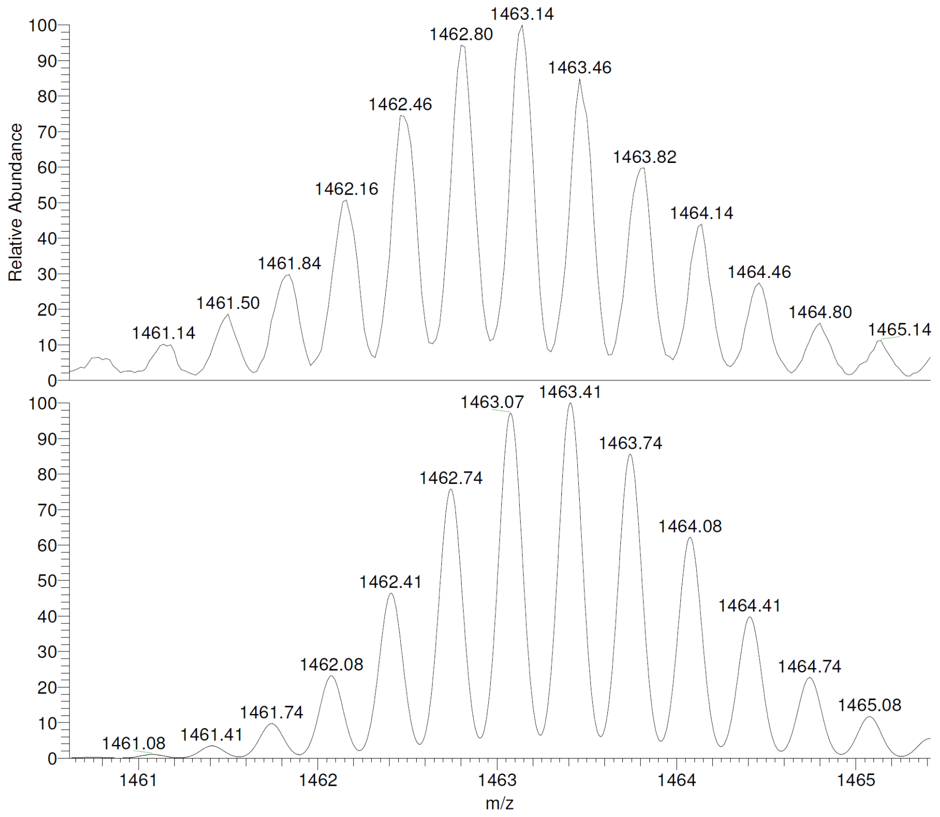

Figure S37. Low resolution ESI-MS of the $[M–3BF_4]^{3+}$ peak from $[Fe_4\mathbf{2}](BF_4)_8$. Experimental spectrum (top) and calculated spectrum (bottom).

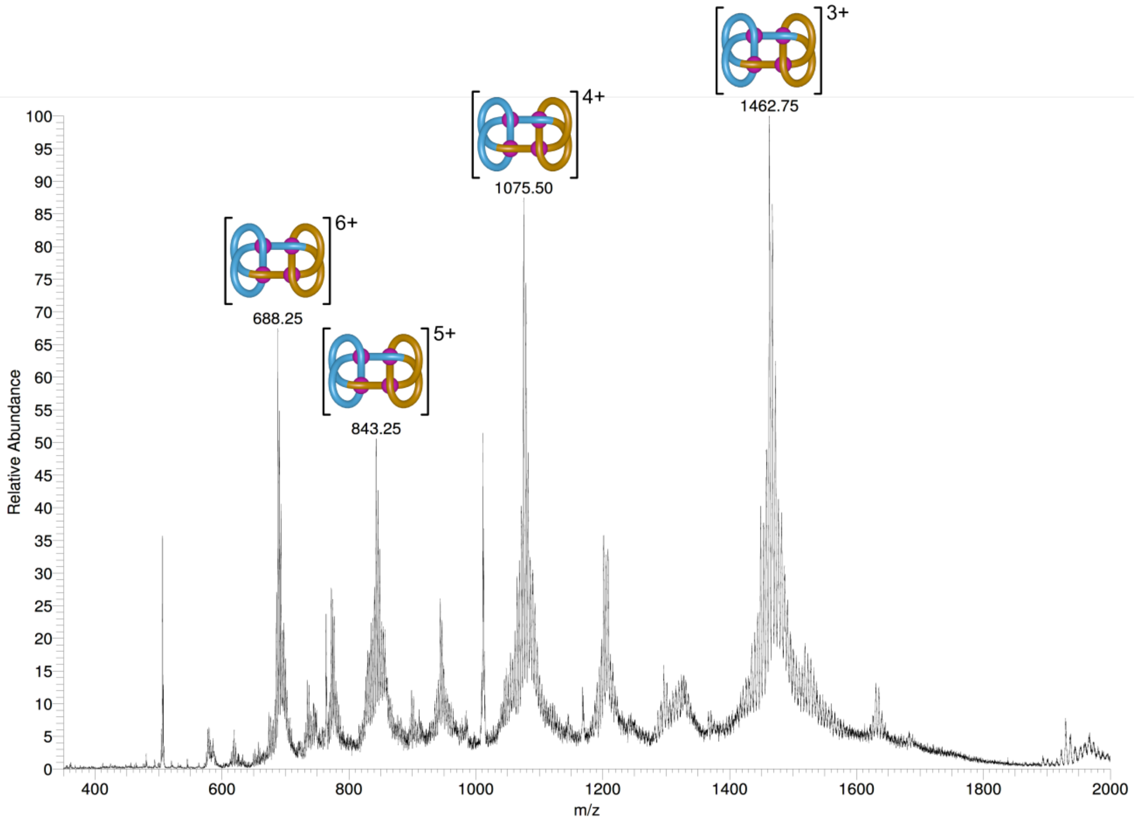

Figure S38. Low-resolution ESI-MS of $[Fe_4\mathbf{3}](BF_4)_8$ after remetallation (all peaks observed as the $[Fe_4\mathbf{3}+(8–n)BF_4]^{n+}$ adducts). Calculated peaks (*m/z*): 1462.7 $[M–3BF_4]^{3+}$; 1075.3 $[M–4BF_4]^{4+}$; 842.8 $[M–5BF_4]^{5+}$; 687.9 $[M–6BF_4]^{6+}$; 577.2 $[M–7BF_4]^{7+}$.

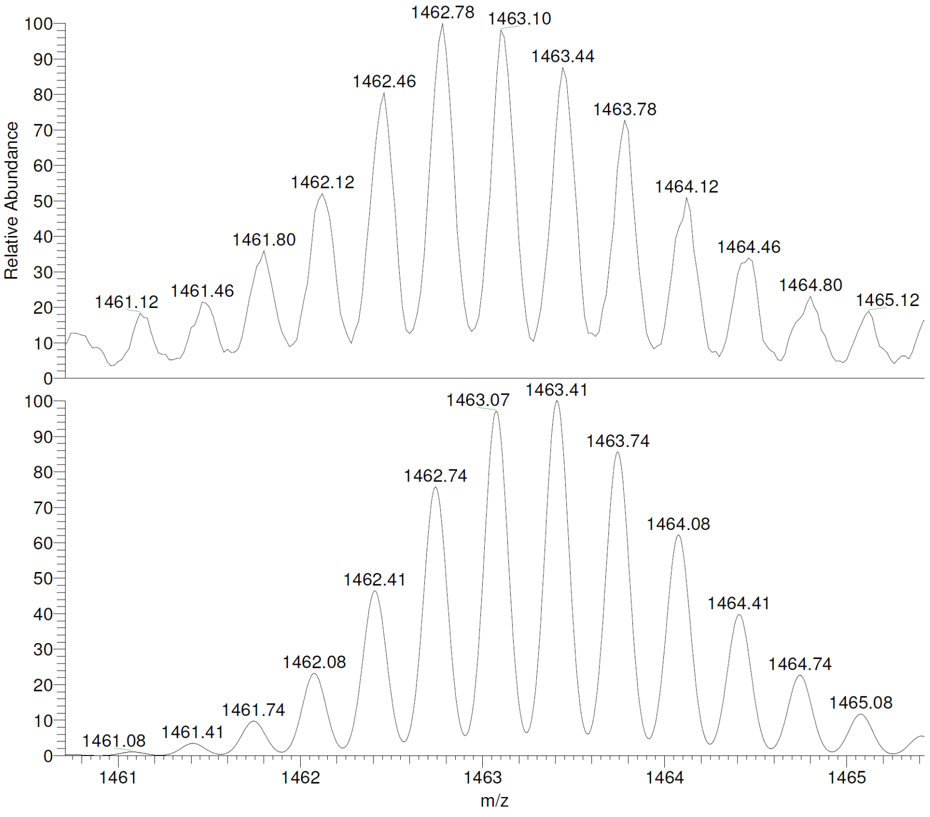

Figure S39. Low resolution ESI-MS of the $[M–3BF_4]^{3+}$ peak from $[Fe_4\mathbf{3}](BF_4)_8$. Experimental spectrum (top) and calculated spectrum (bottom).

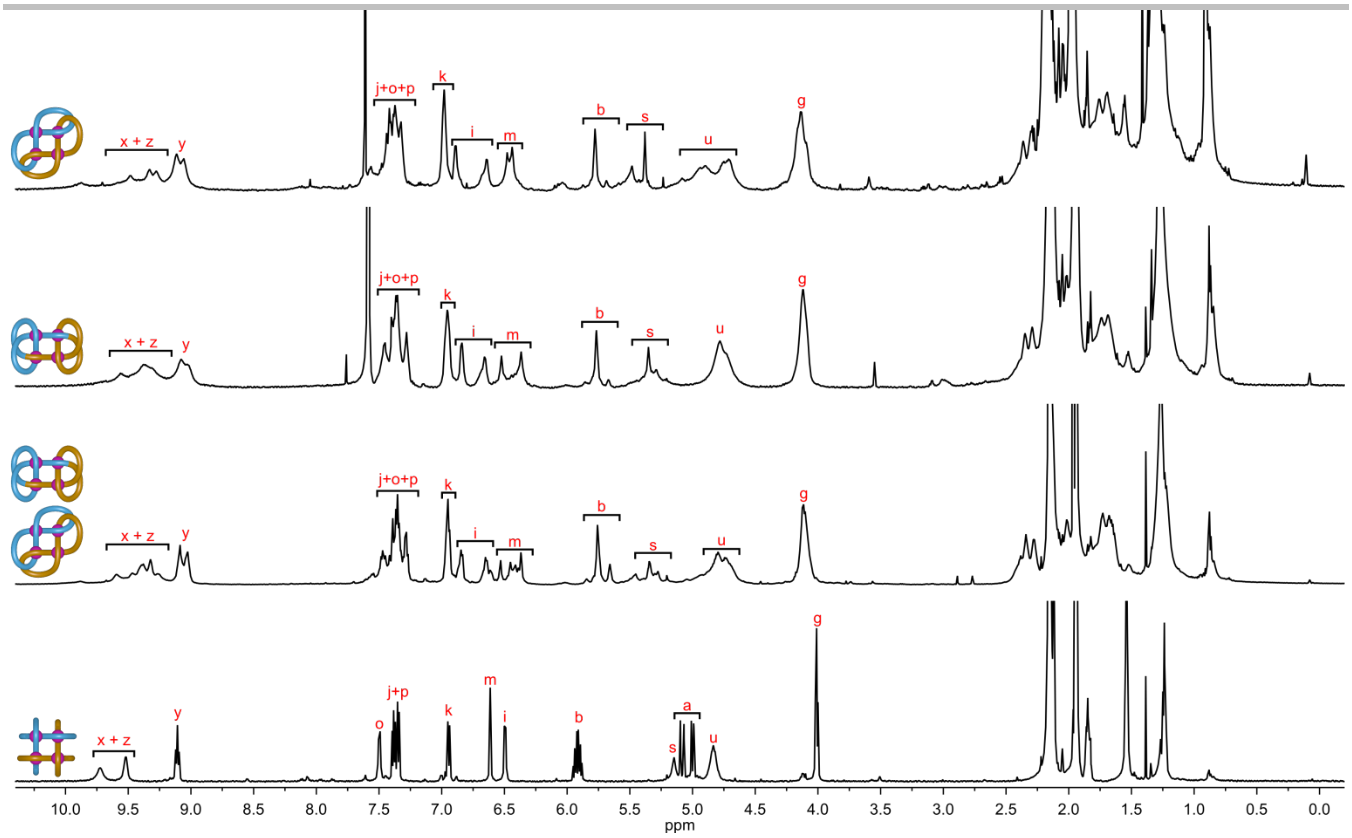

Figure S40. ^{1}H NMR (600 MHz, CD_3CN) spectra and representative peaks for (from top to bottom) remetallated link [Fe_4**2**]$(BF_4)_8$, remetallated knot [Fe_4**3**]$(BF_4)_8$, [Fe_4**2**/**3**]$(BF_4)_8$ remetallated mixture and open 2 × 2 grid [Fe_4**1**$_4$]$(BF_4)_8$.

2.5. Synthesis of the model ligand and model grids

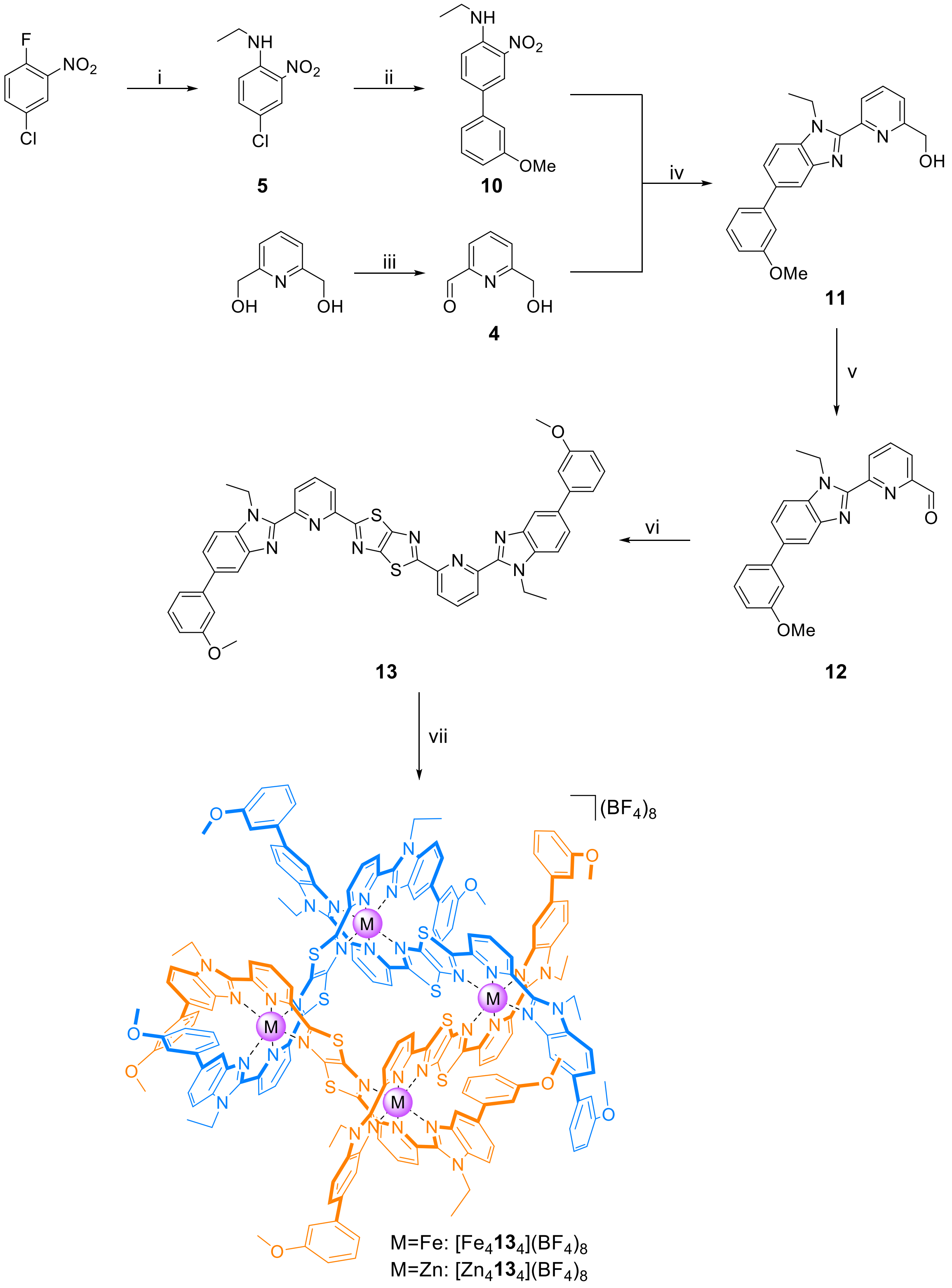

Scheme S3. i) $EtNH_2{\cdot}HCl$, K_2CO_3, DMSO, 90°C, 2h; ii) potassium 3-methoxyphenyltrifluoroborate, $Pd(OAc)_2$, SPhos, K_2CO_3, MeOH, N_2, 65°C, 16h iii) MnO_2, $CHCl_3$, 62°C, 16h; iv) $Na_2S_2O_4$, EtOH/H_2O, N_2, 80°C, 16h; v) MnO_2, $CHCl_3$, 62°C, 16h vi) dithiooxamide, 130°C, DMF, 16h; vii) $M(BF_4)_2$ (M = Fe or Zn), DCM/MeCN, r.t., 15 min.

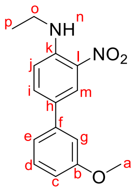

10

5 (114 mg, 0.57 mmol), potassium 3-methoxyphenyltrifluoroborate (182 mg, 0.85 mmol), palladium(II) acetate (1.9 mg, 8.5 µmol), SPhos (7.0 mg, 17 µmol) and potassium carbonate (235 mg , 1.7 mmol) were dissolved in anhydrous and degassed methanol (1.2 mL), and the mixture was heated to reflux for 16 hours with magnetic stirring. After cooling to room temperature, the crude mixture was diluted with ethyl acetate, filtered through silica and eluted with additional eluent. Purification by flash column chromatography (SiO_2, hexane:AcOEt 95:5) yielded **10** as an orange solid (79 mg, 54%). ^{1}H NMR (600 MHz, Chloroform-*d*) δ 8.44 (d, *J* = 2.2 Hz, 1H, H^{m}), 8.04 (br. s, 1H, H^{N}), 7.72 (dd, *J* = 8.9, 2.2 Hz, 1H, H^{i}), 7.35 (t, *J* = 7.9 Hz, 1H, H^{d}), 7.15 (ddd, *J* = 7.6, 1.5, 0.8 Hz, 1H, H^{e}), 7.10 – 7.07 (m, 1H, H^{g}), 6.94 (d, *J* = 8.9 Hz, 1H, H^{j}), 6.89 – 6.86 (m, 1H, H^{c}), 3.87 (s, 3H, H^{a}), 3.41 (qd, *J* = 7.2, 5.3 Hz, 2H, H^{o}), 1.40 (t, *J* = 7.2 Hz, 3H, H^{p}). ^{13}C NMR (151 MHz, Chloroform-*d*) δ 160.24 (C^{b}), 144.91 (C^{k}), 140.55 (C^{f}), 135.21 (C^{i}), 131.98 (C^{l}), 130.11 (C^{d}), 128.30 (C^{h}), 124.79 (C^{m}), 118.86 (C^{e}), 114.45 (C^{j}), 112.61 (C^{c}), 112.15 (C^{g}), 55.51 (C^{a}), 37.97 (C^{o}), 14.58 (C^{p}).LR ESI-MS: *m/z* = 304.2 $[M+MeOH+H]^{+}$ (calcd. for $C_{16}H_{21}N_2O_4$, 305.15).

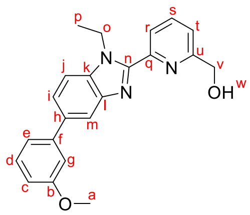

11

6-(hydroxymethyl)picolinaldehyde (44 mg, 0.32 mmol) and **10** (79 mg, 0.29 mmol) were added into a mixture of ethanol (2 mL) and water (0.75 mL). The solution was degassed by bubbling argon and subsequently heated to 80°C until complete dissolution of the solids. Finally, sodium dithionite (152 mg, 0.88 mmol) was added in one portion under positive pressure of nitrogen, yielding a bright yellow suspension. The mixture was then stirred under nitrogen at 80°C for 16 hours. After cooling to room temperature, the solvent was removed under reduced pressure. The residue was partitioned between CH_2Cl_2 and saturated aqueous Na_2CO_3, and, after separation, the aqueous layer was extracted with more CH_2Cl_2. The combined organic layers were then washed with brine before drying over $MgSO_4$ and removal of the solvent under reduced pressure. Flash column chromatography (SiO_2, DCM:MeOH 100:0 to 98:2) yielded **11** as a yellow solid (75 mg, 72%). ^{1}H NMR (600 MHz, Chloroform-*d*) δ 8.32 (d, *J* = 7.7 Hz, 1H, H^{r}), 8.05 (d, *J* = 1.1 Hz, 1H, H^{m}), 7.87 (t, *J* = 7.8 Hz, 1H, H^{s}), 7.61 (dd, *J* = 8.4, 1.6 Hz, 1H, H^{i}), 7.51 (d, *J* = 8.4 Hz, 1H, H^{j}), 7.41 – 7.34 (m, 2H, H^{d} + H^{t}), 7.29 – 7.26 (m, 1H, H^{e}), 7.23 – 7.21 (m, 1H, H^{g}), 6.92 – 6.88 (m, 1H, H^{c}), 4.89 (s, 2H, H^{v}), 4.80 (q, *J* = 7.2 Hz, 2H, H^{o}), 3.89 (s, 3H, H^{a}), 1.58 (t, *J* = 7.2 Hz, 3H, H^{p}). ^{13}C NMR (151 MHz, Chloroform-*d*) δ 160.07 (C^{b}), 158.75(C^{u}), 150.19 (C^{n}), 149.44 (C^{q}), 143.39 (C^{f}), 143.28 (C^{l}), 137.84 (C^{s}), 136.42 (C^{h}), 135.93 (C^{k}), 129.92 (C^{d}), 123.70 (C^{r}), 123.50 (C^{i}), 120.75 (C^{t}), 120.05 (C^{e}), 118.65 (C^{m}), 113.20 (C^{g}), 112.49 (C^{c}), 110.24 (C^{j}), 64.59 (C^{v}), 55.45 (C^{a}), 40.92 (C^{o}), 15.62 (C^{p}). LR ESI-MS: *m/z* = 360.4 $[M+H]^{+}$ (calcd. for $C_{22}H_{22}N_3O_2$, 360.17).

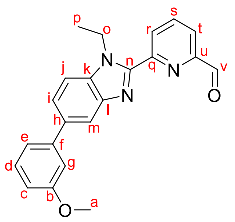

12

To a solution of **11** (75 mg, 0.21 mmol) in chloroform (5 mL) MnO_2 was added (183 mg, 2.1 mmol) and the suspension was stirred at reflux for 16 hours. Upon cooling, the mixture was filtered over Celite® and eluted with dichloromethane/methanol 98:2. The solvent was removed under reduced pressure yielding product **12** as a yellow solid (65 mg, 87%), which was deemed sufficiently pure to use in the following step without further purification. ^{1}H NMR (600 MHz, Chloroform-*d*) δ 10.16 (s, 1H, H^{v}), 8.71 (dd, *J* = 7.5, 1.4 Hz, 1H, H^{r}), 8.12 – 8.00 (m, 3H, H^{m} + H^{s} + H^{t}), 7.64 (dd, *J* = 8.4, 1.5 Hz, 1H, H^{i}), 7.55 (d, *J* = 8.4 Hz, 1H, H^{j}), 7.39 (t, *J* = 7.9 Hz, 1H, H^{d}), 7.28 (d, *J* = 7.7 Hz, 1H, H^{e}), 7.24 – 7.20 (m, 1H, H^{g}), 6.91 (dd, *J* = 8.1, 2.1 Hz, 1H, H^{c}), 4.96 (q, *J* = 7.1 Hz, 2H, H^{o}), 3.89 (s, 3H, H^{a}), 1.63 (t, *J* = 7.1 Hz, 3H, H^{p}). ^{13}C NMR (151 MHz, Chloroform-*d*) δ 192.86 (C^{v}), 160.10 (C^{b}), 152.10 (C^{u}), 151.24 (C^{q}), 149.11 (C^{n}), 143.31 (2C,

$C^f + C^l$), 138.20 (C^s), 136.61 (C^h), 136.18 (C^k), 129.95 (C^d), 128.87 (C^r), 123.87 (C^i), 121.67 (C^t), 120.04 (C^e), 118.79 (C^m), 113.21 (C^g), 112.54 (C^c), 110.36 (C^j), 55.45 (C^a), 41.21 (C^o), 15.69 (C^p). LR ESI-MS: *m/z* = 358.4 $[M+H]^+$ (calcd. for $C_{22}H_{20}N_3O_2$, 358.16).

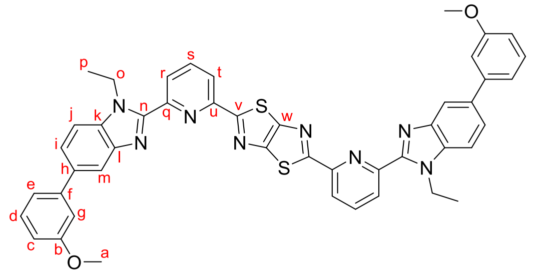

13

A solution of **12** (65 mg, 0.18 mmol) and dithiooxamide (11 mg, 0.091 mmol) in DMF (2 mL) was heated at 130°C for 17 hours. After cooling, precipitation via addition of methanol (40 mL) followed by vacuum filtration and wash with excess methanol afforded **13** (37 mg, 51%) as a yellow powder, which required no further purification ^{1}H NMR (600 MHz,trifluoroacetic acid-*d*) δ 8.55 (d, *J* = 7.9 Hz, 2H, H^r), 8.34 (t, *J* = 7.9 Hz, 2H, H^s), 8.29 (d, *J* = 7.8 Hz, 2H, H^t), 8.02 (s, 2H, H^m), 7.98 (d, *J* = 9.1 Hz, 2H, H^i), 7.91 (d, *J* = 8.7 Hz, 2H, H^j), 7.46 – 7.39 (m, 2H, H^d), 7.34 – 7.29 (m, 4H, $H^e + H^g$), 7.06 (dd, *J* = 8.2, 1.8 Hz, 2H, H^c), 5.03 (q, *J* = 7.0 Hz, 4H, H^o), 3.99 (s, 6H, H^a), 1.88 (t, *J* = 7.3 Hz, 6H, H^p). ^{13}C NMR (151 MHz, trifluoroacetic acid-*d*) δ 174.18 (C^v), 160.44 (C^b), 154.28 (C^w), 153.15 (C^u or C^q), 146.79 (C^n), 145.07 (C^h), 144.26 (C^u or C^q), 142.94 (C^f), 142.86 (C^s), 134.23 (C^k), 132.76 (C^l), 132.73 (C^d), 130.27 (C^i), 129.32 (C^t), 126.18 (C^r), 123.96 (C^e), 116.32 (C^g), 116.21 (C^c), 114.86 (C^j), 114.57 (C^m), 57.78 (C^a), 44.91 (C^o), 15.90 (C^p).

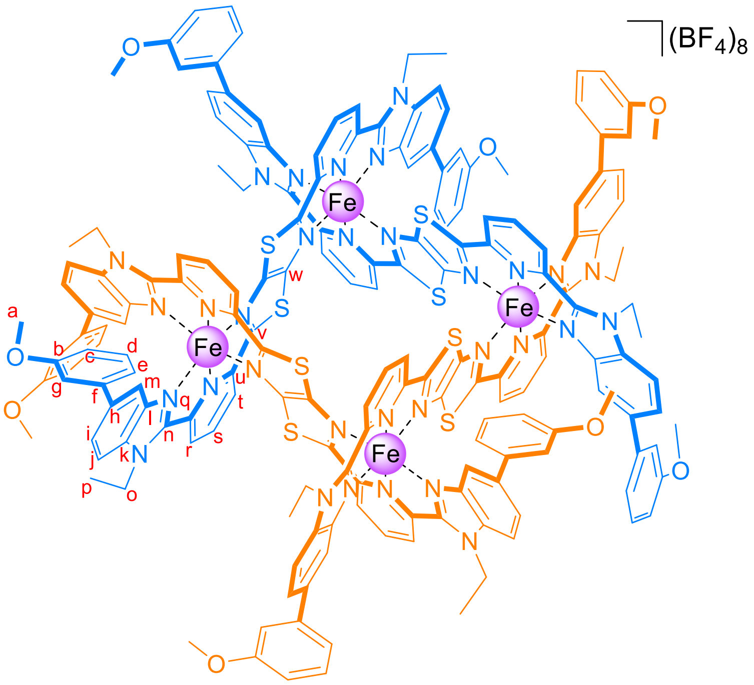

$[Fe_4\mathbf{13}_4](BF_4)_8$

To a solution of **13** (5.0 mg, 6.3 μmol) in CH_2Cl_2 (0.4 mL) at room temperature was added a solution of iron(II) tetrafluoroborate hexahydrate (2.1 mg, 6.3 μmol) in acetonitrile (0.4 mL). Within a minute the brownish yellow solution had become an intensely dark green solution. The mixture was then stirred for further 15 minutes, after which the solution was filtered through a syringe PTFE filter. The green product $[Fe_4\mathbf{13}_4](BF_4)_8$ was characterised in solution without isolation (6.4 mg, quant. yield). Crystals were obtained by slow diffusion of diisopropyl ether into the CH_2Cl_2/MeCN solution of the complex. ^{1}H NMR (600 MHz, Acetonitrile-*d*$_3$) δ 9.80 (br. s, 8H, H^t or H^r), 9.54 (br. s, 8H, H^t or H^r), 9.09 (t, *J* = 8.1 Hz, 8H, H^s), 7.48 (d, *J* = 7.7 Hz, 8H, H^i), 7.34 (t, *J* = 7.6 Hz, 8H, H^d), 7.28 (d, *J* = 7.9 Hz, 8H, H^j), 6.91 (d, *J* = 8.1 Hz, 8H, H^e), 6.54 (s, 8H, H^g), 6.48 (d, *J* = 6.6 Hz, 8H, H^c), 5.04 (br. s, 8H, H^m), 4.83 (br. s, 16H, H^o), 3.81 (s, 24H, H^a), 1.20 (t, *J* = 7.0 Hz, 24H, H^p). ^{13}C NMR (151 MHz, Acetonitrile-*d*$_3$) δ 161.13 (C^b), 150.52 (2C, $C^q + C^u$), 143.55 (C^s), 141.48 (C^f or C^h), 139.01 (C^f or C^h), 131.18 (C^d), 126.65 (C^j), 120.19 (C^s)c, 115.02 (C^i), 114.13 (C^e), 113.85 (C^m), 113.57 (C^g), 56.22 (C^a), 42.98 (C^o), 16.40 (C^p). Carbons k, l, n, r, t, v and w were not observed.

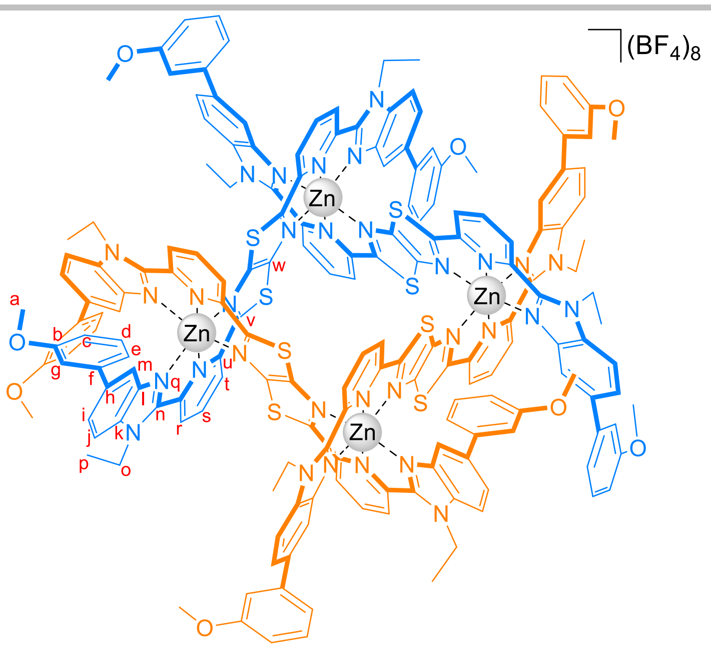

$[Zn_4\mathbf{13}_4](BF_4)_8$

To a solution of **13** (5.0 mg, 6.3 μmol) in CH_2Cl_2 (0.4 mL) at room temperature was added a solution of zinc tetrafluoroborate hydrate (2.2 mg, 6.3 μmol) in acetonitrile (0.4 mL). Within a minute the brownish yellow solution had become an intensely yellow solution. The mixture was then stirred for further 15 minutes, after which the solution was filtered through a syringe PTFE filter. The yellow product $[Zn_4\mathbf{13}_4](BF_4)_8$ was characterised in solution without isolation (6.5 mg, quant. yield). Crystals were obtained by slow diffusion of diisopropyl ether into the CH_2Cl_2/MeCN solution of the complex. ^{1}H NMR (600 MHz, Acetonitrile-d_3) δ 8.91 (t, J = 8.2 Hz, 8H, H^s), 8.80 (d, J = 8.3 Hz, 8H, H^r), 8.48 (d, J = 8.0 Hz, 8H, H^t), 7.54 (d, J = 8.3 Hz, 8H, H^j), 7.49 (d, J = 8.3 Hz, 8H, H^i), 7.23 (t, J = 7.8 Hz, 8H, H^d), 6.87 (dd, J = 8.3, 1.9 Hz, 8H, H^c), 6.72 (d, J = 7.4 Hz, 8H, H^e), 6.69 (s, 8H, H^g), 6.64 (s, 8H, H^m), 4.73 (q, J = 7.3 Hz, 16H, H^o), 3.74 (s, 24H, H^a), 1.49 (t, J = 7.3 Hz, 24H, H^p). ^{13}C NMR (151 MHz, Acetonitrile-d_3) δ 166.96 (C^v), 160.92 (C^b), 151.56 (C^l or C^w), 147.84 (C^s), 147.27 (C^n), 146.23 (C^u), 143.95 (C^q), 141.77 (C^f), 139.57 (C^h), 138.57 (C^l or C^w), 137.12 (C^k), 130.90 (C^d), 127.66 (C^r), 127.04 (C^i), 126.37 (C^t), 120.29 (C^e), 116.06 (C^m), 114.08 (C^c), 113.63 (C^g), 113.28 (C^j), 56.02 (C^a), 42.53 (C^o), 15.26 (C^p).

3. X-ray crystal structures

Data Collection. X-Ray data for compounds [Fe_4**13**$_4$](BF_4)$_8$ and [Zn_4**13**$_4$](BF_4)$_8$ were collected at a temperature of 100 K using a microfocused Bruker X8 Prospector diffractometer with Cu-kα (1.54178) equipped with a CCD detector and an Oxford Cryosystems nitrogen flow gas system. Data were measured using Bruker Apex2 suite of programs. X-Ray data for compound **2** were collected at a temperature of 100 K using a synchrotron radiation at single crystal X-ray diffraction beamline I19 in Diamond light Source,[S2] equipped with an Pilatus 2M detector and an Oxford Cryosystems nitrogen flow gas system. Data were measured using GDA suite of programs.

Crystal structure determinations and refinements. X-Ray data were processed and reduced using CrysAlisPro suite of programmes. Absorption correction was performed using empirical methods (SCALE3 ABSPACK) based upon symmetry-equivalent reflections combined with measurements at different azimuthal angles.[S3] The crystal structure was solved and refined against all F^2 values using the SHELXL and Olex 2 suite of programmes.[S4] Crystals of [Fe_4**13**$_4$](BF_4)$_8$ and **2** present a diffraction limit of 1.1 and 1.0 Å, while crystal of [Zn_4**13**$_4$](BF_4)$_8$ diffracted to 0.83 Å of resolution. All atoms were refined anisotropically except solvent molecules, BF_4 anions and disordered moieties, which were refined isotropically. Hydrogen atoms were placed in the calculated positions. The aromatic moieties were heavily disordered and modelled over two positions were possible. The C-N, C-C and C-O distances in the disordered moieties were restrained using DFIX and SADI and constrained using AFIX SHELXL commands. No restrains were applied to force the configuration of the double bonds of the chains. The atomic displacement parameters (adp) have been restrained using RIGU and SIMU SHELXL commands. A BF_4 anions were found disordered and modelled over two positions where possible. The B-F distances and F-B-F angles were restrained using DFIX SHELXL commands. The adps were also restrained using SIMU and RIGU SHELXL commands.

Compounds [Fe_4**13**$_4$](BF_4)$_8$ and [Zn_4**13**$_4$](BF_4)$_8$ presents large voids filled with a lot of scattered electron density, the solvent mask protocol inside Olex 2 software was used to account for the void electron density corresponding to the disordered solvent molecules placed in the intermolecular space in the crystal structure. Crystals of [Zn_4**13**$_4$](BF_4)$_8$ present 309.3 electrons that were accounted for in a volume of 1352 Å^3. There are 4 grid molecules per unit cell, so there were 77 electrons uncounted per knot, which may correspond to one molecule of disordered BF_4 anions and 1.5 disordered molecules of acetonitrile. Crystals of [Fe_4**13**$_4$](BF_4)$_8$ present 2806.3 electrons that were accounted for in a volume of 8979 Å^3. There are 8 grid molecules per unit cell, so there were 701 electrons uncounted per knot, which may correspond to one molecule of disordered BF_4 anions and 30 disordered molecules of acetonitrile.

A number of A alerts were found in crystals of [Fe_4**13**$_4$](BF_4)$_8$ and **2** due to the poor resolution data obtained (1.1 and 1.0 Å). This resolution is common in big molecules with large intermolecular spaces filled with disordered anions and/or solvent molecules. In order to refine the crystal structure, different moieties were heavily restrained (using DIFX and SADI commands) and constrained (using AFIX commands) to have idealized geometries. Also A-alerts were found in compounds [Fe_4**13**$_4$](BF_4)$_8$, [Zn_4**13**$_4$](BF_4)$_8$ and **2** due to the isotropic refinement of the disordered atoms. Disorder alkyl chains in crystal structure **2** were unsolved in order to maximize the data/parameter ratio.

CCDC 1849964-1849966 contains the supplementary crystallographic data for this paper. These data can be obtained free of charge *via* www.ccdc.cam.ac.uk/conts/retrieving.html (or from the Cambridge Crystallographic Data Centre, 12 Union Road, Cambridge CB21EZ, UK; fax: (+44)1223-336-033; or deposit@ccdc.cam.ac.uk).

Table S1. Crystallographic information for **2**, $[Zn_4\mathbf{13}_4](BF_4)_8$ and $[Fe_4\mathbf{13}_4](BF_4)_8$.

Identification code	**2**	$[Zn_4\mathbf{13}_4](BF_4)_8$	$[Fe_4\mathbf{13}_4](BF_4)_8$
Empirical formula	$C_{265}H_{260}N_{32}O_{11}S_8$	$C_{190}H_{144}B_7F_{28}N_{35}O_8S_8Zn_4$	$C_{193.75}H_{144}B_{7.5}F_{30}Fe_4N_{35.5}O_{8.5}S_8$
Formula weight	4325.51	4171.02	4236.39
Temperature/K	100	150	150
Crystal system	orthorhombic	triclinic	monoclinic
Space group	$Pna2_1$	P-1	$P2_1/c$
a/Å	20.3675(3)	14.1164(7)	16.8345(9)
b/Å	69.0889(17)	23.3888(10)	86.2491(17)
c/Å	15.9645(3)	33.9163(10)	38.832(2)
α/°	90	79.703(3)	90
β/°	90	81.252(4)	127.210(8)
γ/°	90	76.262(4)	90
Volume/Å^3	22464.7(8)	10631.8(8)	44905(5)
Z	4	2	8
ρ_{calc}g/cm^3	1.279	1.303	1.253
μ/mm^{-1}	0.140	1.972	3.443
F(000)	9160.0	4256.0	17300.0
Crystal size/mm^3	0.08 × 0.02 × 0.02	0.35 × 0.1 × 0.1	0.2 × 0.15 × 0.03
Radiation	(λ = 0.6889)	CuKα (λ = 1.54178)	CuKα (λ = 1.54184)
2Θ range for data collection/°	2.996 to 40.296	4.41 to 136.492	3.516 to 89.066
Index ranges	-20 ≤ h ≤ 20, -69 ≤ k ≤ 69, -15 ≤ l ≤ 15	-16 ≤ h ≤ 16, -28 ≤ k ≤ 27, -40 ≤ l ≤ 40	-15 ≤ h ≤ 15, -78 ≤ k ≤ 71, -35 ≤ l ≤ 30
Reflections collected	110501	79647	126462
Independent reflections	23068 [R_{int} = 0.1249, R_{sigma} = 0.0939]	37242 [R_{int} = 0.0885, R_{sigma} = 0.0941]	35251 [R_{int} = 0.1105, R_{sigma} = 0.0868]
Data/restraints/parameters	23068/228/2088	37242/989/2353	35251/2714/4260
Goodness-of-fit on F^2	1.031	1.566	2.171
Final R indexes [I>=2σ (I)]	R_1 = 0.1020, wR_2 = 0.2685	R_1 = 0.1250, wR_2 = 0.3050	R_1 = 0.1523, wR_2 = 0.3635
Final R indexes [all data]	R_1 = 0.1577, wR_2 = 0.3183	R_1 = 0.1905, wR_2 = 0.3400	R_1 = 0.1931, wR_2 = 0.3776
Largest diff. peak/hole / e Å^{-3}	0.41/-0.33	1.15/-0.69	1.60/-0.76
Flack parameter	0.01(7)		

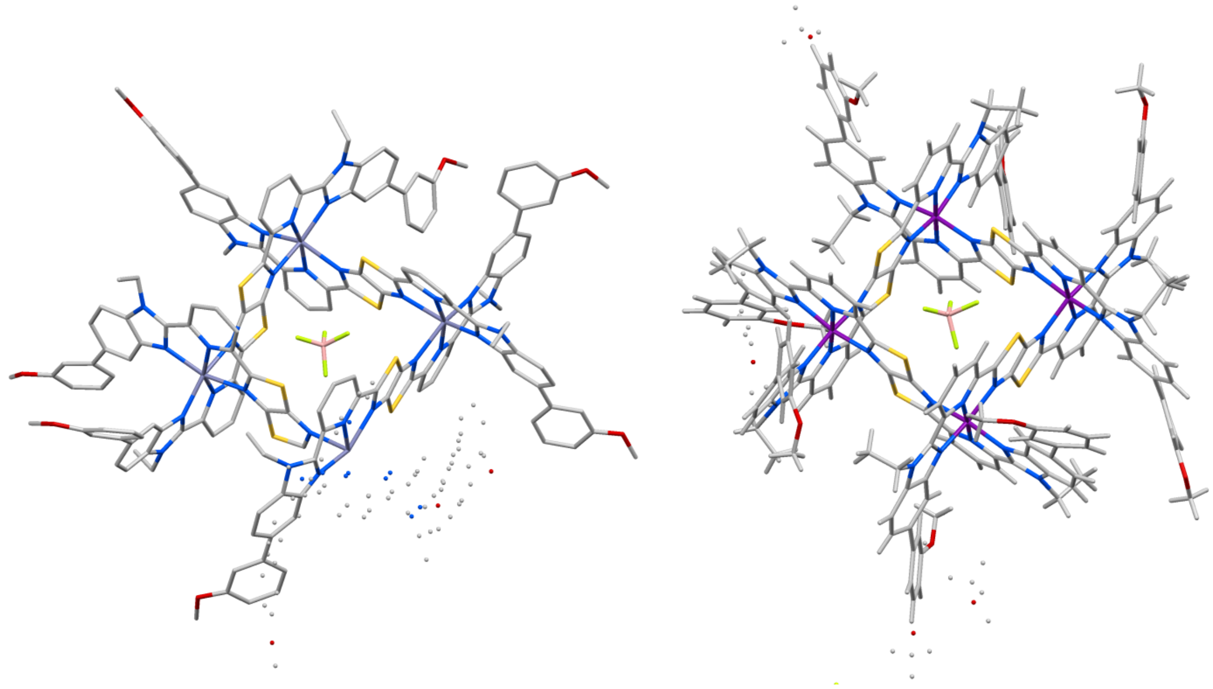

Figure S41. X-ray crystal structure of $[Zn_4\mathbf{13}_4](BF_4)_8$ (left) and $[Fe_4\mathbf{13}_4](BF_4)_8$ (right), showing the formation of the expected interwoven 2 × 2 grids. Disorder is observed around some of the pendant phenyl groups. Both structures contain a BF_4^- anion located in the central cavity of the grid, a behaviour previously observed for this type of system.[S5] Solvent molecules and all other anions have been ommited for clarity. C, grey; N, blue; O, red; S, yellow; B, pink; F, green; Zn, grey-blue; Fe, purple.

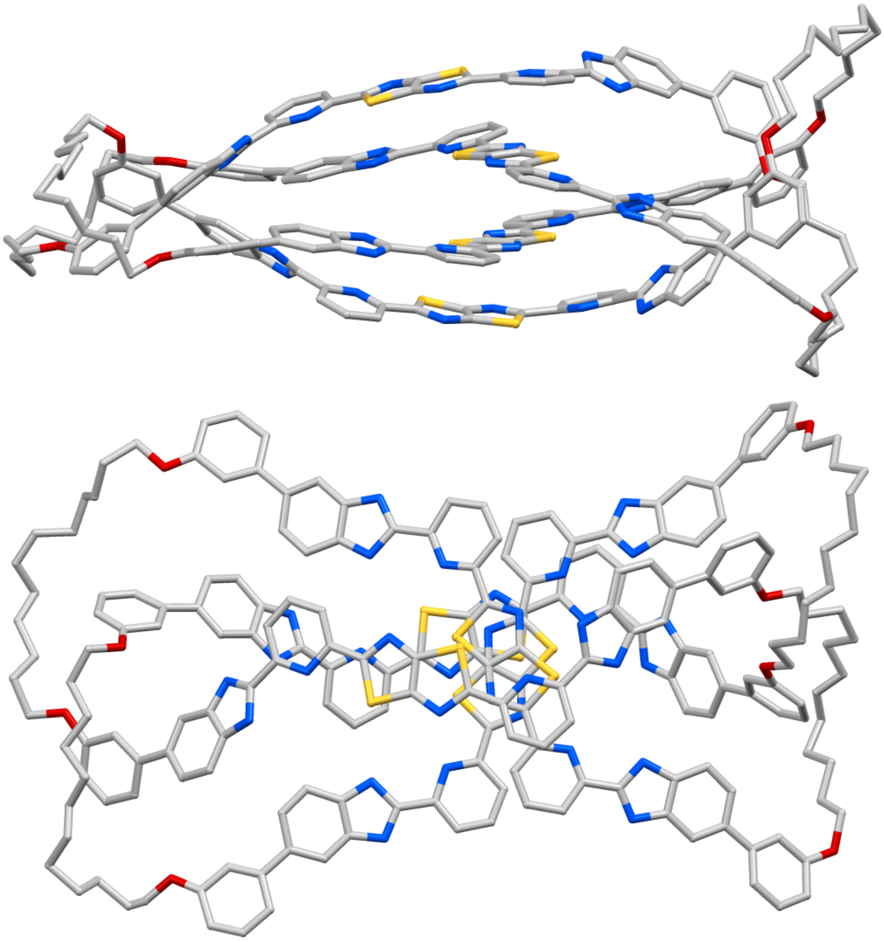

Figure S42. X-ray crystal structure of 6_3^2 link **2**, showing the stacking of the aromatic regions of the ligand strands and the interlocked architecture of the link topology. Solvent molecules and all the pendant ethyl groups have been ommited for clarity. C, grey; N, blue; O, red; S, yellow.

4. NMR spectra

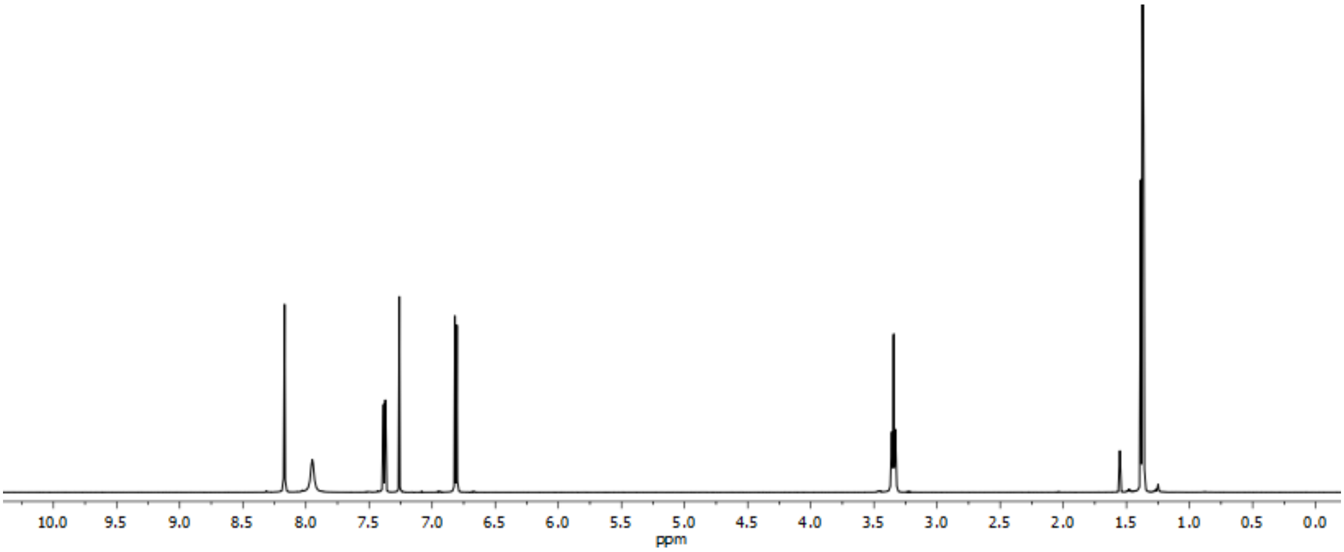

Figure S43. ^{1}H NMR (600 MHz, $CDCl_3$) spectrum of compound **5**.

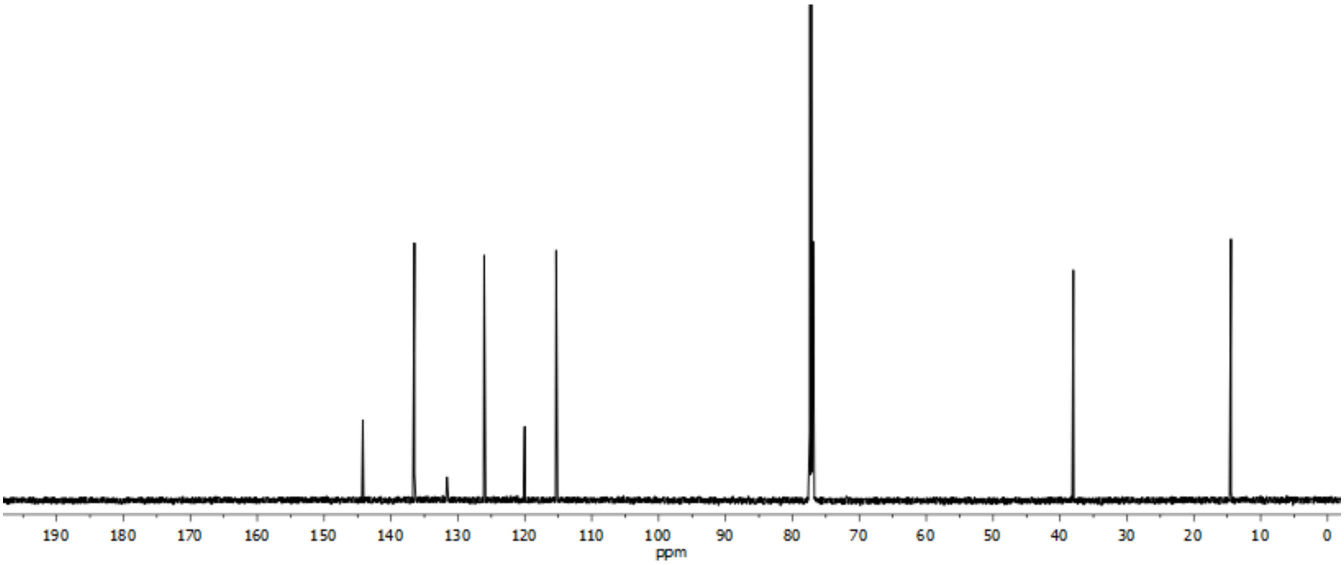

Figure S44. ^{13}C NMR (151 MHz, $CDCl_3$) spectrum of compound **5**.

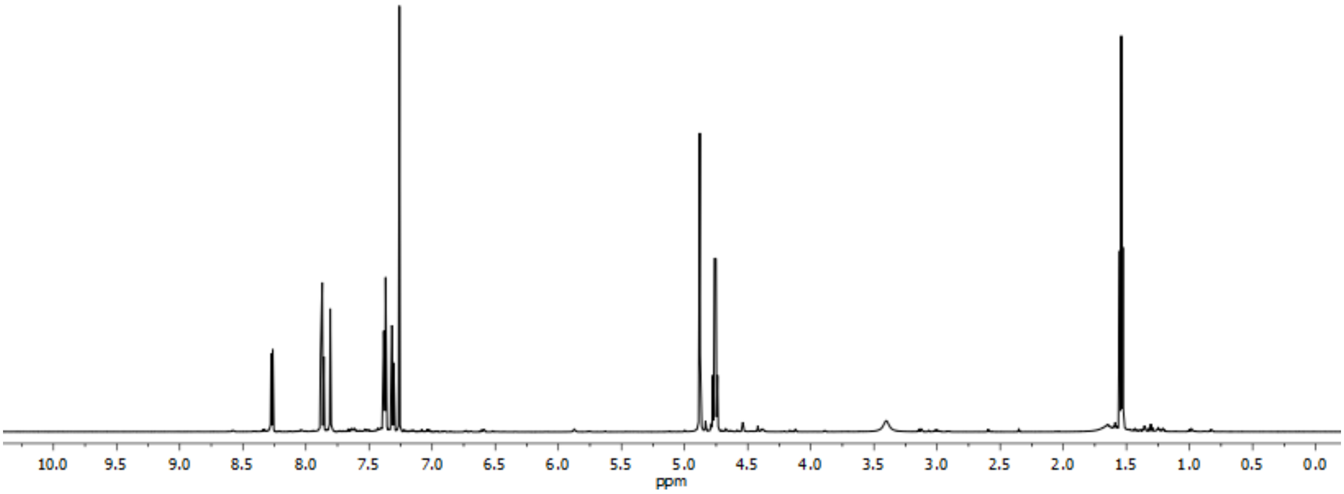

Figure S45. ^{1}H NMR (600 MHz, $CDCl_3$) spectrum of compound **6**.

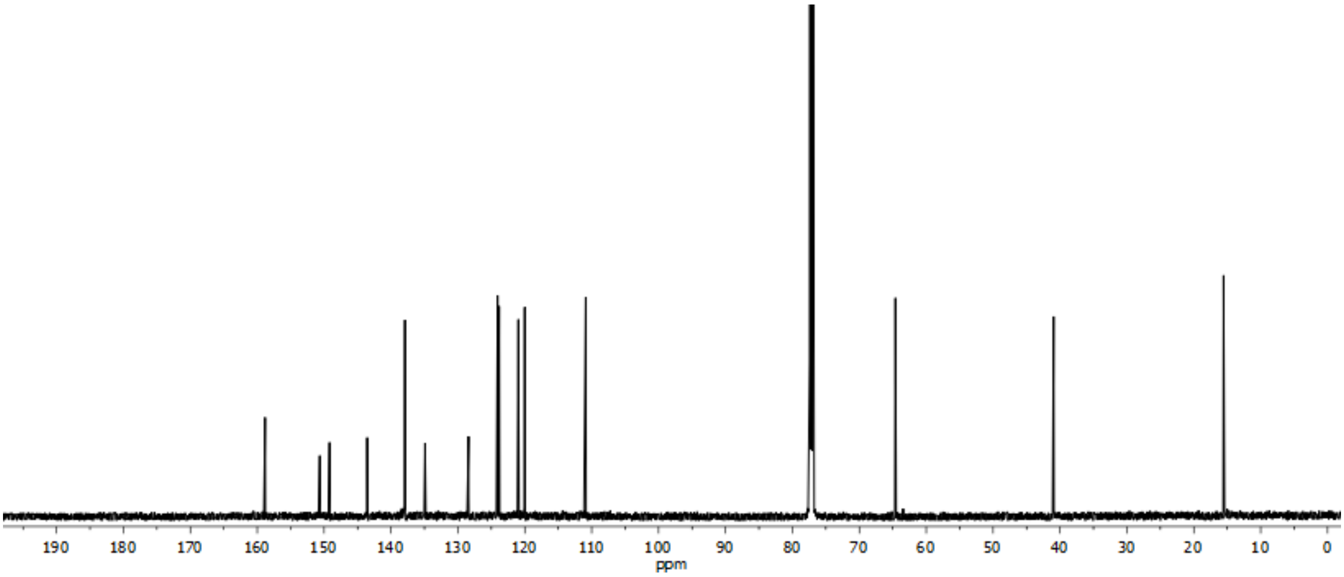

Figure S46. ^{13}C NMR (151 MHz, $CDCl_3$) spectrum of compound **6**.

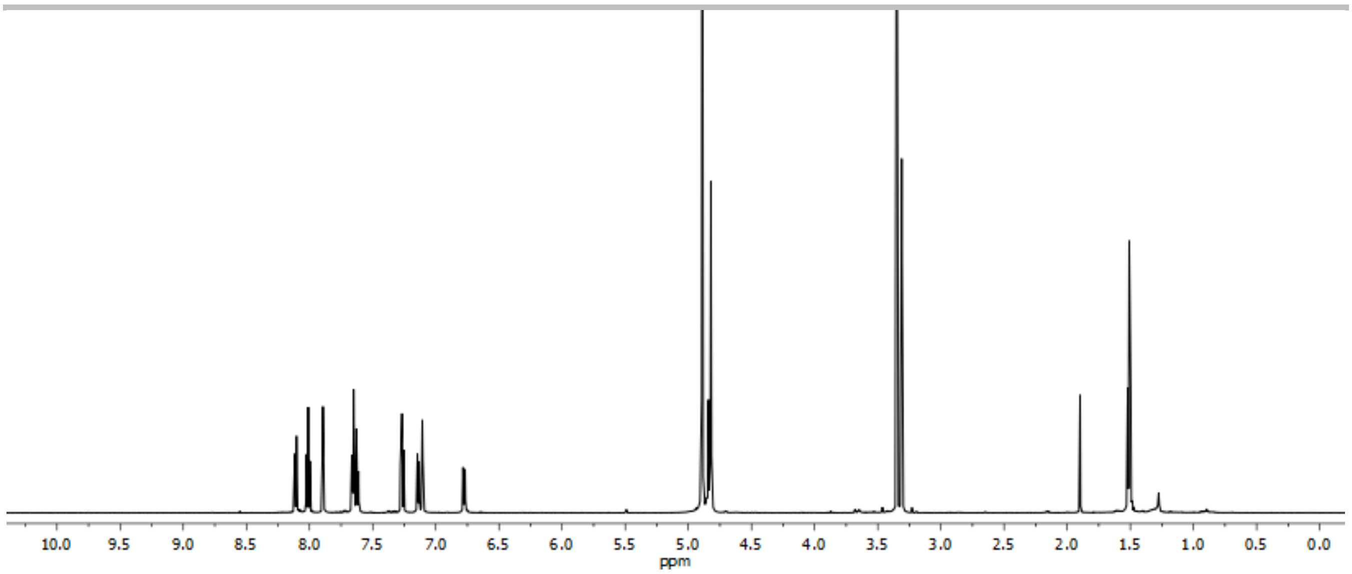

Figure S47. ^{1}H NMR (600 MHz, CD_3OD) spectrum of compound **7**.

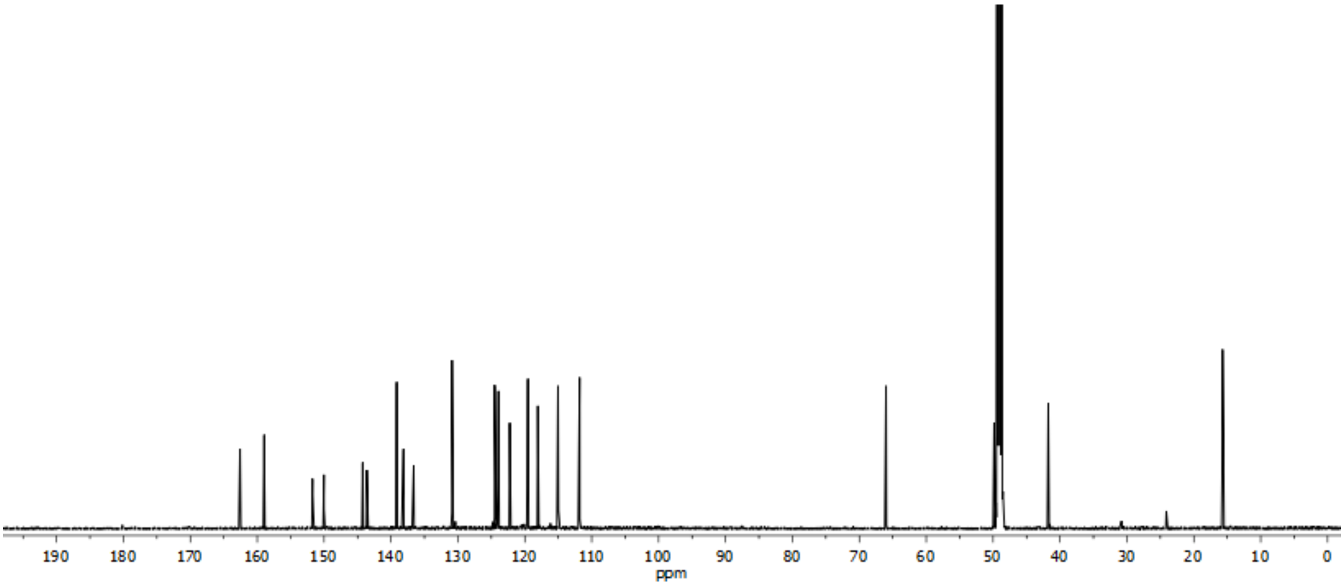

Figure S48. ^{13}C NMR (151 MHz, CD_3OD) spectrum of compound **7**.

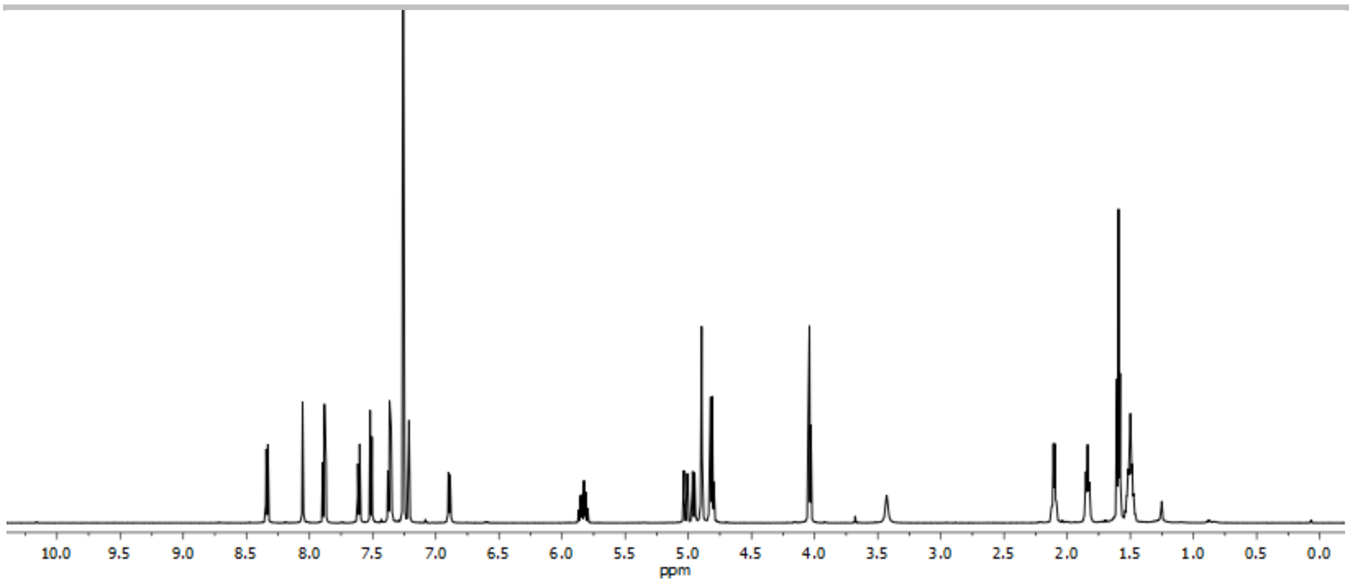

Figure S49. ^{1}H NMR (600 MHz, $CDCl_3$) spectrum of compound **8**.

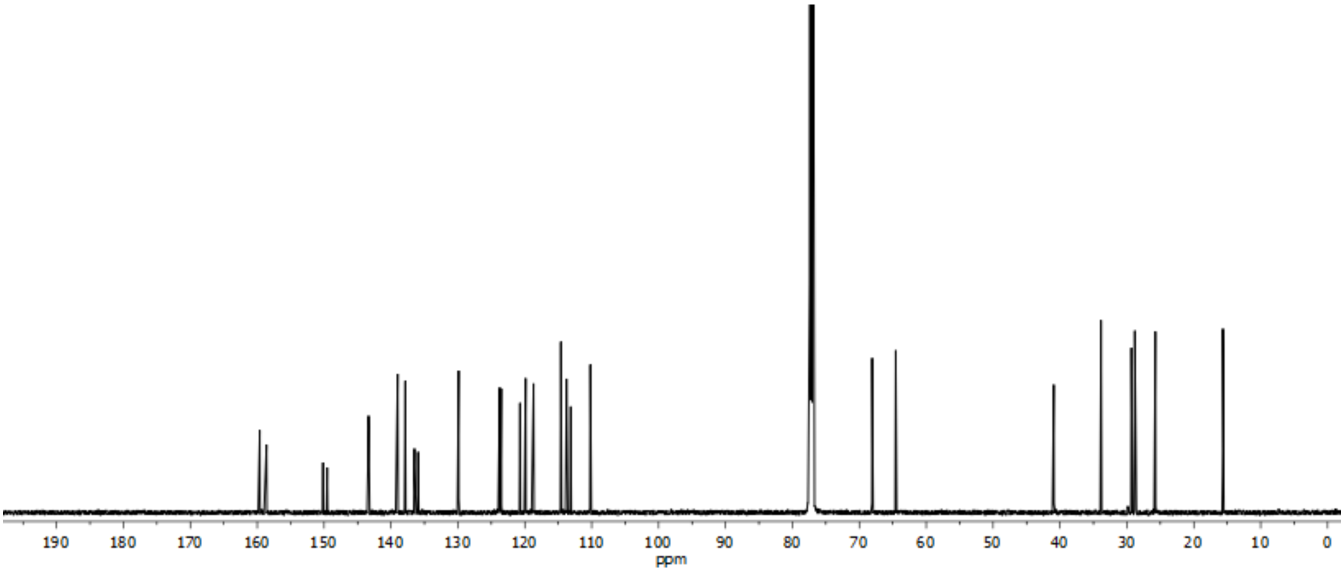

Figure S50. ^{13}C NMR (151 MHz, $CDCl_3$) spectrum of compound **8**.

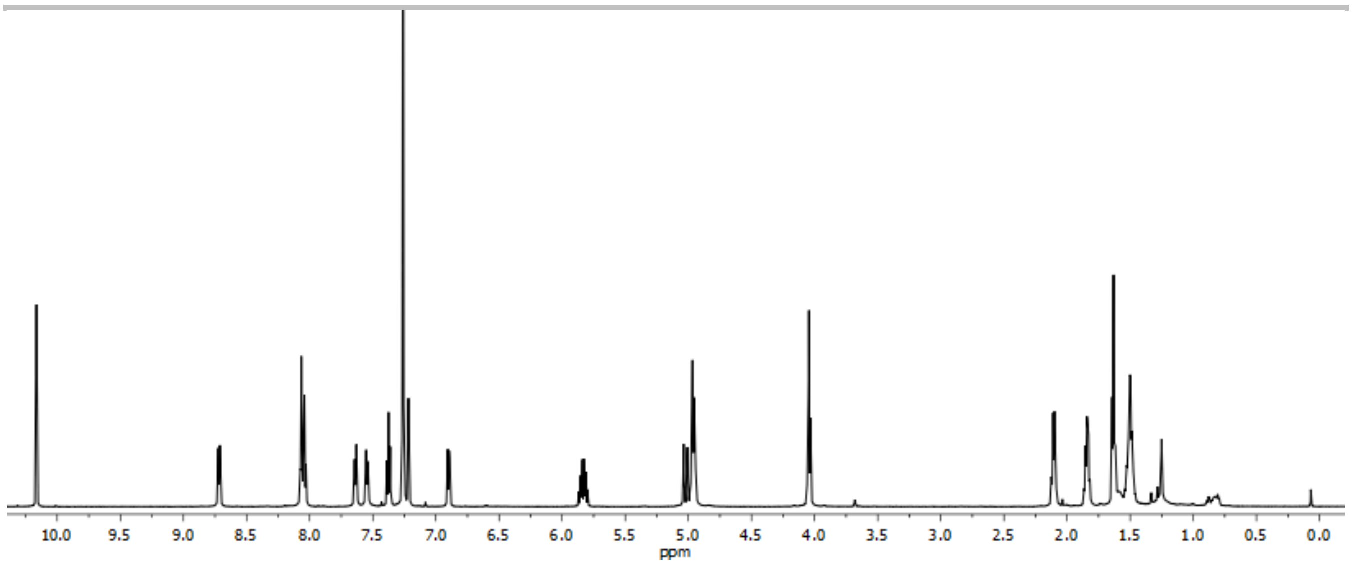

Figure S51. ^{1}H NMR (600 MHz, $CDCl_3$) spectrum of compound **9**.

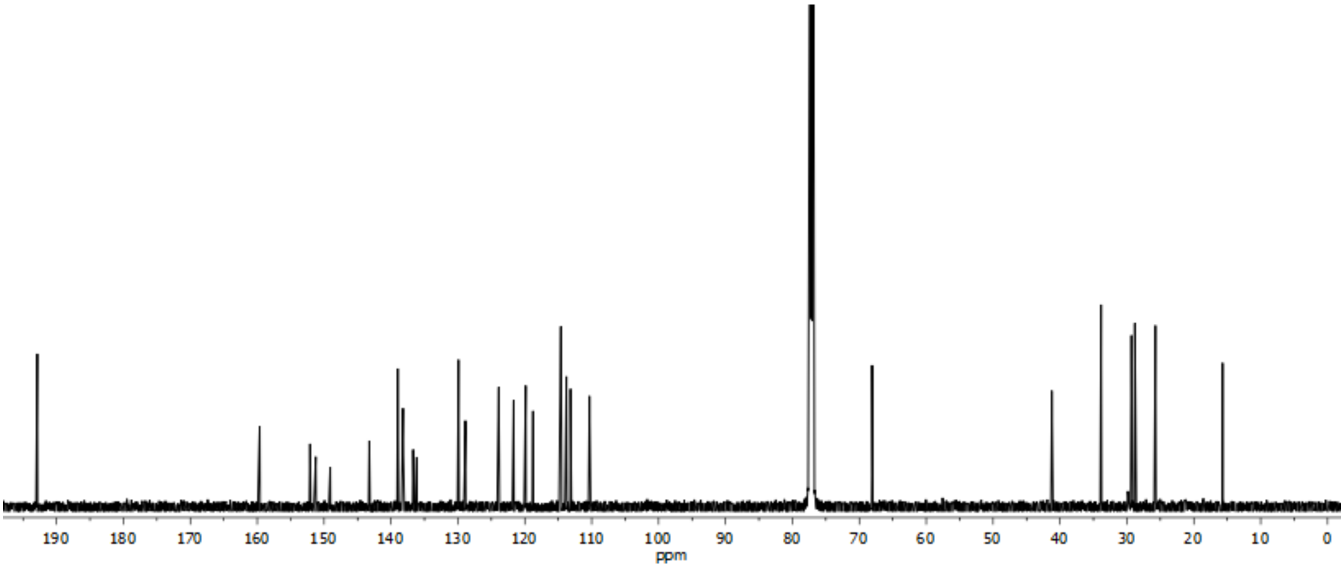

Figure S52. ^{13}C NMR (151 MHz, $CDCl_3$) spectrum of compound **9**.

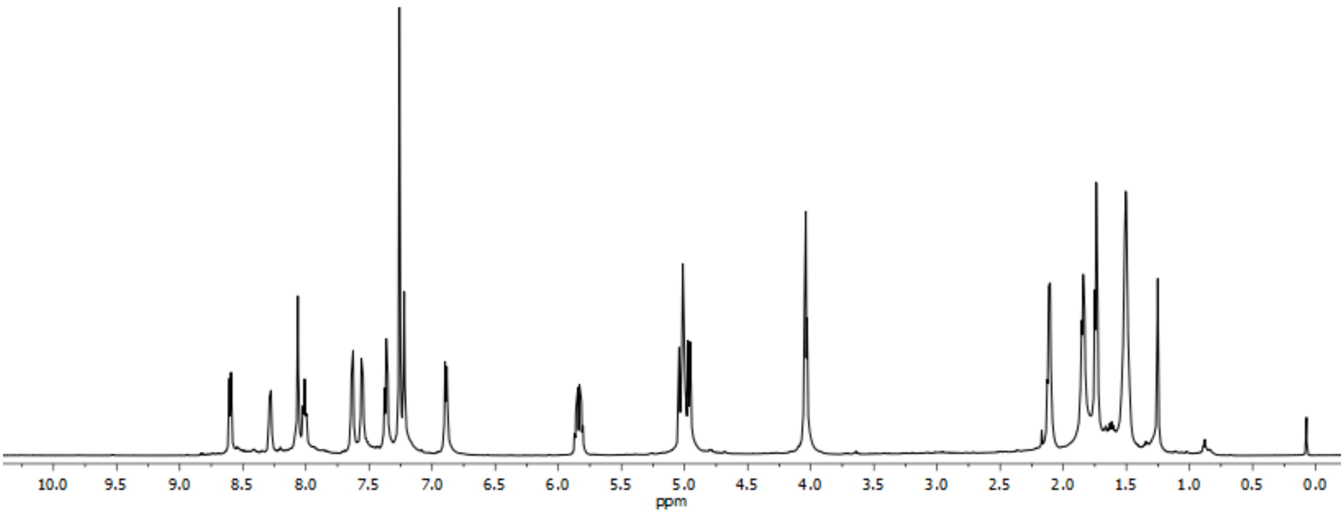

Figure S53. ^{1}H NMR (600 MHz, $CDCl_3$) spectrum of compound **1**.

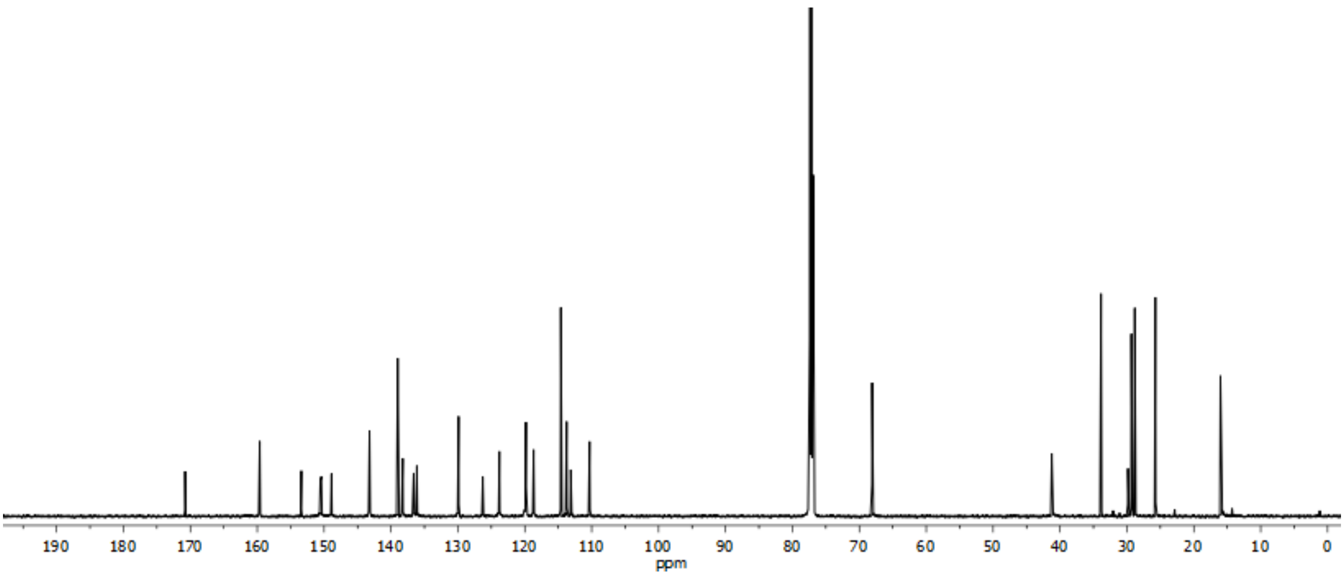

Figure S54. ^{13}C NMR (151 MHz, $CDCl_3$) spectrum of compound **1**.

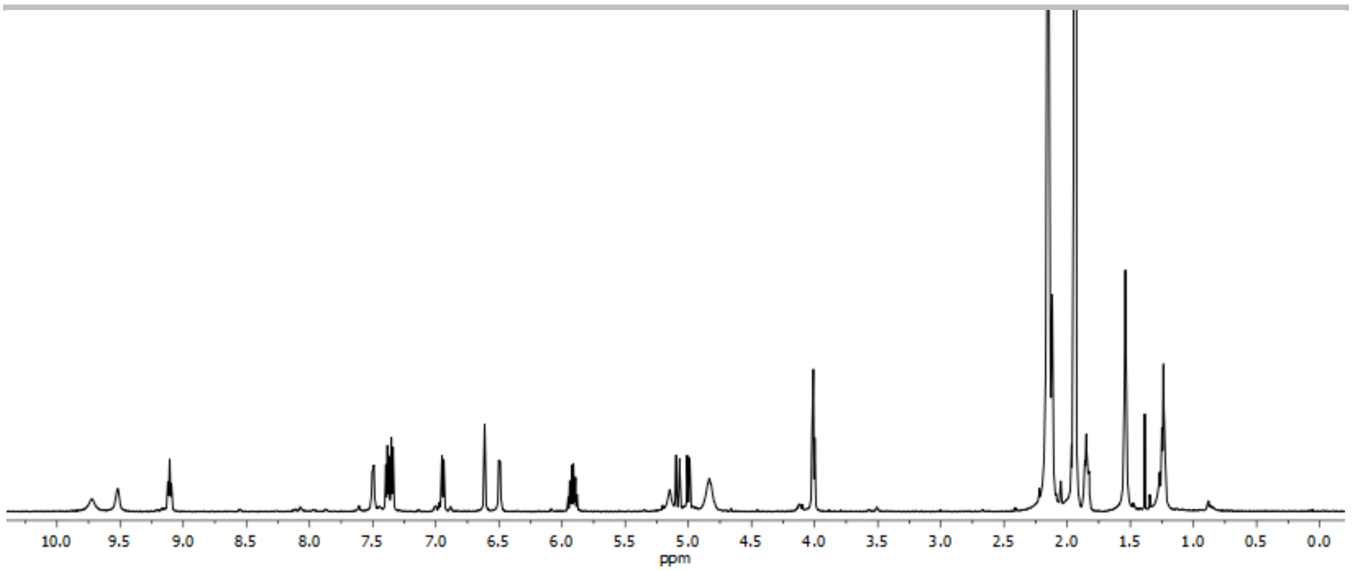

Figure S55. ^{1}H NMR (600 MHz, CD_3CN) spectrum of compound [Fe_4**1**$_4$](BF_4)$_8$.

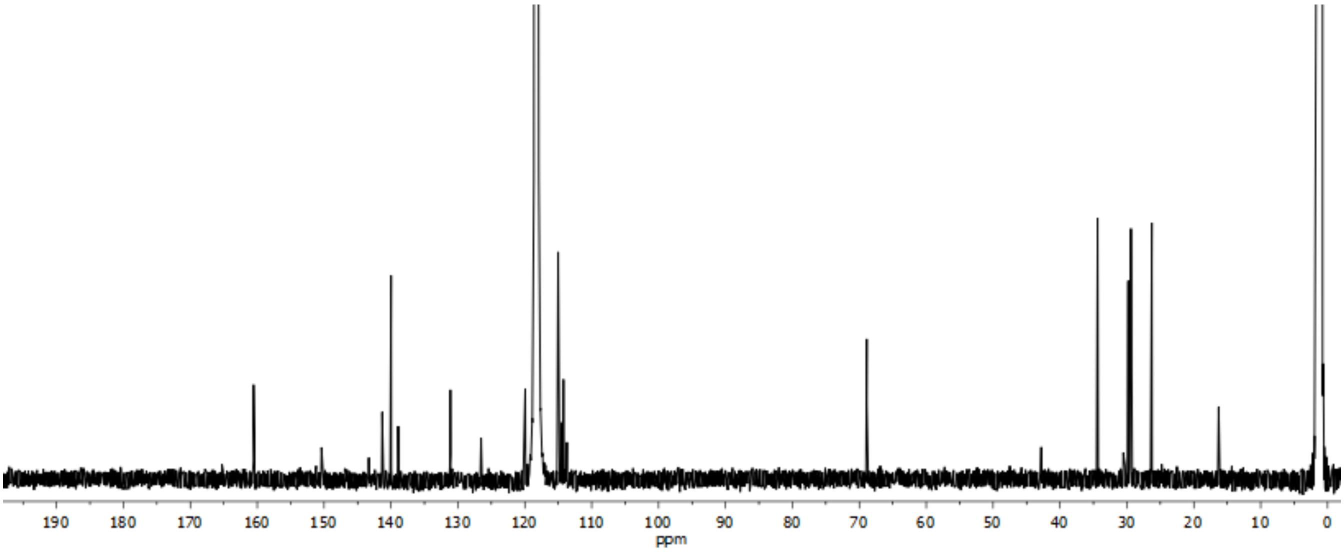

Figure S56. ^{13}C NMR (151 MHz, CD_3CN) spectrum of compound [Fe_4**1**$_4$](BF_4)$_8$.

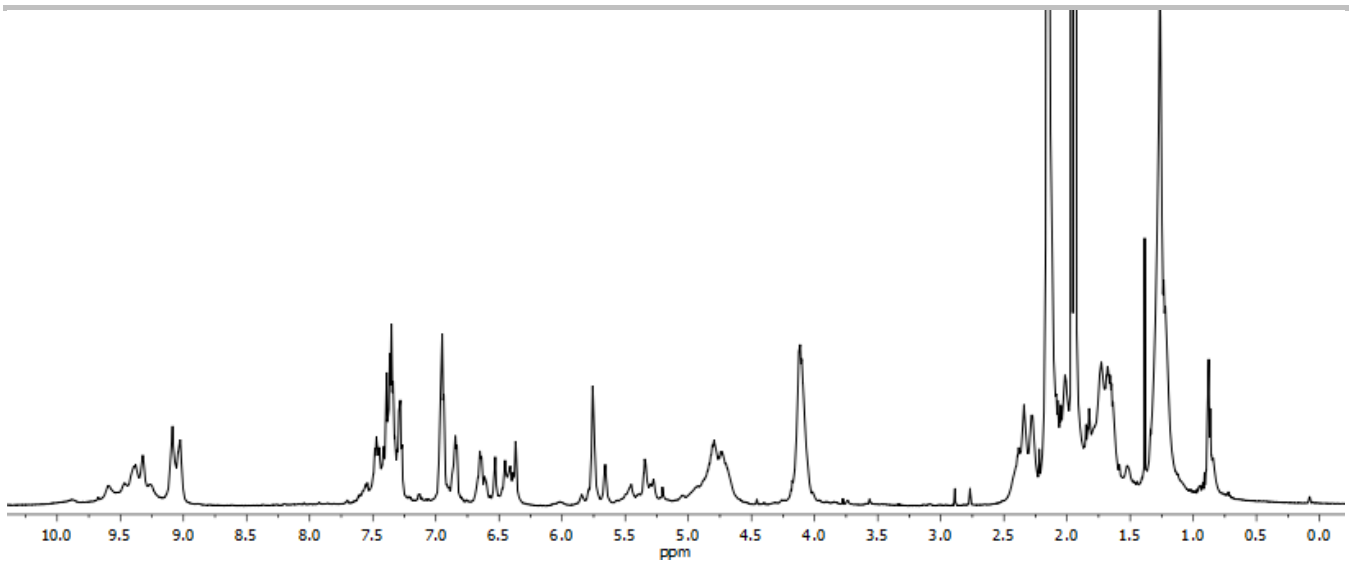

Figure S57. ^1H NMR (600 MHz, CD_3CN) spectrum of compound [Fe_4**2**/**3**]$(BF_4)_8$.

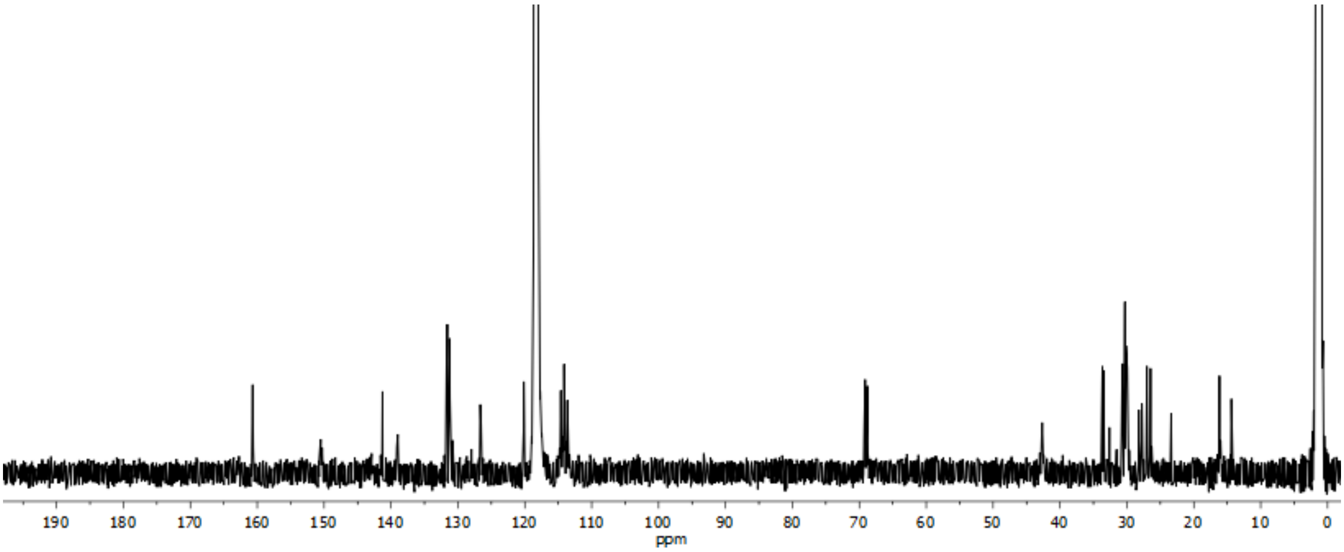

Figure S58. ^{13}C NMR (151 MHz, CD_3CN) spectrum of compound [Fe_4**2**/**3**]$(BF_4)_8$.

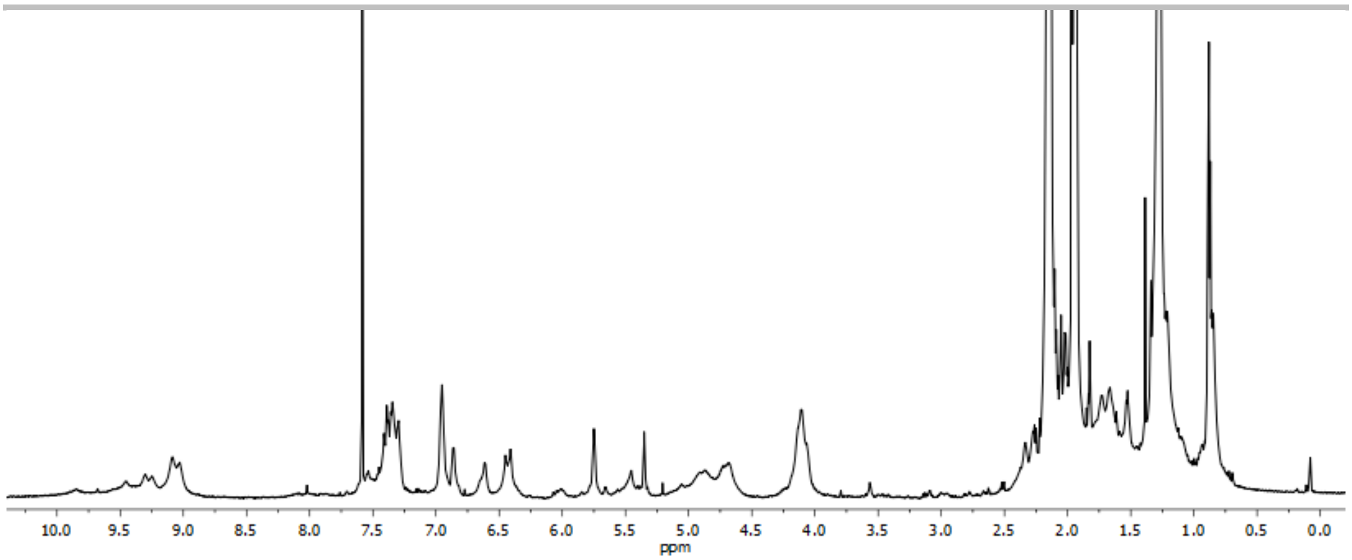

Figure S59. ^{1}H NMR (600 MHz, CD_3CN) spectrum of compound [Fe_4**2**]$(BF_4)_8$.

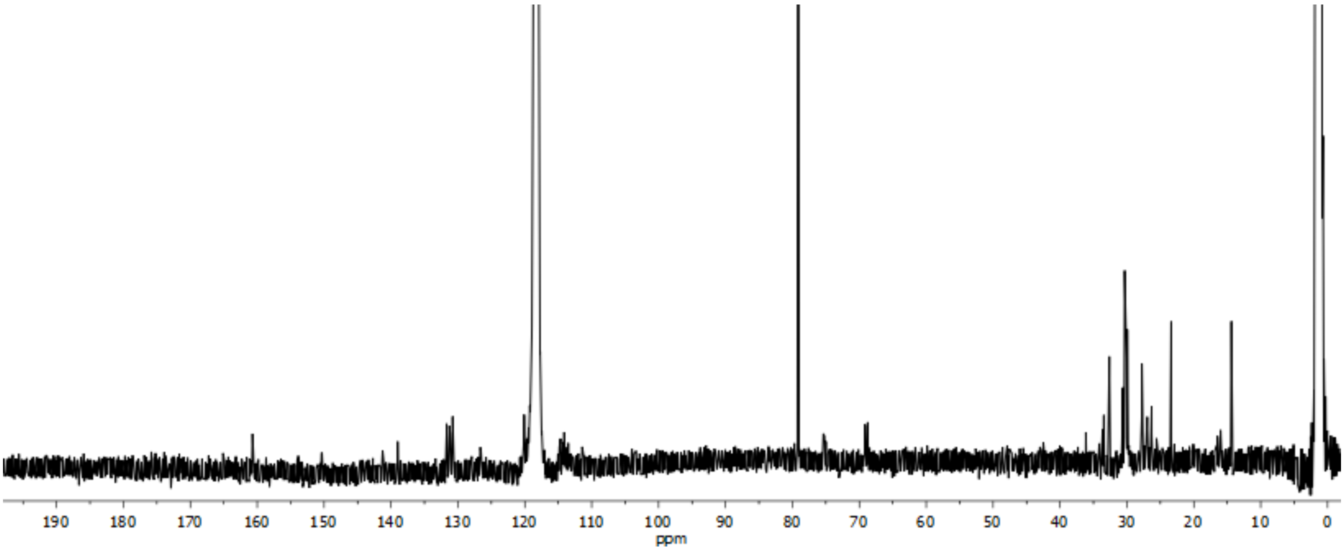

Figure S60. ^{13}C NMR (151 MHz, CD_3CN) spectrum of compound [Fe_4**2**]$(BF_4)_8$.

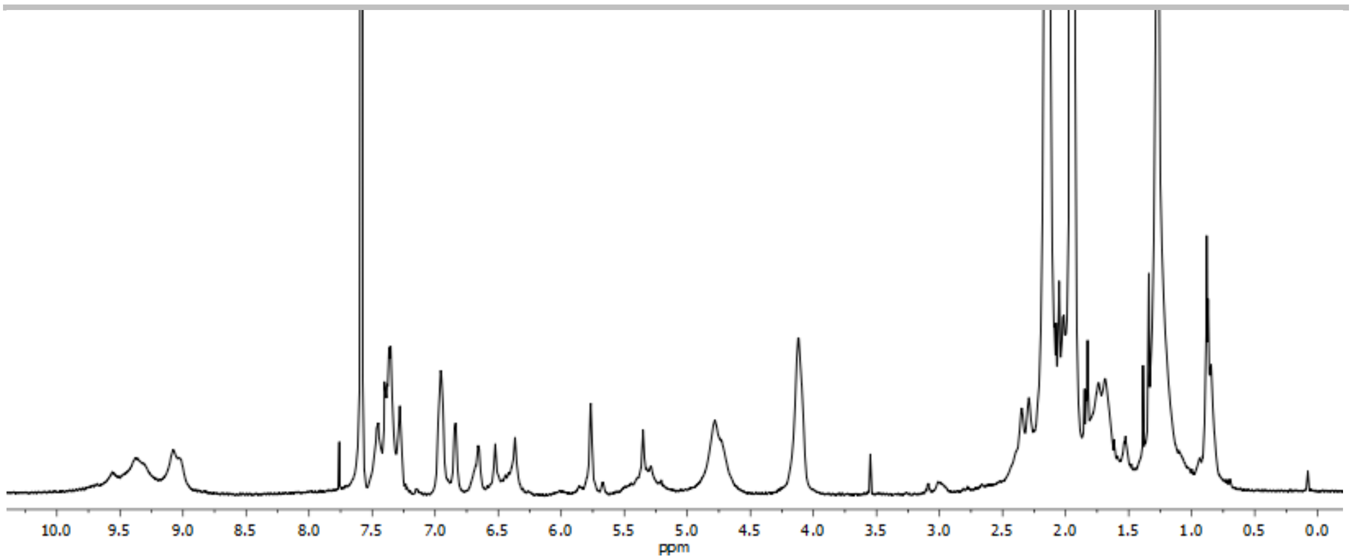

Figure S61. ^{1}H NMR (600 MHz, CD_3CN) spectrum of compound $[Fe_4\mathbf{3}](BF_4)_8$.

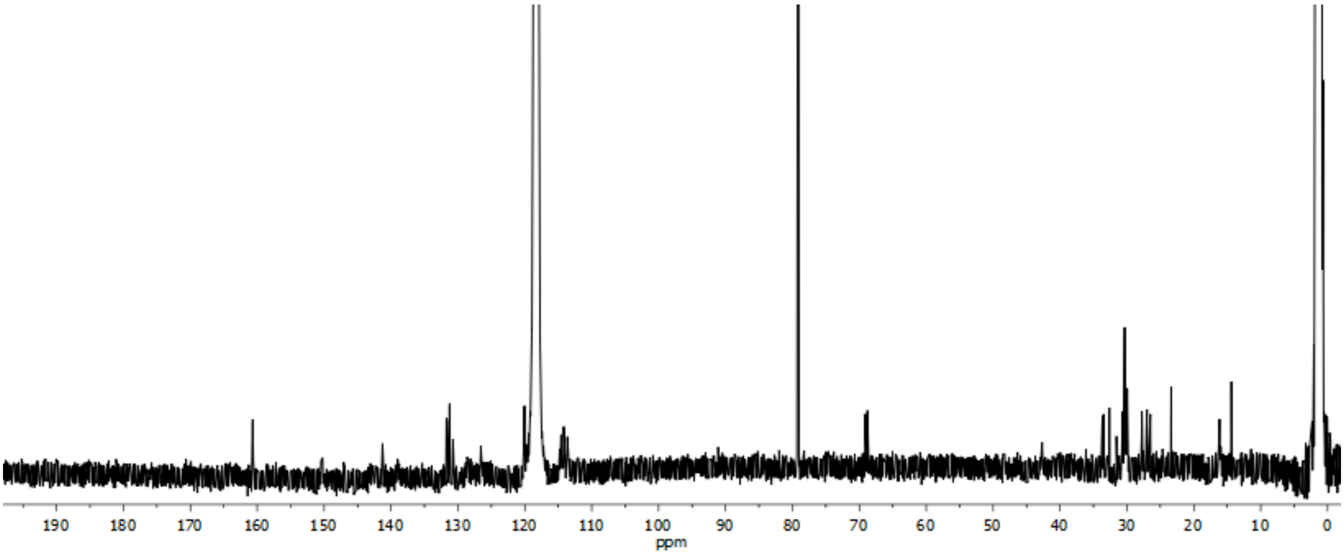

Figure S62. ^{13}C NMR (151 MHz, CD_3CN) spectrum of compound $[Fe_4\mathbf{3}](BF_4)_8$.

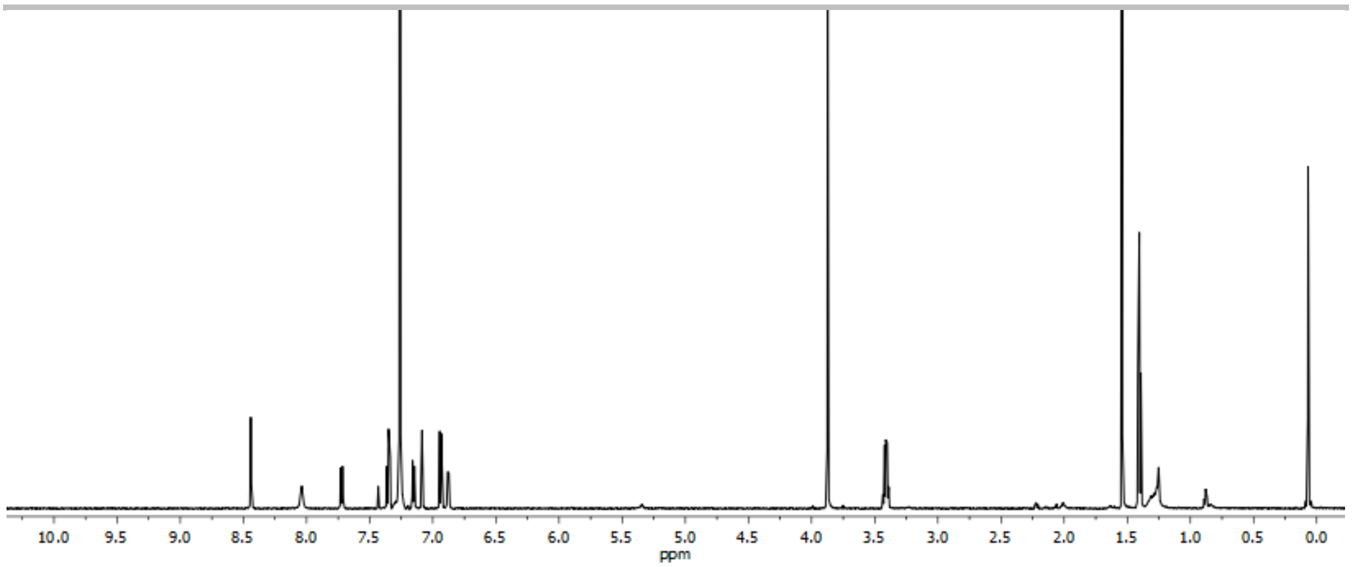

Figure S63. ^{1}H NMR (600 MHz, $CDCl_3$) spectrum of compound **10**.

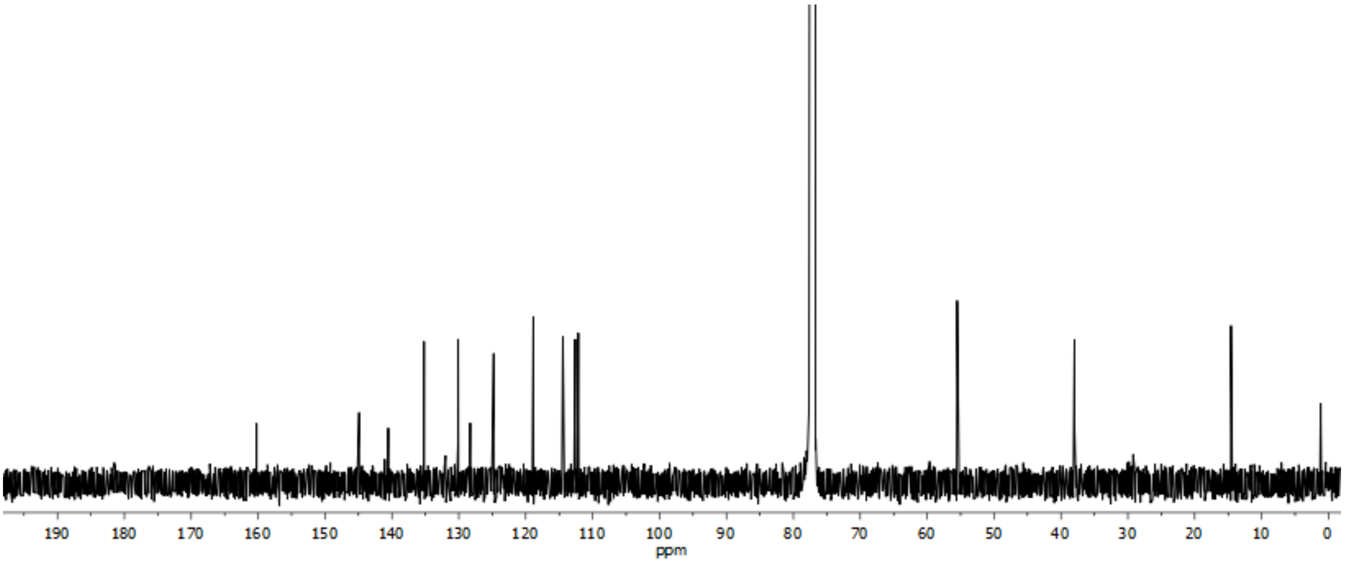

Figure S64. ^{13}C NMR (151 MHz, $CDCl_3$) spectrum of compound **10**.

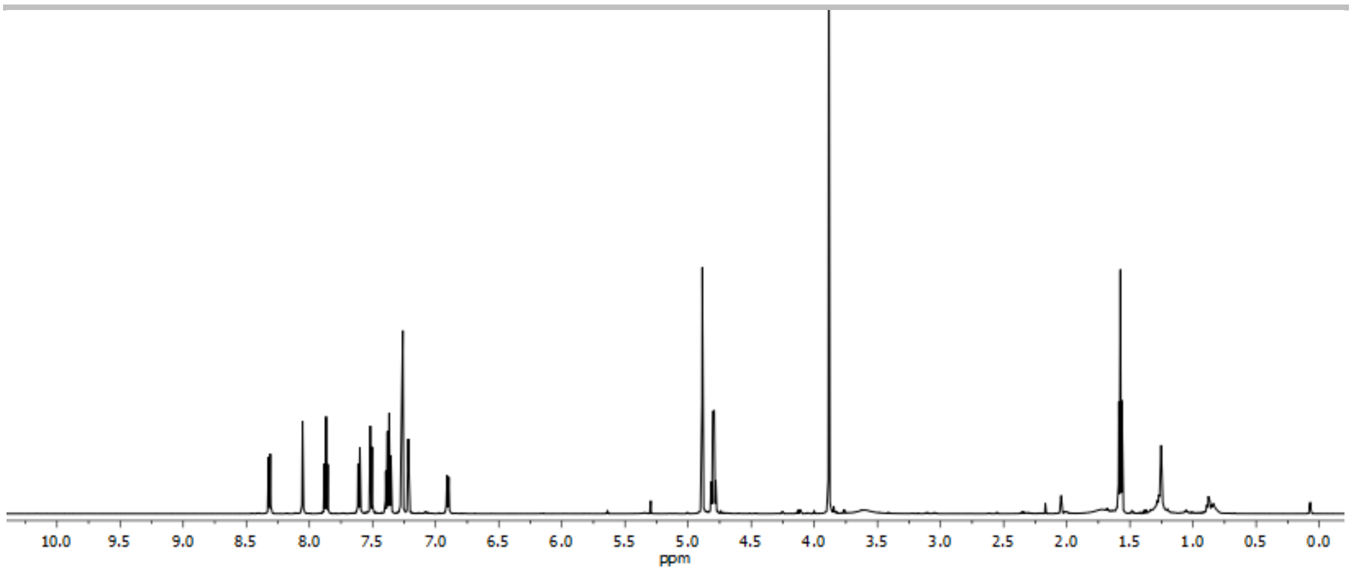

Figure S65. ^{1}H NMR (600 MHz, $CDCl_3$) spectrum of compound **11**.

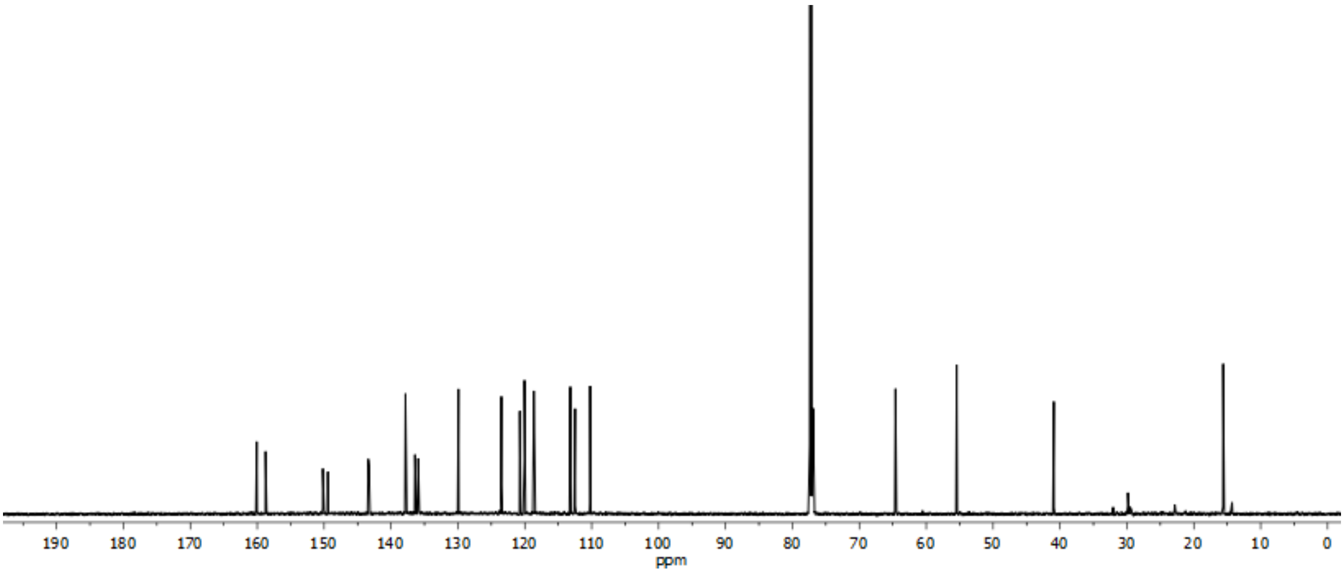

Figure S66. ^{13}C NMR (151 MHz, $CDCl_3$) spectrum of compound **11**.

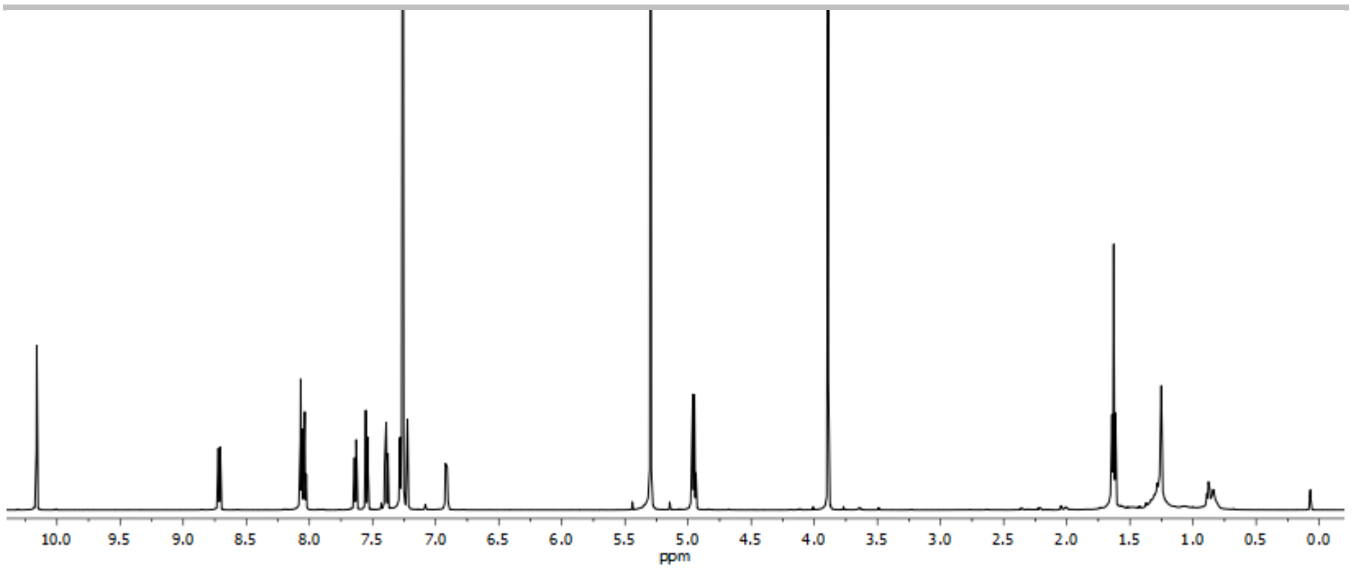

Figure S67. ^{1}H NMR (600 MHz, $CDCl_3$) spectrum of compound **12**.

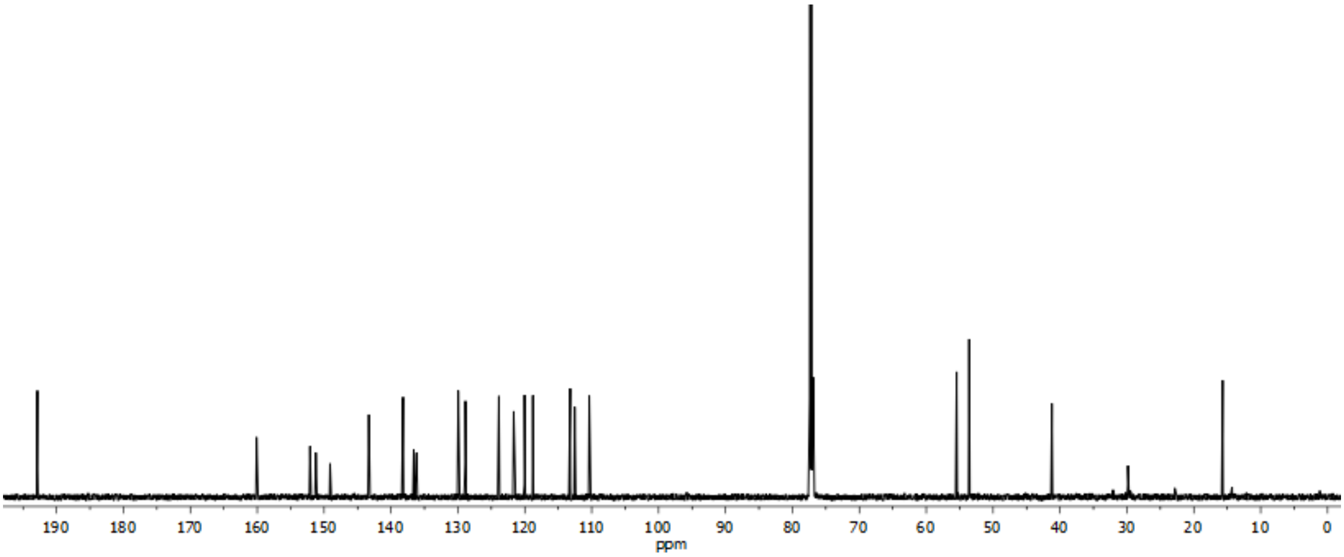

Figure S68. ^{13}C NMR (151 MHz, $CDCl_3$) spectrum of compound **12**.

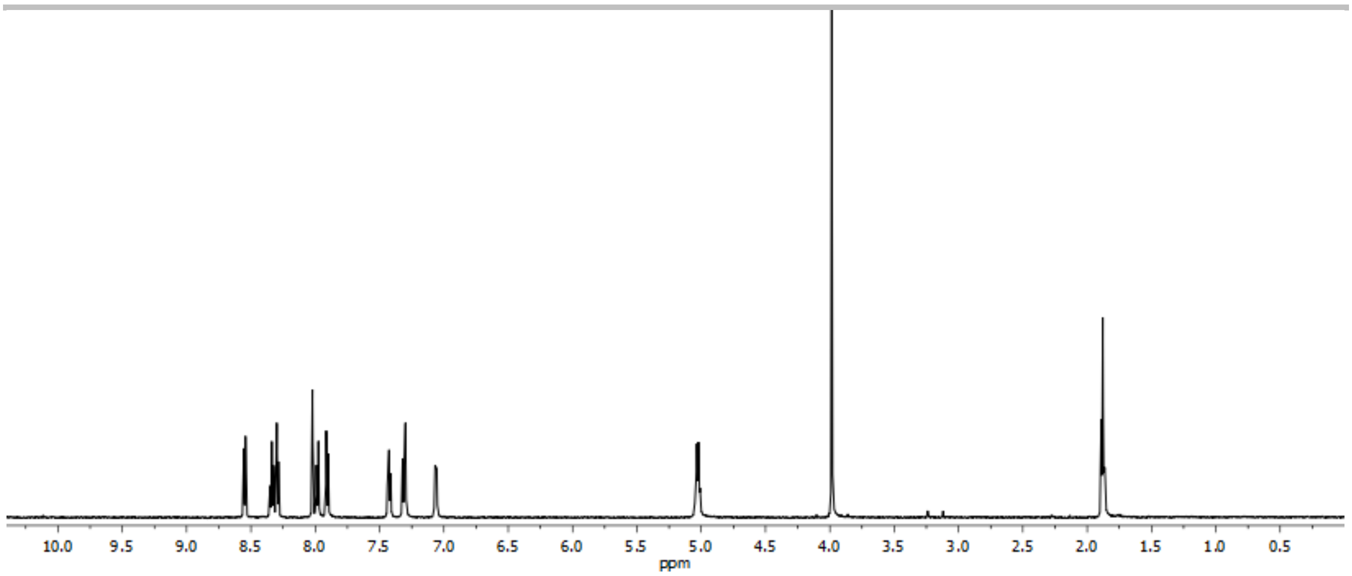

Figure S69. ^{1}H NMR (600 MHz, CF_3COOD) spectrum of compound **13**.

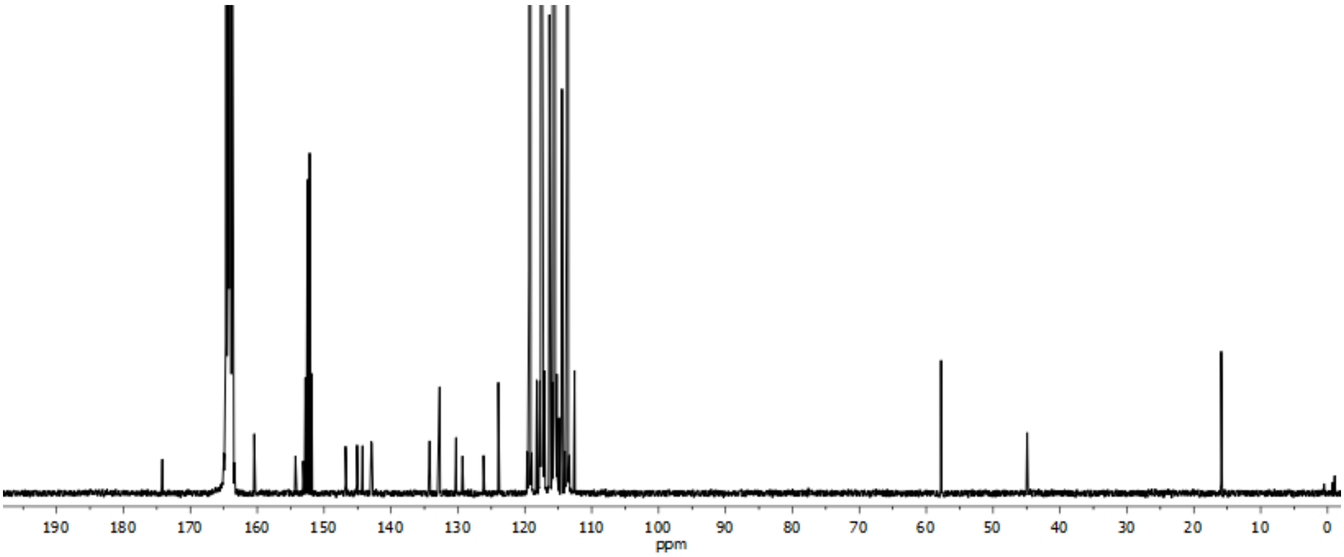

Figure S70. ^{13}C NMR (151 MHz, CF_3COOD) spectrum of compound **13**.

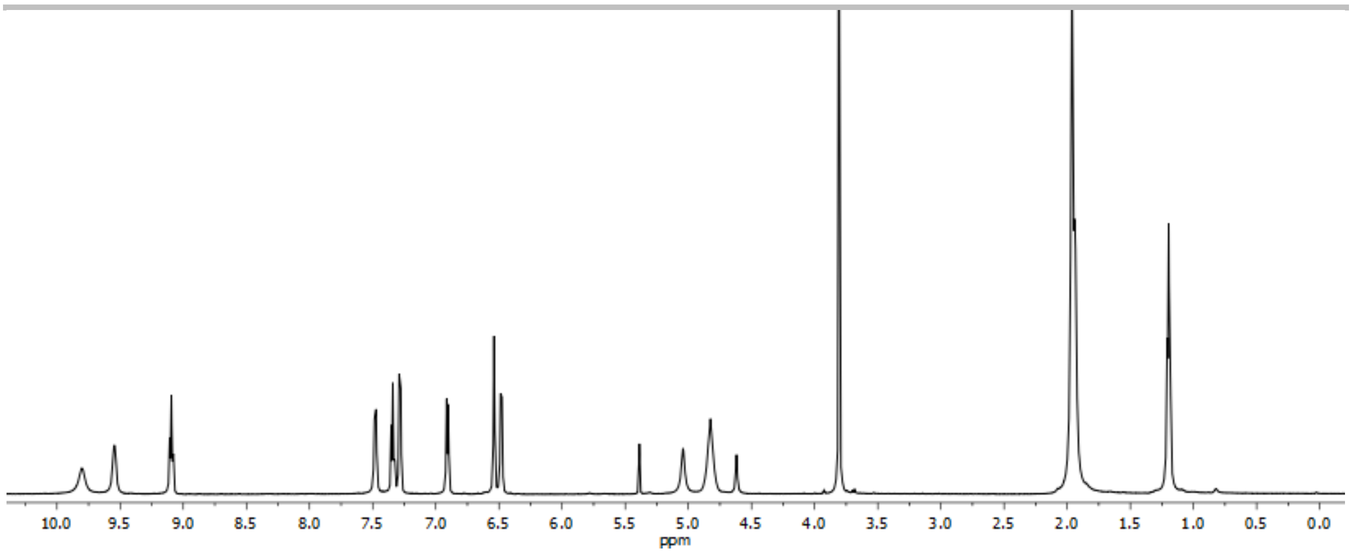

Figure S71. ^{1}H NMR (600 MHz, CD_3CN) spectrum of compound $[Fe_4\mathbf{13}_4](BF_4)_8$.

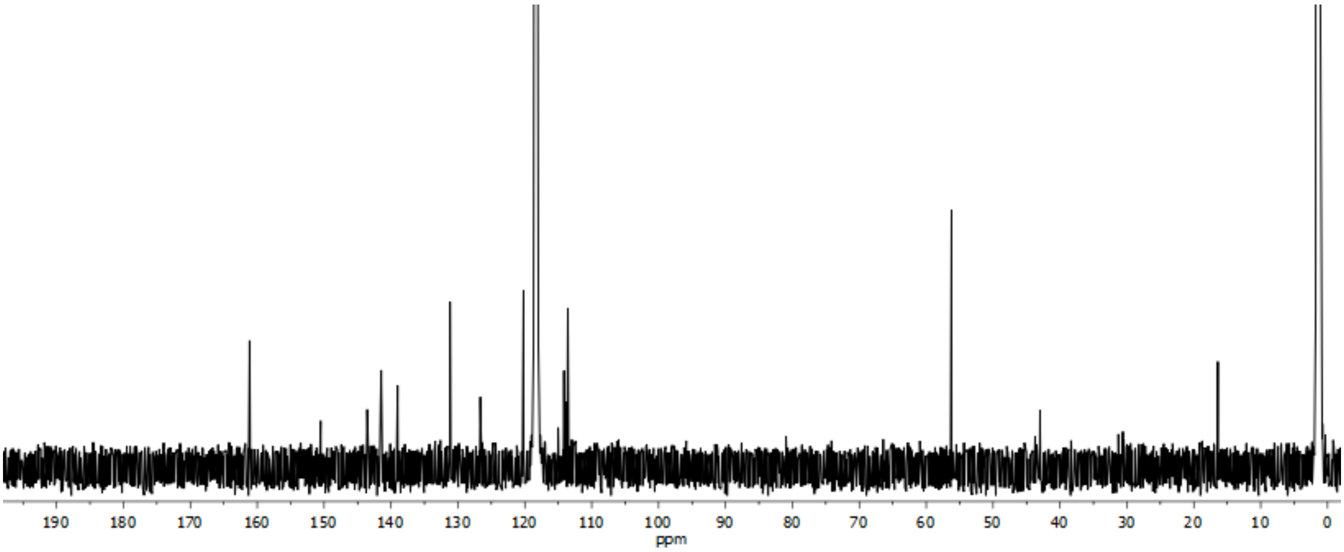

Figure S72. ^{13}C NMR (151 MHz, CD_3CN) spectrum of compound $[Fe_4\mathbf{13}_4](BF_4)_8$.

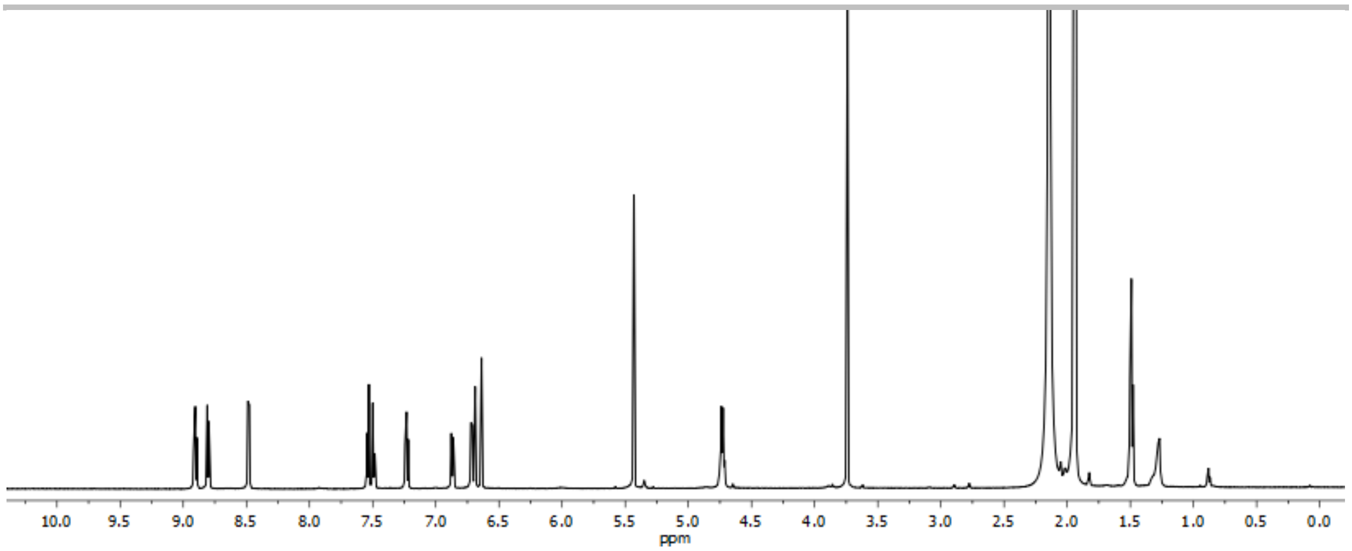

Figure S73. ^{1}H NMR (600 MHz, CD_3CN) spectrum of compound $[Zn_4\mathbf{13}_4](BF_4)_8$.

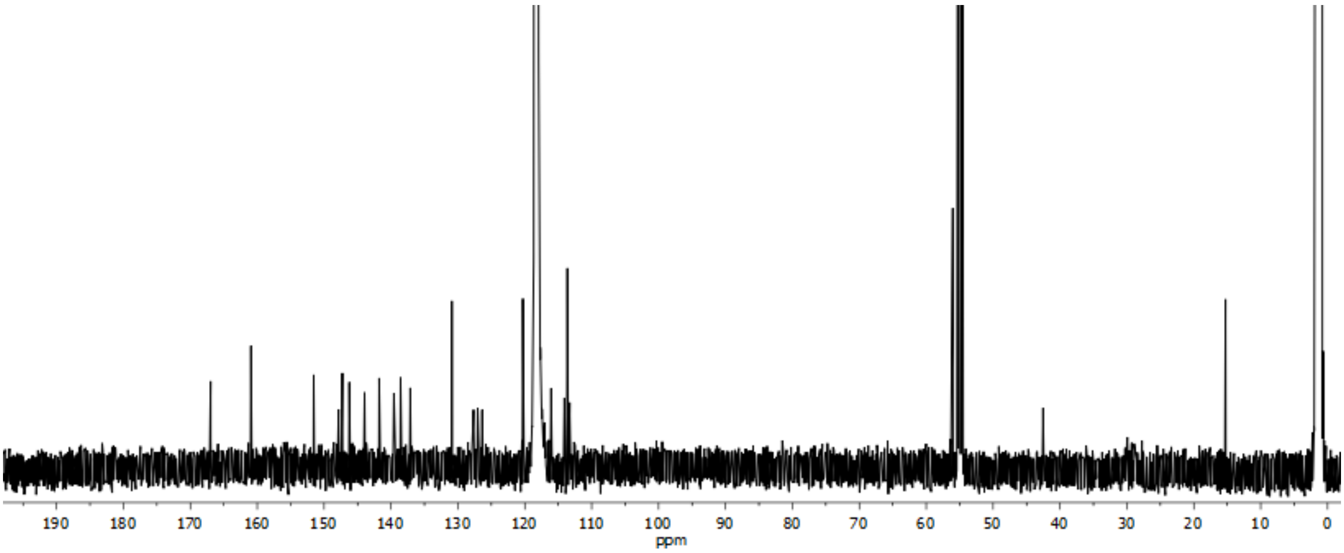

Figure S74. ^{13}C NMR (151 MHz, CD_3CN) spectrum of compound $[Zn_4\mathbf{13}_4](BF_4)_8$.

5. References

[S1] R. Ziessel, P. Nguyen, L. Douce, M. Cesario, C. Estournes, *Org. Lett.* **2004**, *6*, 2865-2868.

[S2] H. Nowell, S. A. Barnett, K. E. Christensen, S. J. Teat, D. R. Allan, *J. Synchrotron Radiat.* **2012**, *19*, 435-441.

[S3] a) G. M. Sheldrick, *SADABS*, empirical absorption correction program based upon the method of Blessing; b) L. Krause, R. Herbst-Irmer, G. M. Sheldrick, D. Stalke, *J. Appl. Cryst.* **2015**, *48*; c) R. H. Blessing, *Acta Crystallogr.* **1995**, *A51*, 33-38.

[S4] a) G. M. Sheldrick, *Acta Crystallogr.* **2015**, *C71*, 3-8; b) O. V. Dolomanov, L. J. Bourhis, R. J. Gildea, J. A. K. Howard, H. Puschmann, *J. Appl. Cryst.* **2009**, *42*, 339-341.

[S5] J. E. Beves, J. J. Danon, D. A. Leigh, J.-F. Lemonnier, I. J. Vitorica-Yrezabal, *Angew. Chem. Int. Ed.* **2015**, *54*, 7555-7559; *Angew. Chem.* **2015**, *127*, 7665-7669.
